# Design, synthesis, and biological evaluation of potent FAK-degrading PROTACs

**DOI:** 10.1080/14756366.2022.2100886

**Published:** 2022-08-17

**Authors:** Qiaohua Qin, Ruifeng Wang, Qinglin Fu, Guoqi Zhang, Tianxiao Wu, Nian Liu, Ruicheng Lv, Wenbo Yin, Yin Sun, Yixiang Sun, Dongmei Zhao, Maosheng Cheng

**Affiliations:** a Key Laboratory of Structure-Based Drug Design and Discovery, Ministry of Education, School of Pharmaceutical Engineering, Shenyang Pharmaceutical University, Shenyang, PR China; b Department of Pharmacy, Shanxi Medical University, Taiyuan, PR China

**Keywords:** FAK, PROTACs, PF-562271, A549 cells, protein degradation

## Abstract

FAK mediated tumour cell migration, invasion, survival, proliferation and regulation of tumour stem cells through its kinase-dependent enzymatic functions and kinase-independent scaffolding functions. At present, the development of FAK PROTACs has become one of the hotspots in current pharmaceutical research to solve above problems. Herein, we designed and synthesised a series of FAK-targeting PROTACs consisted of PF-562271 derivative **1** and Pomalidomide. All compounds showed significant *in vitro* FAK kinase inhibitory activity, the IC_50_ value of the optimised PROTAC **A13** was 26.4 nM. Further, **A13** exhibited optimal protein degradation (85% degradation at 10 nM). Meantime, compared with PF-562271, PROTAC **A13** exhibited better antiproliferative activity and anti-invasion ability in A549 cells. More, **A13** had excellent plasma stability with *T*_1/2_ >194.8 min. There are various signs that PROTAC **A13** could be useful as expand tool for studying functions of FAK in biological system and as potential therapeutic agents.

## 1. Introduction

Focal adhesion kinase (FAK), also named protein tyrosine kinase 2 (PTK2), was first discovered in 1992 by Schaller et al^1^. As an intracellular non-receptor tyrosine kinase, FAK can be divided into four parts: the middle kinase catalytic domain, the amino-terminal FERM (4.1-ezrin-radixin-moesin) domain, the carboxy-terminal focal adhesion targeting (FAT) domain and proline-rich regions (PRRs). FAK protein contains at least 6 tyrosine phosphorylation sites (Tyr397, Tyr407, Tyr576, Tyr577, Tyr861, and Tyr925), which are distributed in kinase catalytic domain and other scaffolding domain of FAK. The phosphorylation of these tyrosine residues provided a binding site for recruiting effector protein, playing an pivotal role in regulating the biological functions of FAK^2^. The kinase domain was mainly responsible for regulating the kinase activity of FAK, while the FERM, FAT and PRR domains regulated protein-protein interactions, followed by the activation of downstream signalling. Hence, FAK exerted catalytic and scaffolding functions, both of them were essential in early embryonic development, reproduction, cancer development, etc^3–6^. Overexpression of FAK had been detected in multiple tumour types, and biological studies revealed that FAK was essential for the development and progression of various human cancers^7–10^. Thus, the FAK has emerged as a promising target for anti-cancer therapy.

To date, several potent FAK inhibitors have been developed, and some of them were conducted clinical evaluation. GSK-2256098, VS-4718, PF-562271 and Defactinib are ATP-competitive inhibitors of kinase domain. GSK-2256098 is a highly selective inhibitor of FAK and has been shown to efficiently inhibit the phosphorylation of Tyr397^11^. VS-4718 is used to treat metastatic non-hematological malignancies, advanced non-hematological malignancies or advanced pancreatic cancer of patients in combination with gemcitabine or nabutaxel^12^. PF-562271 is a highly active and highly selective FAK inhibitor developed by Pfizer^13^. Defactinib is evaluated to treat ovarian cancer, pancreatic cancer, non-small cell lung cancer and mesothelioma in combination with checkpoint inhibitors^14^. As we said above, FAK exerts kinase-dependent enzymatic functions and kinase-independent scaffolding functions. The development of the small-molecule inhibitors can inhibit the enzymatic functions of FAK, but can’t prevent the kinase-independent scaffolding functions. In addition, small molecule drugs are likely to cause off-target toxicity and even drug resistance at high concentrations. In summary, it is necessary to develop a strategy against both the enzymatic functions and scaffolding functions of FAK.

**Figure 1.**
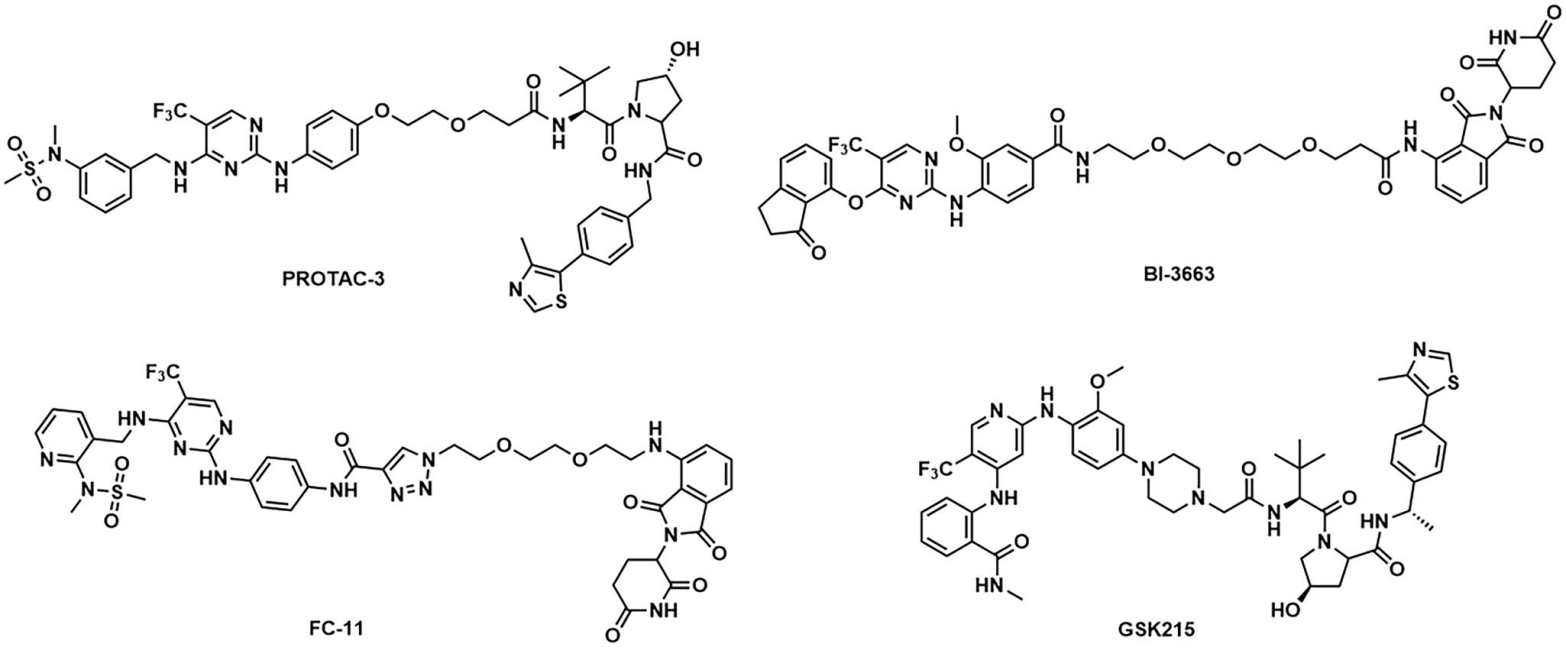
Structures of representative FAK degraders.

Proteolysis targeting chimaeras (PROTACs) are a class of heterobifunctional molecules containing three moieties: the ligand of the protein of interest (POI), the ligand of E3 ubiquitin ligase (E3 ligase) and the linker^15^. The PROTAC molecules formed a POI-PROTAC-E3 ligase ternary complexes after entering the cell, resulting in ubiquitination of the target protein and consequently proteasome-mediated degradation^16^. PROTACs are not required to occupy binding sites for prolonged periods of time during degradation and can function multiple times in the cell, which means that only a catalytic amount of the PROTAC molecule is needed to degrade the target protein. More importantly, PROTAC can inhibit both kinase-dependent enzymatic functions and the essential functions mediated by the FAK scaffolding moieties (Figure 1).

Several targeting-FAK PROTACs have been successfully developed (Figure 1). In 2018, Crew’s group reported PROTAC-3 as a selective and potent Fak degrader, which can potentially induce the degradation of FAK in human prostate cancer cell line PC3 cells with a DC_50_ value of 3.0 nM^17^. Using FAK inhibitor BI-4644 and VHL E3 ligase binder, PROTAC BI-3663 was designed by Boehringer Ingelheim, which can effectively degrade FAK protein in 12 cell lines. However, BI-3663 did not display more potent antiproliferative activity than BI-4644^18^. Rao et al. identified that the degradation of FAK induced by FC-11 can be restored if PROTAC molecules were removed^19^^,^^20^. GlaxoSmithKline reported GSK215 as a potent and selective FAK-degrading PROTAC, which induced rapid and prolonged FAK degradation with a single dose (8 mg/kg) in mice^21^^,^^22^. FAK PROTACs with different E3 ligase ligands and different FAK inhibitors are valuable to enhance activities, drug-like properties, and exploration of structure-activity relationships (SARs). Therefore, the research of novel PROTAC molecules targeting FAK kinase still has important academic significance and practical application requirements.

**Figure 2.**
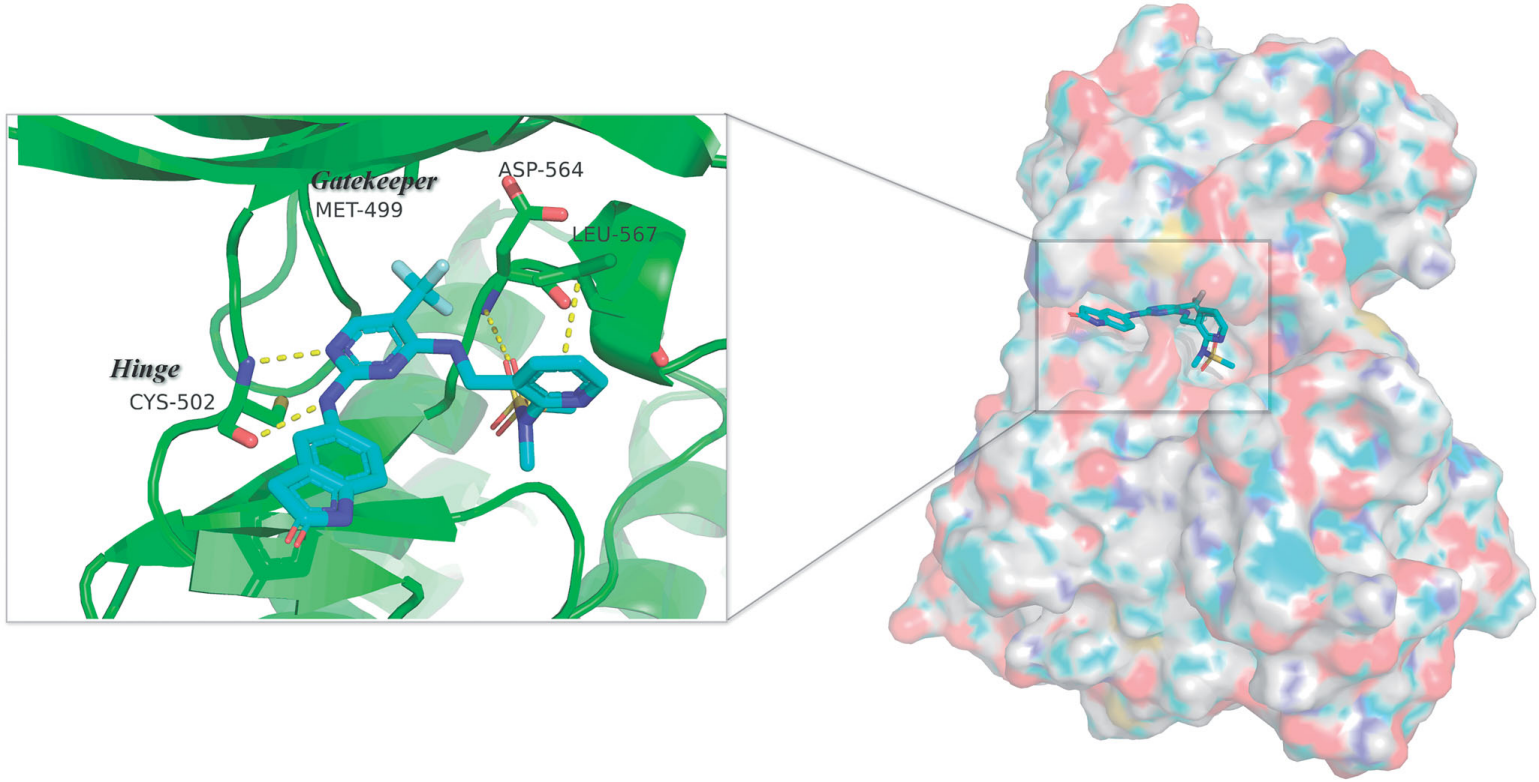
Three-dimensional space matching diagram and detailed interactions of PF-562271 in the ATP-binding site of FAK (PDB ID: 3BZ3). The interactions are illustrated with yellow dashed lines.

PF-562271, a highly active and highly selective FAK inhibitor, was tolerated well in phase I clinical trials. In this study, we presented a series of FAK PROTACs based on PF-562271 and CRBN E3 ligase ligand Pomalidomide. The co-crystal of PF-562271 and FAK was analysed (PDB ID: 3BZ3), as shown in Figure 2, the 2,4-diaminopyrimidine core formed two donor-acceptor interactions with Cys502 in the hinge region, *N*-methyl sulphonamide fragment formed a hydrogen bond interaction with Asp564 in the DFG-motif, the trifluoromethyl group contained a hydrophobic interaction with the gatekeeper residue Met499, the lactam fragment extended to the solvent region and could be modified to bind to the linkers.

**Figure 3.**
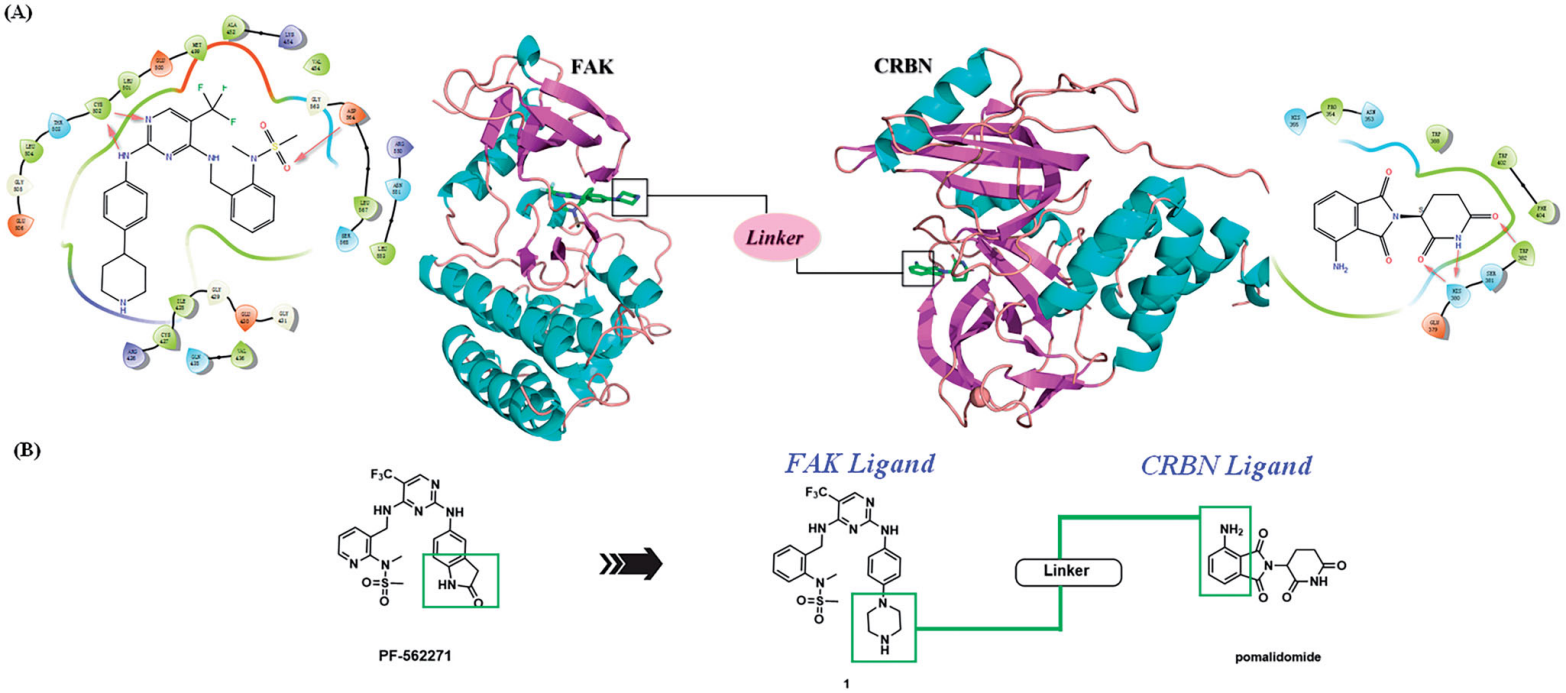
Design of FAK-targeting PROTACs. (**A**) Molecular docking model of compound **1** with FAK protein (PDB ID: 3BZ3) and the co-crystal binding modes of Pomalidomide (PDB ID: 4CI3). Piperazine fragment of **1** with CRBN E3 ligase ligand to develop FAK PROTACs by linkers. (**B**) FAK-targeting PROTACs including three parts: FAK Ligand, Linker, and CRBN Ligand.

The lactam fragment of PF-562271 was replaced with a piperazine ring, leaving a binding site for the linkers. At the same time, the pyridine ring was replaced with a benzene ring to obtain compound **1**. *In vitro* enzymatic activity of compound **1** remained comparable to that of PF-562271. A docking study was conducted to analyse the binding mode of compound **1** and FAK. The results of molecular docking were shown in Figure 3(A). The 2,4-diaminopyrimidine core formed two donor-acceptor interactions with Cys502 in the hinge region, *N*-methyl sulphonamide fragment formed a hydrogen bond interaction with Asp564 in the DFG-motif, the trifluoromethyl group contained a hydrophobic interaction with the gatekeeper residue Met499, the piperazine ring of compound **1** extended to the solvent region to connect with the linkers. Pomalidomide was an inhibitor of CRBN developed by Celgene for the treatment of multiple myeloma, and had been widely used in the design of PROTACs because of low molecular weight. The co-crystal of pomalidomide (PDB ID: 4CI3) with CRBN protein was analysed, as shown in Figure 3(A), piperidine-2,6-dione formed three donor-acceptor interactions with His380 and Trp382, the amino-substituted benzene ring moiety was exposed in the solvent region, serving as the attachment site for the linkers. Based on the above strategies, we designed 15 FAK-targeting PROTACs based on PF-562271 and Pomalidomide, and alkyl-chains or PEG-chains of different lengths and compositions as the linkers (Figure 3(B)). Further, the synthesis and biological evaluation of the designed FAK PROTACs were exhibited in this paper.

## 2. Experimental section

The starting materials, reagents and solvents were commercially available products without further purification unless otherwise. The organic solvents used are purified and preserved by conventional methods. The reaction was monitored by thin-layer chromatography (TLC) on HSGF-254 (10–40 μm) silica gel plates and visualised with UV light. Column chromatography was performed on silica gel (200–300 mesh ASTM). Nuclear magnetic resonance (NMR) data were recorded in DMSO-d_6_ or CDCl_3_ on Bruker ARX-600 NMR or Bruker ARX-400 NMR spectrometers with TMS as an internal standard. LC-MS analysis was performed using an Agilent 1200 liquid phase mass spectrometer (ESI mode). High-resolution accurate mass spectrometry (HRMS) determinations for all final target compounds were obtained on a Bruker micromass time of flight mass spectrometer equipped with an electrospray ionisation (ESI) detector.

### 2.1. Synthesis

#### 2.1.1. 2–(2,6-Dioxopiperidin-3-yl)-4-fluoroisoindoline-1,3-dione (a2)

4-Fluoroisobenzofuran-1,3-dione (1.0 equiv) was added to a stirred solution of 3-aminopiperidine-2,6-dione (1.1 equiv) and sodium acetate (1.3 equiv) in AcOH. The resulted mixture was heated to 140 °C and stirred for 8 h before being cooled to room temperature. The acetic acid was directly removed under vacuum, and the residue was purified by column chromatography (5% petroleum ether/ethyl acetate) to afford intermediate **a2**. ^1^H NMR (400 MHz, DMSO-d_6_) *δ* 11.13 (s, 1H), 7.97 − 7.92 (m, 1H), 7.79 (d, *J* = 7.3 Hz, 1H), 7.73 (t, *J* = 8.9 Hz, 1H), 5.16 (dd, *J* = 12.8, 5.4 Hz, 1H), 2.89 (ddd, *J* = 17.1, 13.9, 5.5 Hz, 1H), 2.66 − 2.51 (m, 2H), 2.11 − 2.02 (m, 1H). MS (ESI) *m/z* (%): 277.1 [M + H]^+^_._

#### 2.1.2. Tert-Butyl [2–(2,6-dioxopiperidin-3-yl)-1,3-dioxoisoindolin-4-yl]glycinate (a3)

To a solution of intermediate **a2** (1.0 equiv) in DMF, tert-butylglycine (1.2 equiv) and DIPEA (1.3 equiv) were added. The mixtures were heated to 90 °C for 5 h. After being cooled to room temperature, the solution was quenched with water and extracted with CH_2_Cl_2_. The organic layer was dried over Na_2_SO_4_ and filtered. The filtrate was concentrated and purified by column chromatography (20% petroleum ether/ethyl acetate) to obtain intermediate **a3**. ^1^H NMR (400 MHz, CDCl_3_) δ 8.04 (s, 1H), 7.51 (dd, *J* = 8.4, 7.2 Hz, 1H), 7.15 (d, *J* = 6.8 Hz, 1H), 6.76 (d, *J* = 8.4 Hz, 1H), 6.71 (s, 1H), 4.96 − 4.90 (m, 1H), 3.94 (s, 2H), 2.89 − 2.74 (m, 3H), 2.14 − 2.10 (m, 1H), 1.50 (s, 9H). MS (ESI) *m/z* (%): 410.2 [M + Na]^+^.

#### 2.1.3. Synthesis of intermediates a4 and a6

TFA (2 ml) was added to a solution of intermediates **a3** and **a5** in CH_2_Cl_2_ (10 mL), respectively, the reaction was stirred at 25 °C for 8 h. The solvent was evaporated to dryness to obtain intermediates **a4** and **a6**, respectively.

#### 2.1.4. Synthesis of intermediates a7–a11

To a solution of intermediate **a6** (1.0 equiv) in CH_2_Cl_2_, bromine substituted carboxylic acid chain (1.1 equiv), HATU (1.2 equiv) and DIPEA (1.5 equiv) were added. The mixtures were stirred at 25 °C. After the reaction was completed, the solution was diluted with CH_2_Cl_2_, washed with H_2_O and brine, and dried over Na_2_SO_4_ overnight. The organic phase was concentrated under vacuum and purified through column chromatography (20% petroleum ether/ethyl acetate) to obtain intermediates **a7**–**a11**.

#### 2.1.5. Synthesis of intermediates a13–a14

TEA (1.3 equiv) and p-toluenesulfonyl chloride (1.1 equiv) were added to a solution of intermediate **a11**–**a12** (1.0 equiv) in CH_2_Cl_2_. The mixtures were stirred at 30 °C until completion of the reaction. The mixture was diluted with CH_2_Cl_2_, the organic layer was washed with brine, dried over Na_2_SO_4_ and filtered. The filtrate was concentrated and purified by column chromatography (20% petroleum ether/ethyl acetate) to give intermediates **a13**–**a14**.

#### 2.1.6. *Synthesis of intermediates a16*–*a21*

SOCl_2_ (5 mL) was added to the corresponding bromocarboxylic acid chain (1.2 equiv), and the mixture was stirred at 80 °C for 5 h. After being cooled to room temperature, the solvent was evaporated to dryness. The residue was dissolved with dry THF, to the solution was added with commercially available **a15** (1.0 equiv). The mixture was stirred at 50 °C until completion of the reaction. After cooling to room temperature, the crude product of intermediates **a16–a21** can be obtained by filtered.

#### 2.1.7. 2-[2–(2-Azidoethoxy)ethoxy]ethan-1-ol (a23)

2-[2–(2-Chloroethoxy)ethoxy]ethan-1-ol (1.0 equiv) and sodium azide (2.0 equiv) were added to DMF and reacted at 100 °C 12 h. After cooling to room temperature, the reaction was quenched with H_2_O and extracted with CH_2_Cl_2_. The organic layer was washed with brine, dried over Na_2_SO_4_ and filtered. The organic phase was concentrated under vacuum and purified by column chromatography (1% Methanol/Dichloromethane) to obtain intermediate **a23**. MS (ESI) *m/z* (%): 176.3 [M + H]^+^.

#### 2.1.8. 1-Azido-2-[2–(2-bromoethoxy)ethoxy]ethane (a24)

Intermediate **a23** (1.0 equiv), carbon tetrabromide (1.2 equiv), triphenylphosphine (1.2 equiv) were added to CH_2_Cl_2_, and reacted at 25 °C for 12 h. The reaction was quenched with H_2_O and extracted with CH_2_Cl_2_. The organic layer was washed with brine, dried over Na_2_SO_4_ and filtered. The organic phase was concentrated under vacuum and purified by column chromatography (15% petroleum ether/ethyl acetate) to obtain intermediate **a24**. MS (ESI) *m/z* (%): 238.1 [M + H]^+^.

#### 2.1.9. Tert-Butyl 3–(3-hydroxypropoxy)propanoate (a26)

tert-Butyl acrylate (1.0 equiv), 1,3-propanediol (5.0 equiv) and 40% benzyltrimethylammonium hydroxide (0.1 equiv) were added to acetonitrile, and the mixtures were heated to 25 °C and stirred for 3 d. The reaction was quenched with H_2_O and extracted with CH_2_Cl_2_. The organic layer was washed with brine, dried over Na_2_SO_4_ and concentrated under vacuum. The residue was purified by column chromatography (1% Methanol/Dichloromethane) to obtain intermediate **a26**. MS (ESI) *m/z* (%): 227.0 [M + Na]^+^.

#### 2.1.10. Tert-Butyl 3–(3-iodopropoxy)propanoate (a27)

Triphenylphosphine (1.2 equiv), imidazole (1.2 equiv) and iodine (1.5 equiv) were dissolved in dry THF. Intermediate **a26** (1.0 equiv) in THF was added dropwise to the reaction system, and the reaction was stirred at 25 °C for 3 h under Argon. The mixture was filtered to remove the white precipitate. The filtrate was concentrated under vacuum and purified by column chromatography (15% petroleum ether/ethyl acetate) to obtain intermediate **a27**.

#### 2.1.11. N-[2-(aminomethyl)phenyl]-N-methylmethanesulfonamide (a30)

2-Fluoro-benzonitrile (1.0 equiv), *N*-methyl-methanesulfonamide (1.0 equiv) and caesium carbonate (1.2 equiv) were added to acetonitrile and the mixture was stirred at 80 °C for 16 h. After cooling to room temperature, the mixture was filtered. The filtrate was concentrated to obtain the crude product of intermediate **a29**. A 2 M solution of BH_3_ in THF (1.5 equiv) was added to a solution of intermediate **a29** (1.0 equiv) in THF. The reaction was carried out at 60 °C for 12 h under Argon. And H_2_O was added to quench the reaction. The mixture was extracted with CH_2_Cl_2_, the organic layer was washed with brine and dried over Na_2_SO_4_. The organic phase was concentrated and purified by column chromatography (1% Methanol/Dichloromethane) to obtain intermediate **a30**.

#### 2.1.12. Tert-butyl 4–(4-{[4-chloro-5-(trifluoromethyl)pyrimidin-2-yl]amino}phenyl)piperazine-1-carboxylate (a32)

2,4-Dichloro-5-(trifluoromethyl)pyrimidine (1.0 equiv) was added to a mixed solvent of tert-butanol and 1,2-dichloroethane, and zinc bromide (1.2 equiv) was added after cooling to 0 °C. The mixture was stirred for 30 min. Then, tert-butyl 4–(4-aminophenyl)piperazine-1-carboxylate (1.0 equiv) and TEA (2.0 equiv) were added, stirring at 0 °C for 3 h. The reaction was quenched with H_2_O and extracted with CH_2_Cl_2_. The organic layer was washed with brine and dried over Na_2_SO_4_. The organic layer was concentrated under reduced pressure and purified by column chromatography (20% petroleum ether/ethyl acetate) to give intermediate **a32**. ^1^H NMR (400 MHz, CDCl_3_) *δ* 8.51 (s, 1H), 7.54 (s, 1H), 7.44 (d, *J* = 8.8 Hz, 2H), 6.94 (d, *J* = 8.7 Hz, 2H), 3.64 − 3.55 (m, 4H), 3.16 − 3.08 (m, 4H), 1.49 (s, 10H).

#### 2.1.13. Tert-butyl 4-{4-[(4-{[2-(N-methylmethylsulfonamido)benzyl]amino}-5-(trifluoromethyl)pyrimidin-2-yl)amino]phenyl}piperazine-1-carboxylate (a33)

Intermediate **a32** (1.0 equiv), intermediate **a30** (1.0 equiv) and DIPEA (1.0 equiv) were added to 1,4-dioxane, the mixture was heated to 100 °C for 6 h. After cooling to room temperature, the reaction was quenched with H_2_O, extracted with EtOAc, the organic layer was washed with brine and dried over Na_2_SO_4_. The organic phase was concentrated under reduced pressure and purified by column chromatography (1% Methanol/Dichloromethane) to obtain intermediate **a33**. TFA (2 mL) was added to a solution of intermediate **a33** in CH_2_Cl_2_ (10 mL), the mixture was stirred at 25 °C for 5 h. The solvent was removed under reduced pressure, the residue was dissolved with saturated aqueous NaHCO_3_ and extracted with CH_2_Cl_2_. The organic layer was concentrated under reduced pressure to give compound **1**.

#### 2.1.14. N-Methyl-N-(2-{[(2-{[4-(piperazin-1-yl)phenyl]amino}-5-(trifluoromethyl)pyrimidin-4-yl)amino]methyl}phenyl)methanesulfonamide (1)

TFA (2 mL) was added to a solution of intermediate **a33** in CH_2_Cl_2_ (10 mL), the mixture was stirred at 25 °C for 5 h. The solvent was removed under reduced pressure, the residue was dissolved with saturated aqueous NaHCO_3_ and extracted with CH_2_Cl_2_. The organic layer was concentrated under reduced pressure to give compound **1**.

#### 2.1.15. N-(2-{[(2-{[4–(4-{2-[2–(2-azidoethoxy)ethoxy]ethyl}piperazin-1-yl)phenyl]amino}-5-(trifluoromethyl)pyrimidin-4-yl)amino]methyl}phenyl)-N-methylmethanesulfonamide (a34)

Compound **1** (1.0 equiv) and intermediate **a24** (0.12 equiv) were dissolved in acetonitrile, potassium carbonate (1.6 equiv) was added, and the mixture was heated to 80 °C and stirred for 6 h. After cooling to room temperature, H_2_O was added to the solution and extracted with CH_2_Cl_2_. The organic phase was washed with brine, dried over Na_2_SO_4_ overnight. The organic layer was concentrated under reduced pressure and purified by column chromatography (1% Methanol/Dichloromethane) to acquire intermediate **a34**. ^1^H NMR (400 MHz, CDCl_3_) δ 8.13 (s, 1H), 7.54 − 7.44 (m, 2H), 7.40 (d, *J* = 9.0 Hz, 2H), 7.37 − 7.30 (m, 2H), 7.28 − 7.26 (m, 1H), 6.87 (d, *J* = 9.0 Hz, 2H), 5.98 (s, 1H), 5.13 (s, 1H), 4.65 (s, 1H), 3.72 − 3.64 (m, 8H), 3.40 − 3.36 (m, 2H), 3.24 (s, 3H), 3.20 − 3.16 (m, 4H), 2.97 (s, 3H), 2.77 − 2.69 (m, 6H).

#### 2.1.16. N-(2-{[(2-{[4–(4-{2-[2–(2-aminoethoxy)ethoxy]ethyl}piperazin-1-yl)phenyl]amino}-5-(trifluoromethyl)pyrimidin-4-yl)amino]methyl}phenyl)-N-methylmethanesulfonamide (a35)

Intermediate **a34** (1.0 equiv) and triphenylphosphine (3.0 equiv) were added to a mixed solvent of THF (10 ml) and H_2_O (3 mL), and the mixture was stirred at 80 °C until completion of the reaction. The solution was concentrated under reduced pressure and extracted with CH_2_Cl_2_. The organic layer was washed with brine, dried over Na_2_SO_4_ and filtered. The filtrate was concentrated under reduced pressure to give intermediate **a35**.

#### 2.1.17. Synthesis of compounds A1-A2

Intermediate **a4** (1.1 equiv), HATU (1.3 equiv) and DIPEA (1.5 equiv) were added to a solution of intermediate **a35** (1.0 equiv) in CH_2_Cl_2_, the mixture was stirred at 25 °C until completion of the reaction. The solution was diluted with H_2_O and extracted with CH_2_Cl_2_, the organic layer with brine, dried over Na_2_SO_4_ and filtered. The organic layer was concentrated under reduced pressure and purified by column chromatography (6% Methanol/Dichloromethane) to obtain compound **A1**.

Intermediate **a6** (1.1 equiv), HATU (1.3 equiv) and DIPEA (1.5 equiv) were added to a solution of intermediate **a37** (1.0 equiv) in CH_2_Cl_2_, the mixture was stirred at 25 °C until completion of the reaction. The solution was diluted with H_2_O and extracted with CH_2_Cl_2_, the organic layer with brine, dried over Na_2_SO_4_ and filtered. The organic layer was concentrated under reduced pressure and purified by column chromatography (6% Methanol/Dichloromethane) to obtain compound **A2**.

##### 2.1.17.1. 2-{[2–(2,6-Dioxopiperidin-3-yl)-1,3-dioxoisoindolin-4-yl]amino}-N-(2-{2-[2–(4-{4-[(4-{[2-(N-methylmethylsulfonamido)benzyl]amino}-5-(trifluoromethyl)pyrimidin-2-yl)amino]phenyl}piperazin-1-yl)ethoxy]ethoxy}ethyl)acetamide (A1)

Yellow solid; yield: 36.8%; ^1^H NMR (600 MHz, CDCl_3_) δ 10.00 (s, 1H), 8.14 (s, 1H), 7.51 − 7.48 (m, 1H), 7.44 (d, *J* = 5.8 Hz, 1H), 7.39 (d, *J* = 8.7 Hz, 2H), 7.35 − 7.27 (m, 3H), 7.18 (d, *J* = 7.1 Hz, 1H), 6.97 (s, 1H), 6.84 (d, *J* = 8.7 Hz, 2H), 6.80 (d, *J* = 8.5 Hz, 1H), 6.70 (t, *J* = 5.7 Hz, 1H), 5.96 (s, 1H), 5.13 (s, 1H), 4.90 (dd, *J* = 11.4, 4.4 Hz, 1H), 4.66 (s, 1H), 3.94 (d, *J* = 5.8 Hz, 2H), 3.62 − 3.52 (m, 9H), 3.45 − 3.42 (m, 1H), 3.24 (s, 3H), 3.15 (s, 4H), 2.97 (s, 4H), 2.86 − 2.77 (m, 2H), 2.72 − 2.64 (m, 6H), 2.11 (dd, *J* = 8.8, 3.9 Hz, 1H). ^13 ^C NMR (150 MHz, DMSO-d_6_) δ 172.81, 170.05, 168.70, 168.58, 167.31, 160.93, 158.34, 154.70, 146.25, 145.80, 139.57, 139.23, 136.22, 132.05, 131.79, 128.37, 127.83, 127.56, 127.07, 125.20 (q, *J* = 269.2 Hz), 120.75 (2 C), 117.46, 115.68 (2 C), 110.98, 109.86, 69.62, 69.52, 68.94, 67.98, 57.03, 52.99 (2 C), 48.57 (3 C), 45.17, 38.66, 38.57, 36.00, 30.98, 22.16. HRMS calcd for C_45_H_52_F_3_N_11_O_9_S, [M + H]^+^, 980.3695; found 980.3712.

##### 2.1.17.2. N-(2-{[2–(2,6-dioxopiperidin-3-yl)-1,3-dioxoisoindolin-4-yl]amino}ethyl)-3-[3–(4-{4-[(4-{[2-(N-methylmethylsulfonamido)benzyl]amino}-5-(trifluoromethyl)pyrimidin-2-yl)amino]phenyl}piperazin-1-yl)propoxy]propanamide (A2)

Yellow solid; yield: 31.7%; ^1^H NMR (600 MHz, CDCl_3_) δ 10.76 (s, 1H), 8.14 (s, 1H), 7.72 (s, 1H), 7.50 − 7.46 (m, 1H), 7.45 − 7.41 (m, 3H), 7.34 − 7.27 (m, 3H), 7.08 (d, *J* = 7.1 Hz, 1H), 6.97 (d, *J* = 8.6 Hz, 1H), 6.82 (d, *J* = 8.7 Hz, 3H), 6.41 (t, *J* = 4.9 Hz, 1H), 5.99 (s, 1H), 5.12 (s, 1H), 4.88 (dd, *J* = 12.0, 5.4 Hz, 1H), 4.65 (d, *J* = 11.3 Hz, 1H), 3.66 (t, *J* = 5.6 Hz, 2H), 3.51 − 3.44 (m, 6H), 3.22 (s, 3H), 3.15 (s, 4H), 2.96 (s, 3H), 2.80 − 2.65 (m, 7H), 2.50 − 2.46 (m, 4H), 2.09 (dd, *J* = 7.8, 5.3 Hz, 1H), 1.82 − 1.78 (m, 2H). ^13 ^C NMR (150 MHz, CDCl_3_) δ 172.53, 172.47, 169.60, 169.56, 167.70, 161.05, 158.88, 154.69, 147.17, 146.82, 139.93, 139.11, 136.36, 132.60, 132.01, 130.67, 129.32, 128.87, 126.42, 125.00 (q, *J* = 269.3 Hz), 121.89 (2 C), 116.92 (2 C), 116.88, 111.94, 110.40, 69.42, 66.88, 55.33, 52.95 (2 C), 49.23 (2 C), 49.00, 42.17, 40.65, 39.32, 39.02, 37.11, 35.63, 31.56, 26.37, 22.98. HRMS calcd for C_45_H_52_F_3_N_11_O_8_S, [M + H]^+^, 964.3746; found 964.3788.

#### 2.1.18. Synthesis of compounds A3-A15

Potassium iodide (1.1 equiv) and potassium carbonate (1.3 equiv) were added to a stirred solution of compound **1** (1.0 equiv) and the corresponding int ermediates **a13–a14**, **a16–a21** and **a7–a11** (1.1 equiv) in acetonitrile. The mixture was stirred at 80 °C until completion of the reaction. The solution was diluted with H_2_O and extracted with CH_2_Cl_2_. The organic layer was washed with brine, dried over Na_2_SO_4_ and filtered. The filtrate was concentrated under reduced pressure and purified by column chromatography (6% Methanol/Dichloromethane) to obtain compounds **A3–A4**, **A5–A10** and **A11–A15**.

##### 2.1.18.1. N-(2-{[(2-{[4–(4-{2-[2–(2-{[2–(2,6-Dioxopiperidin-3-yl)]-1,3- Dioxyisoindolin-4-acyl}amino)ethoxy]ethoxy}ethylpiperazin-1-yl)phenyl]amino}-5-(trifluoromethyl)pyrimidin-4-yl)amino]methyl}phenyl)-N-methylmethanesulfonamide (A3)

Yellow solid; yield: 37.7%; ^1^H NMR (600 MHz, CDCl_3_) δ 10.40 (s, 1H), 8.15 (s, 1H), 7.71 (s, 1H), 7.49 − 7.45 (m, 1H), 7.44 (d, *J* = 6.6 Hz, 1H), 7.40 (d, *J* = 9.0 Hz, 2H), 7.34 − 7.27 (m, 3H), 7.09 (d, *J* = 7.1 Hz, 1H), 6.90 (d, *J* = 8.6 Hz, 1H), 6.85 (d, *J* = 8.9 Hz, 2H), 6.52 (t, *J* = 5.5 Hz, 1H), 6.00 (s, 1H), 5.13 (s, 1H), 4.87 (dd, *J* = 11.5, 4.1 Hz, 1H), 4.64 (d, *J* = 11.5 Hz, 1H), 3.73 − 3.69 (m, 4H), 3.67 − 3.64 (m, 4H), 3.47 − 3.44 (m, 2H), 3.22 (s, 3H), 3.17 (s, 4H), 2.96 (s, 3H), 2.80 − 2.65 (m, 9H), 2.10 − 2.06 (m, 1H). ^13 ^C NMR (150 MHz, CDCl_3_) δ 172.28, 169.44, 169.30, 167.75, 161.07, 158.89, 154.69, 147.33, 146.89, 139.91, 139.14, 136.14, 132.66, 132.01, 130.70, 129.31, 128.84, 126.41, 125.00 (q, *J* = 269.3 Hz), 121.98 (2 C), 116.99 (2 C), 116.83, 111.75, 110.48, 70.78, 70.48, 69.51, 68.77, 57.70, 53.45 (2 C), 49.54 (2 C), 48.96, 42.48, 40.67, 39.30, 35.64, 31.50, 23.00. HRMS calcd for C_43_H_49_F_3_N_10_O_8_S, [M + H]^+^, 923.3480; found 923.3513.

##### 2.1.18.2. N-{2-[({[2-({4-[4–(2-{2-[2–(2-{[2–(2,6-Dioxopiperidin-3-yl)]- 1,3-Dioxyisoindoline-4-acyl}amino)ethoxy]ethoxy}ethoxy)ethylpiperazin-1-yl]phenyl}amino)-5-(trifluoromethyl) yl]pyrimidin-4-yl}amino)methyl]phenyl}-N-methylmethanesulfonamide (A4)

Yellow solid; yield: 29.6%; ^1^H NMR (600 MHz, CDCl_3_) δ 10.79 (s, 1H), 8.15 (s, 1H), 7.61 (s, 1H), 7.49 − 7.43 (m, 2H), 7.39 (d, *J* = 8.9 Hz, 2H), 7.35 − 7.27 (m, 3H), 7.09 (d, *J* = 7.1 Hz, 1H), 6.89 (d, *J* = 8.5 Hz, 1H), 6.83 (d, *J* = 8.9 Hz, 2H), 6.50 (t, *J* = 5.4 Hz, 1H), 5.99 (s, 1H), 5.13 (s, 1H), 4.88 (dd, *J* = 12.3, 5.4 Hz, 1H), 4.64 (d, *J* = 11.8 Hz, 1H), 3.72 − 3.64 (m, 12H), 3.46 − 3.42 (m, 2H), 3.23 (s, 3H), 3.16 (s, 4H), 2.96 (s, 3H), 2.84 − 2.67 (m, 9H), 2.12 − 2.08 (m, 1H). ^13 ^C NMR (150 MHz, CDCl_3_) δ 172.27, 169.46, 169.44, 167.80, 161.13, 158.90, 154.79, 147.45, 146.88, 139.93, 139.17, 136.12, 132.69, 131.82, 130.71, 129.32, 128.85, 126.42, 125.02 (q, *J* = 269.7 Hz), 121.99 (2 C), 116.85 (2 C), 116.81, 111.73, 110.49, 70.96, 70.60 (2 C), 70.43, 69.44, 68.48, 57.53, 53.44 (2 C), 49.40 (2 C), 49.02, 42.43, 40.65, 39.32, 35.66, 31.64, 23.05. HRMS calcd for C_45_H_53_F_3_N_10_O_9_S, [M + H]^+^, 967.3743; found 967.3778.

##### 2.1.18.3. N-[2–(2,6-Dioxopiperidin-3-yl)1,3-dioxoisoindol-4-yl]-5–(4-{4-[(4-{[2 -(N-Methylmethylsulfonamido)benzyl]amino)}5-(trifluoromethyl)pyrimidin-2-yl)amino]phenyl}piperazin-1-yl)pentanamide (A5)

Yellow solid; yield: 32.5%; ^1^H NMR (600 MHz, CDCl_3_) δ 10.04 (s, 1H), 9.43 (s, 1H), 8.81 (d, *J* = 8.5 Hz, 1H), 8.15 (s, 1H), 7.83 − 7.68 (m, 2H), 7.54 (d, *J* = 7.3 Hz, 1H), 7.44 (d, *J* = 7.3 Hz, 1H), 7.41 (d, *J* = 8.8 Hz, 2H), 7.35 − 7.27 (m, 3H), 6.85 (d, *J* = 8.8 Hz, 2H), 6.00 (s, 1H), 5.14 (s, 1H), 4.92 (dd, *J* = 12.4, 5.3 Hz, 1H), 4.64 (d, *J* = 12.3 Hz, 1H), 3.23 (s, 3H), 3.14 (s, 4H), 2.96 (s, 3H), 2.87 (d, *J* = 15.8 Hz, 1H), 2.79 − 2.71 (m, 2H), 2.61 (s, 4H), 2.50 (t, *J* = 7.3 Hz, 2H), 2.47 − 2.44 (m, 2H), 2.13 − 2.10 (m, 1H), 1.82 − 1.77 (m, 2H), 1.66 − 1.60 (m, 2H). ^13 ^C NMR (150 MHz, CDCl_3_) δ 172.25, 171.83, 169.34, 168.67, 166.84, 161.06, 158.91, 154.67, 147.57, 139.94, 139.17, 137.92, 136.55, 131.79, 131.25, 130.77, 129.33, 128.87, 126.40, 125.37, 125.00 (q, *J* = 269.4 Hz), 121.95 (2 C), 118.59, 116.82 (2 C), 115.45, 58.01, 53.23 (2 C), 49.70 (2 C), 49.39, 40.68, 39.34, 37.78, 35.65, 31.51, 26.10, 23.30, 22.82. HRMS calcd for C_42_H_45_F_3_N_10_O_7_S, [M + H]^+^, 891.3218; found 891.3225.

##### 2.1.18.4. N-(2–(2,6-Dioxopiperidin-3-yl)-1,3-dioxoisoindol-4-yl)-6–(4-{4-[(4- {[2-(N-Methylmethylsulfonamido)benzyl]amino}-5-(trifluoromethyl)pyrimidin-2-yl)amino]phenyl}piperazin-1-yl)hexanamide (A6)

Yellow solid; yield: 41.9%; ^1^H NMR (600 MHz, CDCl_3_) δ 9.90 (s, 1H), 9.43 (s, 1H), 8.81 (d, *J* = 8.5 Hz, 1H), 8.15 (s, 1H), 7.78 − 7.64 (m, 2H), 7.54 (d, *J* = 7.2 Hz, 1H), 7.45 − 7.41 (m, 3H), 7.36 − 7.27 (m, 3H), 6.86 (d, *J* = 8.8 Hz, 2H), 6.01 (s, 1H), 5.13 (s, 1H), 4.94 (dd, *J* = 12.3, 5.3 Hz, 1H), 4.65 (d, *J* = 11.6 Hz, 1H), 3.24 (s, 3H), 3.21 (s, 4H), 2.97 (s, 3H), 2.89 (d, *J* = 15.7 Hz, 1H), 2.81 − 2.70 (m, 6H), 2.53 − 2.46 (m, 4H), 2.1 − 2.14 (m, 1H), 1.82 − 1.76 (m, 2H), 1.67 − 1.61 (m, 2H), 1.46 − 1.41 (m, 2H). ^13 ^C NMR (150 MHz, CDCl_3_) δ 172.24, 171.69, 169.35, 168.60, 166.83, 161.02, 158.91, 154.64, 147.22, 139.95, 139.15, 137.91, 136.57, 132.11, 131.25, 130.74, 129.35, 128.89, 126.41, 125.38, 124.98 (q, *J* = 269.6 Hz), 121.94 (2 C), 118.61, 117.12 (2 C), 115.48, 58.19, 53.02 (2 C), 49.40 (2 C), 49.37, 40.69, 39.35, 37.87, 35.65, 31.51, 26.98, 25.90, 25.11, 22.88. HRMS calcd for C_43_H_47_F_3_N_10_O_7_S, [M + H]^+^, 905.3375; found 905.3394.

##### 2.1.18.5. N-[2–(2,6-Dioxopiperidin-3-yl)-1,3-dioxoisoindol-4-yl]-7–(4-{4-[(4-{[2-(N-Methylmethylsulfonamido)benzyl]amino}-5-(trifluoromethyl)pyrimidin-2-yl)amino]phenyl}piperazin-1-yl)heptamide (A7)

Yellow solid; yield: 36.1%; ^1^H NMR (600 MHz, CDCl_3_) δ 10.10 (s, 1H), 9.44 (s, 1H), 8.80 (d, *J* = 8.5 Hz, 1H), 8.14 (s, 1H), 7.70 (t, *J* = 7.9 Hz, 1H), 7.54 (d, *J* = 7.3 Hz, 1H), 7.44 − 7.40 (m, 3H), 7.35 − 7.27 (m, 3H), 6.87 (d, *J* = 8.7 Hz, 2H), 6.00 (s, 1H), 5.13 (s, 1H), 4.93 (s, 1H), 4.65 (d, *J* = 11.3 Hz, 1H), 3.23 (s, 3H), 3.18 (s, 4H), 2.97 (s, 3H), 2.88 (d, *J* = 15.6 Hz, 1H), 2.79 − 2.72 (m, 2H), 2.68 (s, 4H), 2.46 (t, *J* = 7.1 Hz, 4H), 2.17 − 2.14 (m, 1H), 1.79 − 1.74 (m, 2H), 1.60 − 1.57 (m, 2H), 1.44 − 1.38 (m, 4H). ^13 ^C NMR (150 MHz, CDCl_3_) δ 172.41, 171.72, 169.41, 168.70, 166.84, 161.12, 158.90, 154.77, 147.40, 139.95, 139.17, 137.93, 136.56, 131.97, 131.27, 130.76, 129.34, 128.87, 126.42, 125.40, 125.00 (q, *J* = 269.4 Hz), 122.04 (2 C), 118.59, 117.04 (2 C), 115.48, 58.50, 53.04 (2 C), 49.55 (2 C), 49.37, 40.68, 39.34, 38.01, 35.66, 31.48, 28.99, 27.23, 26.12, 25.28, 22.92. HRMS calcd for C_44_H_49_F_3_N_10_O_7_S, [M + H]^+^, 919.3531; found 919.3543.

##### 2.1.18.6. N-[2–(2,6-Dioxopiperidin-3-yl)-1,3-dioxoisoindol-4-yl]-8–(4-{4-[(4-{[2-(N-Methylmethylsulfonamido)benzyl]amino}-5-(trifluoromethyl)pyrimidin-2-yl)amino]phenyl}piperazin-1-yl)octamide (A8)

Yellow solid; yield: 38.2%; ^1^H NMR (600 MHz, CDCl_3_) δ 10.73 (s, 1H), 9.44 (s, 1H), 8.79 (d, *J* = 8.5 Hz, 1H), 8.13 (s, 1H), 7.98 (s, 1H), 7.69 (t, *J* = 7.9 Hz, 1H), 7.53 (d, *J* = 7.3 Hz, 1H), 7.45 − 7.41 (m, 3H), 7.35 − 7.27 (m, 3H), 6.84 (d, *J* = 8.8 Hz, 2H), 6.04 (s, 1H), 5.12 (s, 1H), 4.93 (dd, *J* = 11.9, 5.4 Hz, 1H), 4.64 (d, *J* = 11.2 Hz, 1H), 3.28 − 3.20 (m, 7H), 2.96 (s, 3H), 2.87 (d, *J* = 13.6 Hz, 1H), 2.76 − 2.72 (m, 6H), 2.53 − 2.49 (m, 2H), 2.44 (t, *J* = 7.3 Hz, 2H), 2.17 − 2.15 (m, 1H), 1.74 (dt, *J* = 13.3, 6.7 Hz, 2H), 1.62 − 1.58 (m, 2H), 1.41 − 1.30 (m, 6H). ^13 ^C NMR (150 MHz, CDCl_3_) δ 172.49, 172.06, 169.40, 168.94, 166.86, 160.88, 158.90, 154.36, 147.09 (2 C), 139.92, 139.07, 137.88, 136.52, 132.16, 131.27, 130.64, 129.33, 128.88, 126.43, 125.36, 124.92 (q, *J* = 269.4 Hz), 121.89 (2 C), 118.55 (2 C), 117.13, 115.49, 58.47, 52.85 (2 C), 49.39, 49.04 (2 C), 40.71, 39.34, 37.90, 35.65, 31.52, 29.14, 28.91, 27.45, 25.87, 25.34, 22.93. HRMS calcd for C_45_H_51_F_3_N_10_O_7_S, [M + H]^+^, 933.3688; found 933.3702.

##### 2.1.18.7. N-[2–(2,6-Dioxopiperidin-3-yl)-1,3-dioxoisoindol-4-yl]-9–(4-{4-[(4-{[2-(N-methylmethylsulfonamido)benzyl]amino}-5-(trifluoromethyl)pyrimidin-2-yl)amino]phenyl}piperazin-1-yl)nonanamide (A9)

Yellow solid; yield: 29.7%; ^1^H NMR (600 MHz, CDCl_3_) δ 10.97 (s, 1H), 9.47 (s, 1H), 8.80 (d, *J* = 8.5 Hz, 1H), 8.14 (s, 1H), 7.77 − 7.64 (m, 2H), 7.53 (d, *J* = 7.3 Hz, 1H), 7.45 − 7.40 (m, 3H), 7.35 − 7.26 (m, 3H), 6.84 (d, *J* = 8.9 Hz, 2H), 6.02 (s, 1H), 5.12 (s, 1H), 4.93 (dd, *J* = 12.2, 5.4 Hz, 1H), 4.64 (d, *J* = 11.6 Hz, 1H), 3.25 − 3.21 (m, 7H), 2.96 (s, 3H), 2.86 (dd, *J* = 13.2, 2.4 Hz, 1H), 2.80 − 2.71 (m, 6H), 2.52 (s, 2H), 2.45 (dd, *J* = 14.3, 7.3 Hz, 2H), 2.17 − 2.12 (m, 1H), 1.77 − 1.71 (m, 2H), 1.63 − 1.58 (m, 2H), 1.40 − 1.30 (m, 8H). ^13 ^C NMR (150 MHz, CDCl_3_) δ 172.51, 172.17, 169.36, 168.99, 166.88, 161.02, 158.89, 154.65, 147.06, 139.91, 139.10, 137.91, 136.49, 132.15, 131.25, 130.61, 129.31, 128.85, 126.42, 125.30, 124.97 (q, *J* = 270.5 Hz), 121.94 (2 C), 118.49, 117.12 (2 C), 115.46, 58.39, 52.78 (2 C), 49.37, 49.05 (2 C), 40.67, 39.31, 38.04, 35.64, 31.55, 29.17, 29.06, 28.91, 27.38, 25.84, 25.24, 22.89. HRMS calcd for C_46_H_53_F_3_N_10_O_7_S, [M + H]^+^, 947.3844; found 947.3866.

##### 2.1.18.8. N-[2–(2,6-Dioxopiperidin-3-yl)-1,3-dioxoisoindol-4-yl]-10–(4-{4-[(4-{[2-(N-methylmethylsulfonamido)benzyl]amino}-5-(trifluoromethyl)pyrimidin-2-yl)amino]phenyl}piperazin-1-yl)decanoamide (A10)

Yellow solid; yield: 39.1%; ^1^H NMR (600 MHz, CDCl_3_) δ 11.02 (s, 1H), 9.44 (s, 1H), 8.81 (d, *J* = 8.3 Hz, 1H), 8.14 (s, 1H), 7.76 (s, 1H), 7.69 (t, *J* = 7.7 Hz, 1H), 7.53 (d, *J* = 7.0 Hz, 1H), 7.44 − 7.39 (m, 3H), 7.34 − 7.27 (m, 3H), 6.84 (d, *J* = 8.1 Hz, 2H), 6.01 (s, 1H), 5.13 (s, 1H), 4.93 (dd, *J* = 11.8, 4.8 Hz, 1H), 4.64 (d, *J* = 8.0 Hz, 1H), 3.23 (s, 3H), 3.18 (s, 4H), 2.96 (s, 3H), 2.87 (d, *J* = 15.6 Hz, 1H), 2.81 − 2.69 (m, 6H), 2.46 − 2.43 (m, 4H), 2.16 − 2.13 (m, 1H), 1.76 − 1.72 (m, 2H), 1.55 (s, 2H), 1.40 − 1.30 (m, 10H). ^13 ^C NMR (150 MHz, CDCl_3_) δ 172.58, 172.18, 169.35, 168.92, 166.90, 161.03, 158.90, 154.61, 147.34 (2 C), 139.92, 139.12, 137.93, 136.48, 131.89, 131.24, 130.69, 129.32, 128.85, 126.42, 125.32, 124.97 (q, *J* = 269.5 Hz), 121.94 (2 C), 118.48, 116.90 (2 C), 115.45, 58.65, 52.97 (2 C), 49.41, 49.30 (2 C), 40.67, 39.32, 38.15, 35.65, 31.61, 29.55, 29.21, 29.17, 29.02, 27.61, 26.21, 25.33, 22.86. HRMS calcd for C^47^H^55^F^3^N^10^O^7^S, [M + H]^+^, 961.4001; found 961.4022.

##### 2.1.18.9. N-(2-{[2–(2,6-Dioxopiperidin-3-yl)-1,3-dioxoisoindolin-4-yl]amino}ethyl)-5–(4 –{4-[(4-{[2-(N-Methylmethylsulfonamido)benzyl]amino}-5-(trifluoromethyl)pyrimidin-2-yl)amino]phenyl}piperazine -1-yl)pentanamide (A11)

Yellow solid; yield: 40.3%; ^1^H NMR (600 MHz, CDCl_3_) δ 10.57 (s, 1H), 8.14 (s, 1H), 7.78 (s, 1H), 7.46 (dd, *J* = 17.1, 9.0 Hz, 2H), 7.41 (d, *J* = 8.6 Hz, 2H), 7.34 − 7.26 (m, 3H), 7.07 (d, *J* = 7.1 Hz, 1H), 6.96 (d, *J* = 8.6 Hz, 1H), 6.82 (d, *J* = 8.6 Hz, 2H), 6.48 (s, 1H), 6.41 (s, 1H), 6.00 (s, 1H), 5.12 (s, 1H), 4.89 (dd, J = 12.0, 5.3 Hz, 1H), 4.65 (d, *J* = 11.5 Hz, 1H), 3.44 (s, 4H), 3.22 (s, 3H), 3.13 (s, 4H), 2.96 (s, 3H), 2.82 − 2.70 (m, 3H), 2.63 (s, 4H), 2.44 (s, 2H), 2.20 (t, *J* = 7.0 Hz, 2H), 2.09 − 2.05 (m, 1H), 1.66 − 1.62 (m, 2H), 1.58 − 1.54 (m, 2H). ^13 ^C NMR (150 MHz, CDCl_3_) δ 173.89, 172.44, 169.58 (2 C), 167.69, 161.05, 158.88, 154.66, 147.24, 146.86, 139.92, 139.11, 136.36, 132.58, 131.94, 130.67, 129.32, 128.87, 126.42, 125.00 (q, *J* = 269.3 Hz), 121.91 (2 C), 116.93, 116.87 (2 C), 111.96, 110.41, 57.83, 52.98 (2 C), 49.34 (2 C), 49.02, 42.25, 40.66, 39.31, 39.01, 36.10, 35.64, 31.56, 25.82, 23.46, 22.91. HRMS calcd for C_44_H_50_F_3_N_11_O_7_S, [M + H]^+^, 934.3640; found 934.3689.

##### 2.1.18.10. N-(2-{[2–(2,6-Dioxopiperidin-3-yl)-1,3-dioxoisoindolin-4-yl]amino}ethyl)-6–(4-{4-[(4-{[2-(N-methylmethylsulfonamido)benzyl]amino}-5-(trifluoromethyl)pyrimidin-2-yl)amino]phenyl}piperazine-1-yl)hexanamide (A12)

Yellow solid; yield: 32.9%; ^1^H NMR (600 MHz, CDCl_3)_ δ 11.03 (s, 1H), 8.14 (s, 1H), 7.65 (s, 1H), 7.47 (t, *J* = 7.7 Hz, 1H), 7.43 (d, *J* = 6.9 Hz, 1H), 7.39 (d, *J* = 8.3 Hz, 2H), 7.35 − 7.27 (m, 3H), 7.09 (d, *J* = 7.0 Hz, 1H), 6.95 (d, J = 8.4 Hz, 1H), 6.82 (d, *J* = 8.4 Hz, 2H), 6.40 (s, 1H), 6.24 (s, 1H), 5.98 (s, 1H), 5.12 (s, 1H), 4.88 (dd, *J* = 11.6, 5.1 Hz, 1H), 4.68 − 4.59 (m, 1H), 3.53 − 3.41 (m, 4H), 3.22 (s, 3H), 3.16 (s, 4H), 2.96 (s, 3H), 2.84 − 2.66 (m, 7H), 2.43 (d, *J* = 6.3 Hz, 2H), 2.18 (s, 2H), 2.11 − 2.07 (m, 1H), 1.68–1.63 (m, 2H), 1.58 − 1.54 (m, 2H), 1.35 − 1.31 (m, 2H). ^13 ^C NMR (150 MHz, CDCl_3_) δ 174.04, 172.46, 169.74, 169.60, 167.70, 161.13, 158.90, 154.78, 147.30, 146.81, 139.95, 139.14, 136.33, 132.66, 131.95, 130.66, 129.33, 128.87, 126.45, 125.03 (q, *J* = 269.5 Hz), 121.96 (2 C), 116.93 (3 C), 112.01, 110.60, 58.07, 52.89 (2 C), 49.25 (2 C), 49.05, 42.01, 40.68, 39.32, 38.82, 36.49, 35.69, 31.61, 26.97, 25.74, 25.67, 22.99. HRMS calcd for C_45_H_52_F_3_N_11_O_7_S, [M + H]^+^, 948.3797; found 948.3851.

##### 2.1.18.11. N-(2-{[2–(2,6-Dioxopiperidin-3-yl)-1,3-dioxoisoindolin-4-yl]amino}ethyl)-7–(4 -{4-[(4-{[2-(N-methylmethylsulfonamido)benzyl]amino}-5-(trifluoromethyl)pyrimidin-2-yl)amino]phenyl}piperazine -1-yl)heptamide (A13)

Yellow solid; yield: 31.4%; ^1^H NMR (600 MHz, CDCl_3_) δ 11.07 (s, 1H), 8.14 (s, 1H), 7.60 (s, 1H), 7.49 − 7.46 (m, 1H), 7.44 (d, *J* = 7.1 Hz, 1H), 7.39 (d, *J* = 8.6 Hz, 2H), 7.35 − 7.27 (m, 3H), 7.09 (d, *J* = 7.1 Hz, 1H), 6.93 (d, *J* = 8.5 Hz, 1H), 6.83 (d, *J* = 8.7 Hz, 2H), 6.41 (s, 1H), 6.11 (s, 1H), 5.98 (s, 1H), 5.12 (s, 1H), 4.88 (dd, *J* = 12.2, 5.4 Hz, 1H), 4.64 (d, *J* = 9.0 Hz, 1H), 3.59 − 3.56 (m, 1H), 3.47 − 3.40 (m, 3H), 3.22 (s, 3H), 3.17 (s, 4H), 2.96 (s, 3H), 2.83 − 2.67 (m, 7H), 2.49 − 2.40 (m, 2H), 2.22 − 2.13 (m, 2H), 2.09 − 2.07 (m, 1H), 1.65 (t, *J* = 11.2 Hz, 2H), 1.54 (s, 2H), 1.36 − 1.29 (m, 4H). ^13 ^C NMR (150 MHz, CDCl_3_) δ 174.11, 172.45, 169.62, 169.50, 167.73, 161.14, 158.90, 154.79, 147.33, 146.77, 139.95, 139.15, 136.28, 132.66, 131.89, 130.67, 129.33, 128.87, 126.46, 125.03 (q, *J* = 269.6 Hz), 121.98 (2 C), 116.90 (2 C), 116.78, 111.93, 110.57, 58.46, 52.90 (2 C), 49.17 (2 C), 49.05, 42.28, 40.67, 39.33, 38.89, 36.68, 35.70, 31.65, 29.19, 27.36, 25.91, 25.58, 22.97. HRMS calcd for C_46_H_54_F_3_N_11_O_7_S, [M + H]^+^, 962.3953; found 962.4011.

##### 2.1.18.12. N-(2-{[2–(2,6-Dioxopiperidin-3-yl)-1,3-dioxoisoindolin-4-yl]amino}ethyl)-8–(4-{4-[(4-{[2-(N-methylmethylsulfonamido)benzyl]amino}-5-(trifluoromethyl)pyrimidin-2-yl)amino]phenyl}piperazine-1-yl) octanamide (A14)

Yellow solid; yield: 39.7%; ^1^H NMR (600 MHz, CDCl_3_) δ 10.46 (s, 1H), 8.14 (s, 1H), 7.62 (s, 1H), 7.50 − 7.47 (m, 1H), 7.44 (d, *J* = 7.5 Hz, 1H), 7.40 (d, *J* = 8.9 Hz, 2H), 7.35 − 7.26 (m, 3H), 7.09 (d, *J* = 7.1 Hz, 1H), 6.98 (d, *J* = 8.6 Hz, 1H), 6.83 (d, *J* = 8.9 Hz, 2H), 6.40 (d, J = 5.0 Hz, 1H), 6.13 (s, 1H), 5.99 (s, 1H), 5.12 (s, 1H), 4.90 (dd, *J* = 12.3, 5.4 Hz, 1H), 4.64 (d, *J* = 13.1 Hz, 1H), 3.49 − 3.43 (m, 4H), 3.23 (s, 3H), 3.20 (s, 4H), 2.97 (s, 3H), 2.86 − 2.67 (m, 7H), 2.46 (d, *J* = 7.7 Hz, 2H), 2.16 (t, *J* = 7.5 Hz, 2H), 2.11 − 2.08 (m, 1H), 1.63 − 1.59 (m, 2H), 1.56 − 1.53 (m, 2H), 1.33 − 1.28 (m, 6H). ^13 ^C NMR (150 MHz, CDCl_3_) δ 174.12, 172.23, 169.58, 169.45, 167.71, 161.11, 158.90, 154.75, 147.23, 146.84, 139.94, 139.15, 136.37, 132.62, 131.99, 130.69, 129.35, 128.88, 126.43, 125.01 (q, *J* = 269.9 Hz), 121.96 (2 C), 117.00 (2 C), 116.88, 112.01, 110.50, 58.32, 52.91 (2 C), 49.20 (2 C), 49.08, 42.12, 40.67, 39.34, 39.04, 36.62, 35.67, 31.67, 29.03, 28.98, 27.10, 26.04, 25.61, 22.93. HRMS calcd for C_47_H_56_F_3_N_11_O_7_S, [M + H]^+^, 976.4110; found 976.4171.

##### 2.1.18.13. N-(2-{[2–(2,6-Dioxopiperidin-3-yl)-1,3-dioxoisoindolin-4-yl]amino}ethyl)-10–(4 -{4-[(4-{[2-(N-methylmethylsulfonamido)benzyl]amino}-5-(trifluoromethyl)pyrimidin-2-yl)amino]phenyl}piperazine -1-yl)decanoamide (A15)

Yellow solid; yield: 34.8%; ^1^H NMR (600 MHz, CDCl_3_) δ 9.94 (s, 1H), 8.15 (s, 1H), 7.57 − 7.44 (m, 3H), 7.41 (d, *J* = 8.9 Hz, 2H), 7.35 − 7.27 (m, 3H), 7.10 (d, *J* = 7.1 Hz, 1H), 7.00 (d, *J* = 8.6 Hz, 1H), 6.85 (d, *J* = 8.9 Hz, 2H), 6.40 (d, *J* = 5.2 Hz, 1H), 5.99 (d, *J* = 15.2 Hz, 2H), 5.14 (s, 1H), 4.91 (dd, J = 12.4, 5.4 Hz, 1H), 4.66 (s, 1H), 3.49 − 3.44 (m, 4H), 3.24 (s, 3H), 3.19 (s, 4H), 2.97 (s, 3H), 2.87 − 2.66 (m, 7H), 2.44 (s, 2H), 2.16 (t, *J* = 7.5 Hz, 2H), 2.12 − 2.08 (m, 1H), 1.63 − 1.60 (m, 2H), 1.57 − 1.53 (m, 2H), 1.33–1.26 (m, 8H). ^13 ^C NMR (150 MHz, CDCl_3_) δ 174.09, 171.95, 169.59, 169.19, 167.68, 161.19, 158.92, 154.85, 147.43, 146.91, 139.98, 139.20, 136.39, 132.64, 131.89, 130.76, 129.35, 128.88, 126.45, 125.05 (q, *J* = 269.4 Hz), 122.02 (2 C), 116.97 (2 C), 116.88, 112.07, 110.54, 58.60, 53.08 (2 C), 49.40 (2 C), 49.11, 42.22, 40.68, 39.36, 39.19, 36.71, 35.72, 31.67, 29.25 (2 C), 29.19, 27.45, 26.31, 25.70, 22.94. HRMS calcd for C_49_H_60_F_3_N_1_1O_7_S, [M + H]^+^, 1004.4423; found 1004.4482.

### 2.2. Fak HTRF assay

The FAK kinase assay was performed using the HTRF^®^ KinEASE™-TK kit (Cisbio Bioassays, France) in white 384-well small volume plates with a total working volume of 20 μL. Purified FAK enzyme was purchased from Carna Biosciences (Japan). Compounds were diluted step-by-step from a concentrated stock of 8 mM in 100% DMSO and with serial kinase reaction buffer dilutions. The IC_50_ measurements were performed in replicates. For each assay, 4 μL of dispensed compounds, 4 μL of mix 1 (ATP + Substrate TK) and 2 μL of kinase (0.111 ng/mL) were added to the assay wells. The assay plates were incubated at 30 °C for 50 min and reactions were terminated by adding 10 μL of mix 2 (Sa-XL665 + TK-Antibody-Cryptate). After a final incubation (60 min at room temperature), HTRF signals were obtained by reading plates using an Infinite^®^ F500 microplate reader (Tecan, Switzerland). The fluorescence was measured at 620 nM (Cryptate) and 665 nM (XL665). A ratio was calculated (665/620) for each well. For IC_50_ measurements, values were normalised and fitted with Graphpad prism 8.3.0.

### 2.3. Cell proliferation assay

A549 cells were cultured in a 96-well plate at a density of 4000 cells/well and were maintained at 37 °C in a humidified atmosphere of 5% CO_2_ for 24 h. The tested compounds were added to the culture medium at the indicated final concentrations and incubated for 72 h. Fresh MTT was added to each well at a final concentration of 5 mg/mL in phosphate-buffered saline (PBS), and the cells were then incubated at 37 °C for 4 h. Removing culture solution and the formazan crystals in each well were dissolved in 150 μL DMSO, and the absorbance of each test was measured at *λ*_490nm_ using Infinite^®^ F500 microplate reader (Tecan, Switzerland). The IC_50_ values were calculated by concentration-response curve fitting using Graphpad prism 8.3.0.

### 2.4. Western blotting

Antibodies used in this study are: Anti-FAK (CST-3285S) was obtained from Cell Signalling Technology (MA, USA), anti-GAPDH (Milipore-MAB374, 1:5000 for Western Blotting) was purchased from Milipore (MA, USA). A549 cells were cultured in F12K medium containing 10% foetal bovine serum and 1% pen/strep in an incubator containing 5% CO_2_ at 37 °C. The drug was prepared with DMSO to a concentration of 5 mM; After the cells were plated for 24 h, the drug-containing medium was changed, and then the drugs were added to co-culture for 8 h, and then the cells were collected. Add 10 μL PMSF (100 mM) to 1 mL lysate, shake well and put it on ice (PMSF can be mixed with lysate only after shaking well until there is no crystallization). According to the number of cells, add 100–500 μL lysis solution containing PMSF to each tube of cells, and lyse on ice for 30 min. Centrifuge at 12,000 rpm for 10 min at 4 °C. The centrifuged supernatant was packaged and transferred to a 1.5 mL centrifuge tube for protein quantitative detection. The protein was pre-treated using BCA method. The protein solution was mixed with 5× loading buffer according to the volume ratio of 4:1, boiled in boiling water for 10 min, cooled and then subjected to SDS-PAGE electrophoresis.

According to the molecular weight of the target protein, 12% separation gel and 5% concentrated gel were prepared. Calculate the volume of solution containing 50 μg protein, which is the loading amount. Use 1× loading buffer to make up the total volume of each sample loading hole consistent; The voltage of concentrated glue is 80 V for 30 min, and the voltage of separated glue is 120 V for 30–50 min. Bromophenol blue can stop electrophoresis and transfer film when it reaches the glue bottom. PVDF membrane with constant pressure of 100 V, 60 min and 0.45 μm; Immerse PVDF membrane completely in 5% BSA-PBST, and gently shake at room temperature for 60 min. The primary antibody was diluted with 5% BSA-PBST and incubated overnight at 4 °C. The PVDF membrane was taken out the next day, and PBST washed the membrane five times, each time for 6 min. PBST diluted secondary antibody and incubated at room temperature for 60 min. PBST membrane washing 5 times and each time is 6 min. After mixing ECL A and B solution according to the volume of 1:1, drop them evenly on the film, set the exposure time and type as required, start the exposure, save the picture and export the picture after the exposure, and use image analysis software Image J for grey Analysis.

### 2.5. Wound healing assay

A549 cells (6 × 105 cells) were seeded in six-well plates and grown to approximately 100% confluence in culture medium. Subsequently, a cell-free line was manually created by scratching the confluent cell monolayers with a 200 μL pipette tip. The wounded cell monolayers were washed three times with phosphate-buffered saline (PBS) and incubated with serum-free medium. Then, cells were treated with different concentrations of compound **A13**, incubated for 72 h, and photographed at 0, 24 and 72 h with an inverted microscope.

### 2.6. Transwell invasion assay

On the first day, 0.2× basement membrane extract (BME) working solution was prepared by diluting 5× BME stock solution in 1× Travigen Inc. coating buffer. Briefly, 100 μL of 10× coating buffer was diluted in 900 μL of sterile water to make 1× coating buffer. Then 960 μL of 1× coating buffer was mixed with 40 μL of 5× BME to make a working 0.2× BME solution. Corning Transwell permeable inserts (Costar Transwell chambers, Corning) were placed on a 24-well plate, and 100 μL of 0.2× BME solution was added to each Transwell insert and incubated for 16 h. The following day, A549 cells were trypsinized and cells were suspended in serum-free medium. Approximately 100 μL from the cell suspension (∼3 × 105 cells) was added to each Transwell insert followed by another 100 μL of PROTAC or control containing serum-free RPMI medium. The lower chamber was filled with 10% FBS containing RPMI medium, and the whole setup was incubated at 37 °C and 5% CO_2_ for 24 h. After 24 h, the cell culture medium was removed from both lower and upper chambers and the Transwell inserts were washed three times with PBS. Non-invasive cells were removed using a cotton swab, and the bottom side of the membrane of the Transwell inserts was fixed with 4% formaldehyde for 10 min at room temperature followed by permeabilization with PBST (pH-7.4, 50 mM Tris-HCl, 150 mM NaCl, 0.1% Triton-X100) for another 10 min. Inserts were washed once with PBS and stained with 0.2% (w/v) crystal violet solution for 20 min at room temperature. Inserts were then extensively washed with PBS and once with water to remove all excess dye and salts. Cells migrated through the membrane were captured using a camera attached to a light microscope. Images were then analysed and the number of cells on the bottom side of the membrane were counted by ImageJ software.

### 2.7. Plasma stability assay

The pooled frozen human plasma (BioreclamationIVT, batch No. BRH1569252) was thawed in a water bath at 37 °C prior to experiment. Plasma was centrifuged at 4000 rpm for 5 min and the clots were removed if any. The pH will be adjusted to 7.4 ± 0.1 if required. 100 μM propantheline bromide solution was prepared by diluting 5 µL of 10 mM stock solution with 495 µL H_2_O as a positive control. The other detected compound solution was prepared by the same method. 98 µL of blank plasma was spiked with 2 μL of dosing solution (100 μM) to achieve 2 μM of the final concentration in duplicate and samples were incubated at 37 °C in a water bath. At each time point (0, 10, 30, 60 and 120 min), 400 μL of stop solution (200 ng/mL tolbutamide and 200 ng/mL Labetalol in ACN) was added to precipitate protein and mixed thoroughly. Centrifuged sample plates at 4000 rpm for 15 min. An aliquot of supernatant (50 μL) was transfered from each well and mixed with 100 μL ultra-pure water. The samples were shaked at 800 rpm for about 10 min before submitting to LC-MS/MS analysis.

### 2.8. The membrane permeability assay

After 21–28 days, the cells seeded on transwell filters were used for permeability experiments. The monolayers were rinsed once with a solution of 0.02% EDTA in DPBS on the apical and basolateral side, and three times with HBSS buffered at pH 7.4 with 25 mM HEPES buffer (*N*-2-hydroxyethylpiperazine-*N’*-2-ethane sulphonic acid). The cells were then left to equilibrate at 37 °C for 30 min in HBSS/HEPES solution. TEER values were then measured. All cell monolayers used had TEER values between 2.5 and 5 kΩ/cm^2^ before the experiments.

At the start of the experiment (time = 0 min), 600 μL of buffered HBSS had been placed on the basolateral side of the monolayer and 100 μL of 200 μM solution of the test compound prepared in HBSS/HEPES was added to the apical side of the monolayer. At time 30, 90, 120 and 150 min, 400 μL were sampled from the basolateral side. The volume sampled (400 μL) was replaced with 400 μL of HBSS/HEPES buffer solution. Each compound was tested on 4 wells/experiment and at least three experiments were conducted using different passage number cells. LC–MS/MS analysis was performed on each sample and compared to a standard curve (0.05–100 μM) to determine the concentration of each respective peptide derivative. Apparent permeability values (Papp) were then calculated using the following formula.

$$\mathrm{Papp}\;=\;\frac{dC}{dt}\;\times \;\frac{Vr}{A\;\times {\;C}_{0}}$$

where *dC*/*dt* is the steady state rate of the change in the chemical concentration (M/s); *Vr* is the volume of the receiver chamber (cm^3^); A is the surface area of the cell monolayer (cm^2^); C0 is the initial concentration in the donor chamber (M).

### 2.9. Molecular docking study

Ligand structures were prepared by using Maestro 9.0 within the Schrodinger package. The crystal structures of FAK (PDB ID: 3BZ3) were retrieved from RCSB Protein Data Bank (http://www. pdb.org), and prepared for molecular docking by using Protein Preparation Wizard. The ligand structures were optimised by Maestro’s Ligprep module, regulated to a protonated state of pH 7.4, and minimised with OPLS_2005 force field to produce low-energy conformers. Compounds were docked into binding sites with the Glide module within the Schrodinger package on account of united-atom scoring function. The docking program was implemented twenty times, providing sufficient constellation groups and the output was characterised by the favourable binding affinity value. In addition, the figure was prepared using PyMOL.

## 3. Experimental section

### 3.1. Chemistry

**Scheme 1.**
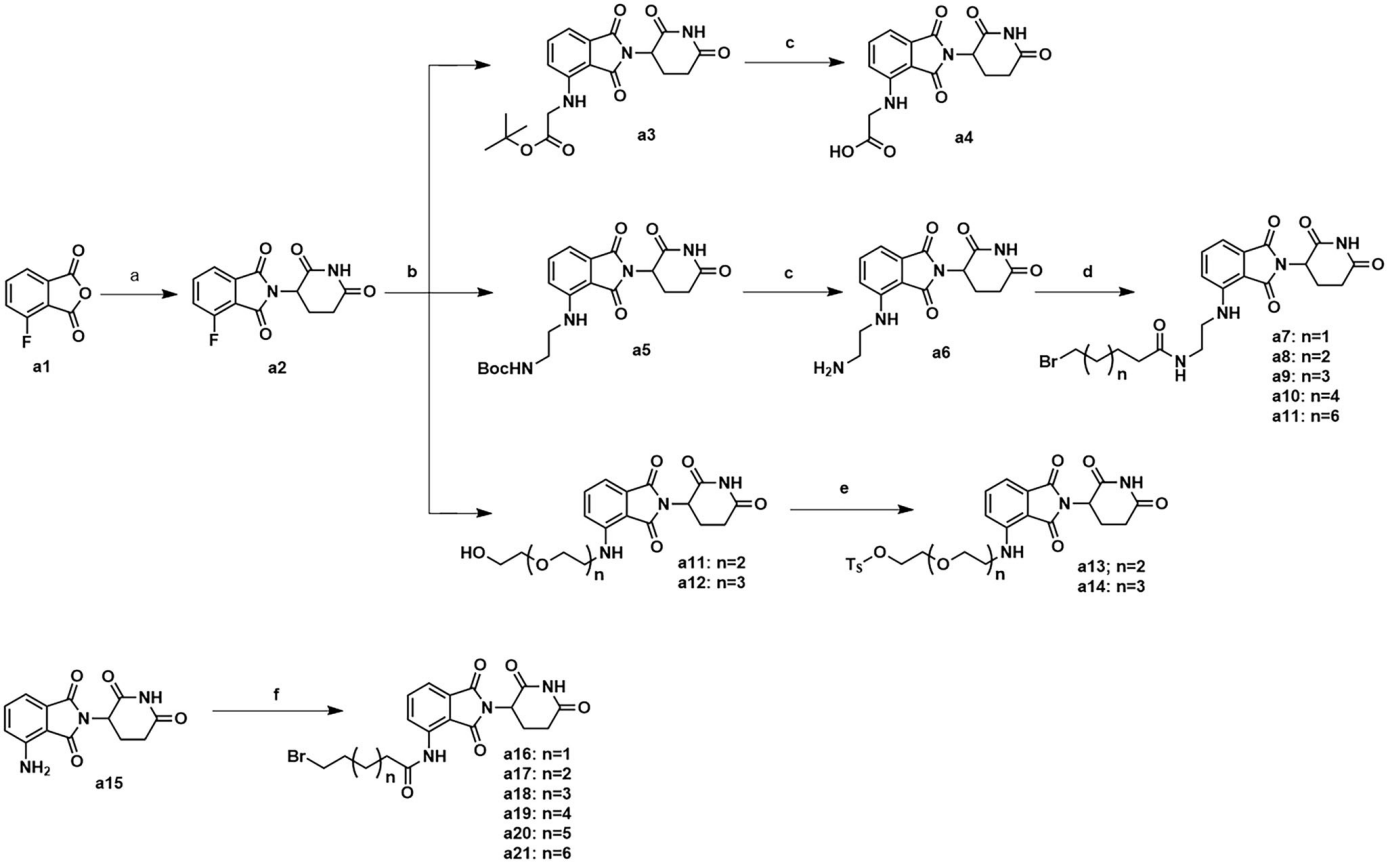
Synthesis of intermediates **a4**, **a7–a11**, **a13–a14** and **a16–a21**. Reagents and conditions: (a) 3-aminopiperidine-2,6-dione, NaOAc, AcOH, 140 °C, 58.3–68.7% yield; (b) DIPEA, DMF, 90 °C,53.6–65.2% yield; (c) CF_3_COOH, CH_2_Cl_2_, 25 °C, 86.3–90.2% yield; (d) HATU, DIPEA, 25 °C, 67.4–75.3% yield; (e) TsCl, Et_3_N, CH_2_Cl_2_, 30 °C, 72.6–79.3% yield; (f) corresponding bromocarboxylic acid, SOCl_2_, CH_2_Cl_2_, 40 °C, 61.5–85.8% yield.

The synthesis of intermediates **a4**, **a7–a11**, **a13–a14** and **a16–a21** was followed as Scheme 1. Commercially available 4-fluoroisobenzofuran-1,3-dione (**a1**) gave intermediate **a2** via cyclisation reaction with 3-aminopiperidine-2,6-dione. The F atom of intermediate **a2** was substituted by tert-butylglycine, followed by hydrolysis reaction to obtain intermediate **a4**. The F atom of intermediate **a2** was substituted by Boc-protected ethylenediamine to generate intermediate **a5**. After removing the Boc protecting group, intermediate **a5** was condensed with the corresponding long chain of carboxylic acid to acquire intermediates **a7–a11**. Nucleophilic substitution of intermediate **a2** with amino-substituted PEG-chains gave intermediates **a11–a12**. And intermediates **a11–a12** were protected by TsCl to afford intermediates **a13–a14**. Commercially available **a15** was condensed with the corresponding long chain of carboxylic acid to obtain intermediates **a16–a21**.

**Scheme 2.**
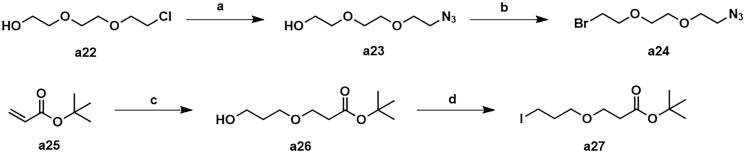
Synthesis of intermediates **a24** and **a27**. Reagents and conditions: (a) NaN_3_, DMF, 100 °C, 47.5% yield; (b) CBr_4_, PPh_3_, CH_2_Cl_2_, 25 °C, 53.7% yield; (c) propane-1,3-diol, tritionB, CH_3_CN, 25 °C, 36.2% yield; (d) I_2_, PPh_3_, imidazole, THF, 25 °C, 31.8% yield.

The synthetic routes for intermediates **a24** and **a27** were shown in Scheme 2. Using 2-[2–(2-chloroethoxy)ethoxy]ethan-1-ol (**a22**) as the starting material, the intermediate **a24** was generated by azide and bromination. The addition reaction of tert-butyl acrylate with 1,3-propanediol gave intermediate **a26**, which was then substituted by iodine to afford intermediate **a27**.

**Scheme 3.**
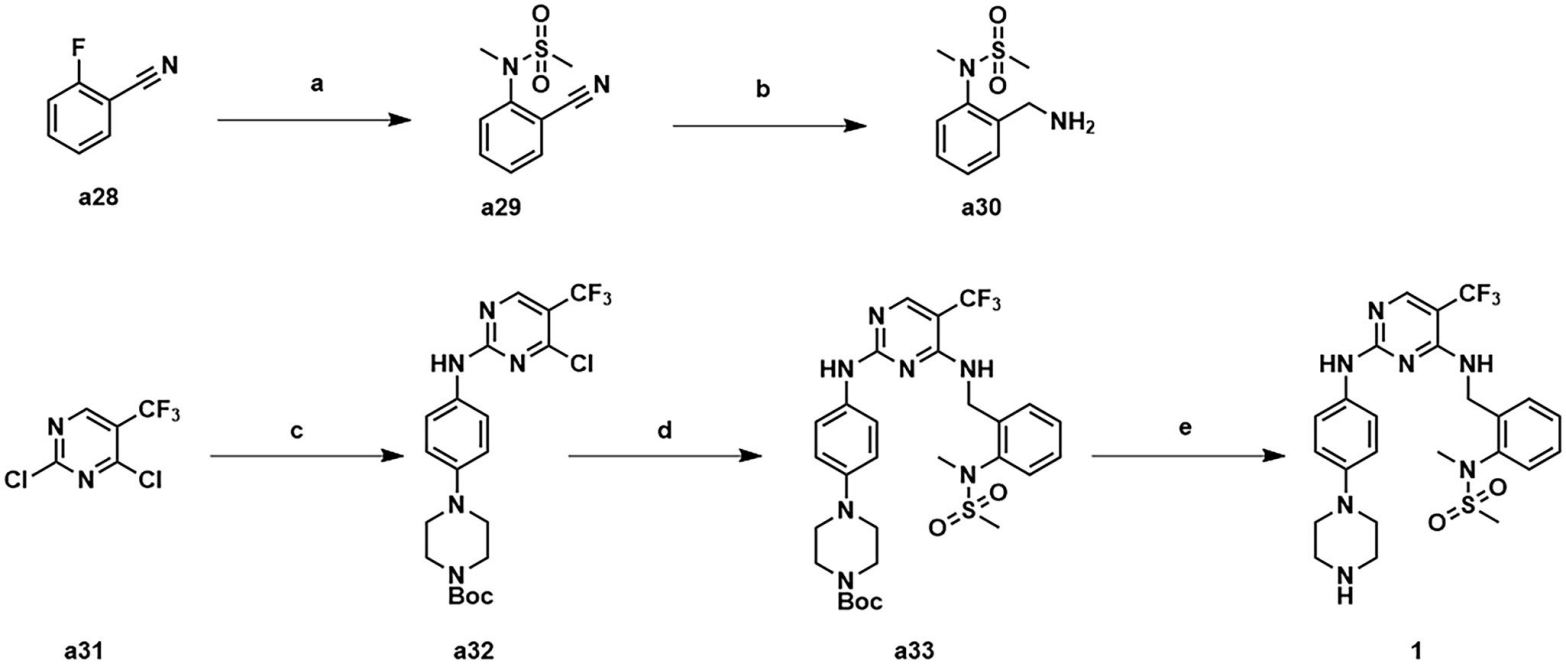
Synthesis of compound **1**. Reagents and conditions: (a) *N*-methylmethanesulfonamide, Cs_2_CO_3_, CH_3_CN, 80 °C, 82.6% yield; (b) BH_3_ (2 M in THF), anhydrous THF, 60 °C, 47.9% yield; (c) tert-butyl 4–(4-aminophenyl)piperazine-1-carboxylate, ZnBr_2_, TEA, t-BuOH/DCE, 0 °C, 72.3% yield; (d) **a30**, DIPEA, 1,4-dioxane, 100 °C, 81.3%; e) CF_3_COOH, CH_2_Cl_2_, 25 °C, 93.6% yield.

Compound **1** was synthesised as depicted in Scheme 3. Commercially available *o*-fluorobenzonitrile was substituted by *N*-methyl-methanesulfonamide to give intermediate **a29**, which was then subjected to a reduction to obtain intermediate **a30**. Commercially available 2,4-dichloro-5-(trifluoromethyl)pyrimidine **a31** afforded the intermediate **a33** through a two-step substitution reaction, and was followed by a deprotection to produce compound **1**.

**Scheme 4.**
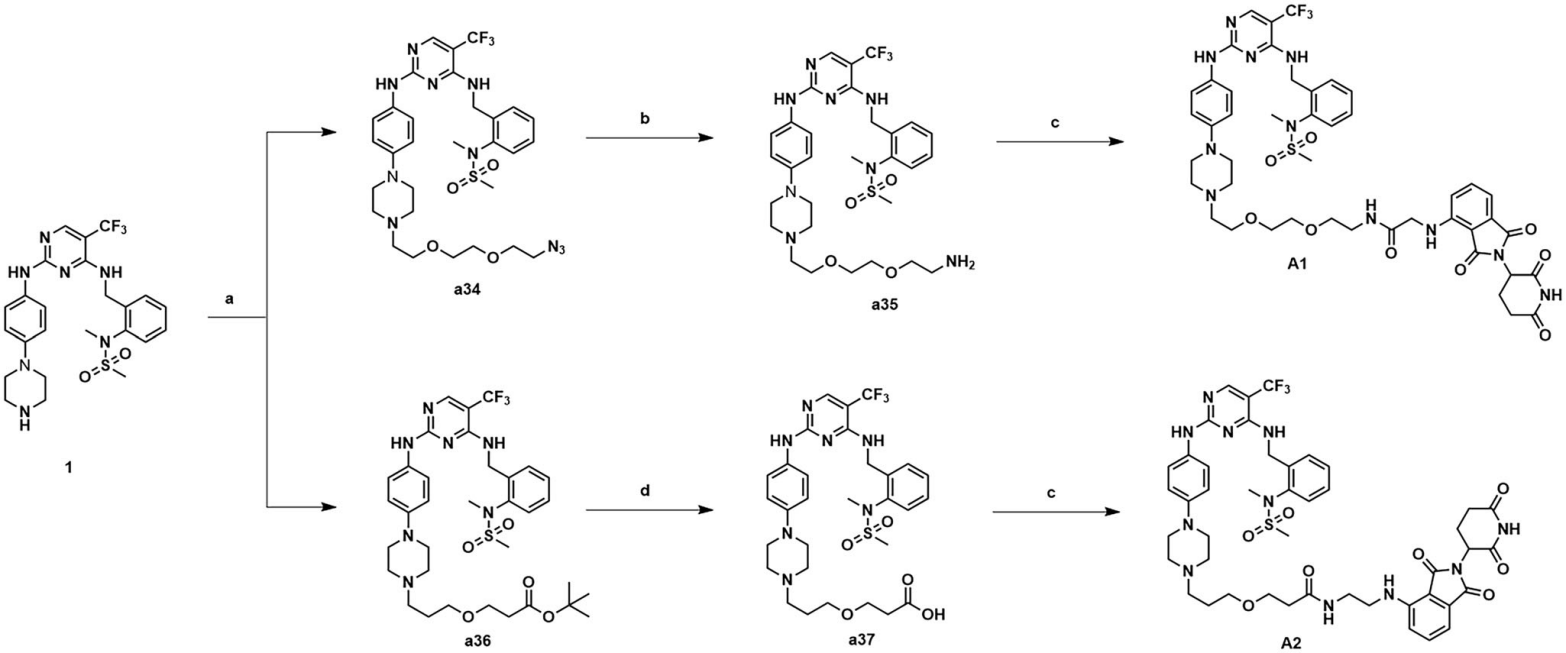
Synthesis of compounds **A1-A2**. Reagents and conditions: (a) **81** or **84**, K_2_CO_3_, CH_3_CN, 80 °C, 58.6–63.3% yield; (b) PPh_3_, THF/H_2_0, 70 °C, 76.3–81.2% yield; (c) **65** or **67**, HATU, DIPEA, CH_2_Cl_2_, 25 °C, 31.7–36.8% yield; (d) TFA, DCM, 30 °C, 95% yield.

The synthetic routes of compounds **A1-A2** are shown in Scheme 4. Compound **1** was substituted by intermediate **a24** to give intermediate **a34**. Intermediate **a34** was reduced to a primary amine and condensed with intermediate **a4** to obtain compound **A1**. Compound **1** was substituted by intermediate **a27** to give intermediate **a36**, which was hydrolysed and condensed with intermediate **a6** to obtain compound **A2**.

**Scheme 5.**
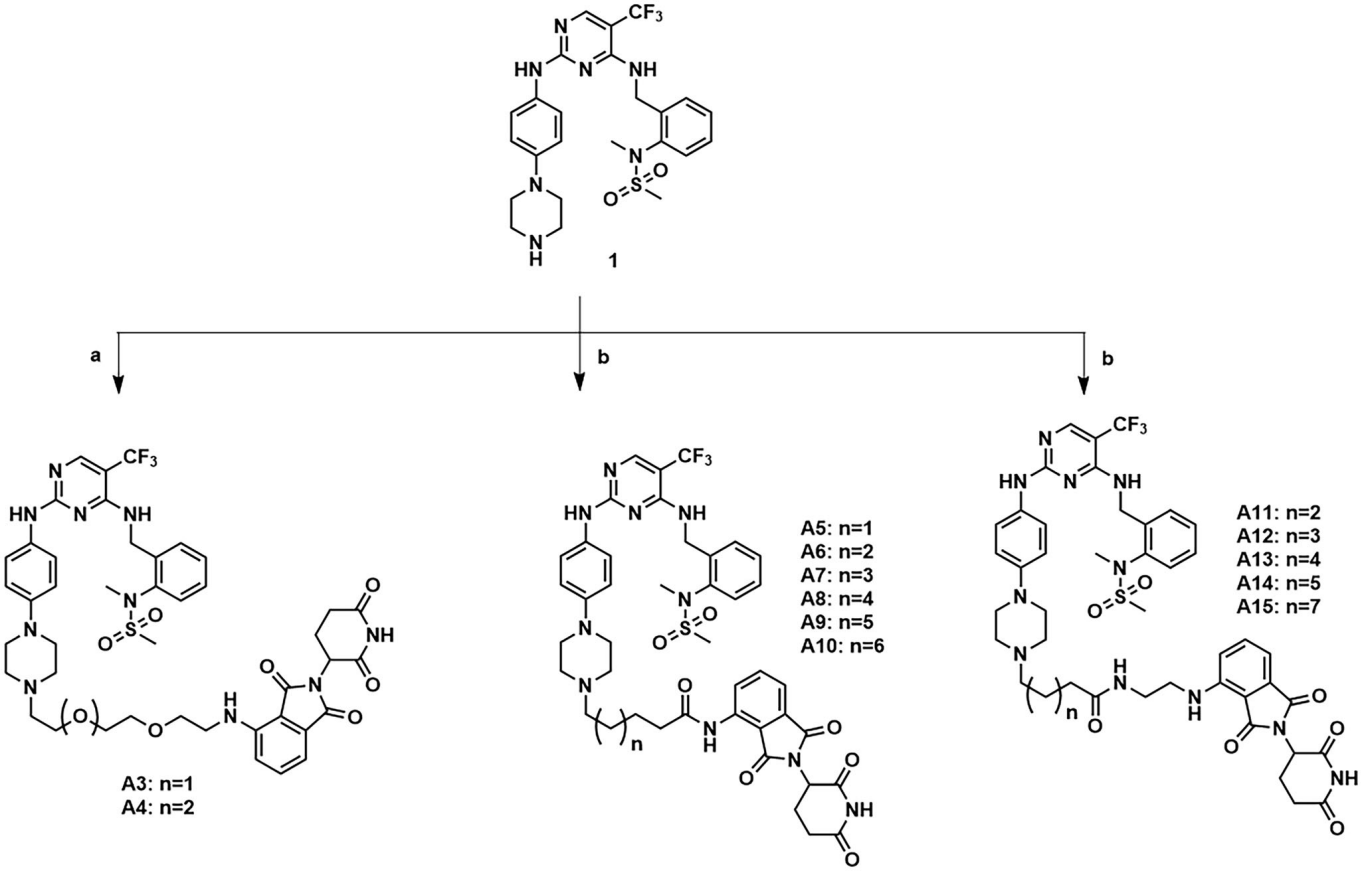
Synthesis of compounds **A3–A15**. Reagents and conditions: (a) **a13–a14**, DIPEA, DMF, 90 °C, 29.6–37.7%; (b) **a16–a21** or **a7–a11**, KI, K_2_CO_3_, CH_3_CN, 80 °C, 29.4–41.9%.

The compounds **A3–A15** were synthesised as shown in Scheme 5. Compound **1** was substituted by intermediates **a13–a14**, **a16–a21** and **a7–a11** to afford compounds **A3–A4**, **A5–A10** and **A11–A15**.

### 3.2. In vitro *activity against FAK kinase and SAR analyses*

**Figure 4.**
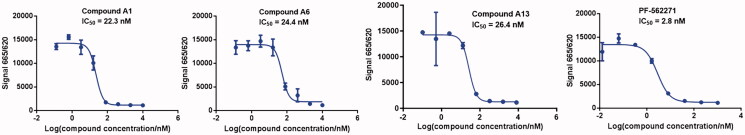
The enzyme inhibition activity curve of compound **A1**, **A6**, **A13** and **PF-562271**.

Compounds **A1–A15** were evaluated for their activities against FAK kinases using homogeneous time-resolved technology (HTRF) assays at concentrations of 0.5 μM and 0.1 μM. PF-562271 was served as a positive control to confirm the experimental condition. PF-562271 showed a strong inhibitory activity against FAK with an IC_50_ value of 2.8 nM (Figure 4), which was similar to the reported data. As shown in Table 1, the results from the assay showed that inhibitory activities of most of PROTACs were more than 90% at the concentration of 0.5 μM. Furthermore, at the concentration of 0.1 μM, all PROTACs inhibited FAK protein by more than 50%, indicating that all the PROTACs had good affinities to FAK protein. In addition, compounds A1, A6 and A13 containing alkyl-linker or PEG-linker were selected to test their IC_50_ values, both of them exhibited good inhibitory activities with IC_50_ values of 22.3, 24.4 and 26.4 nM, respectively, which were slightly lower than PF-562271 (Figure 4).

**Table 1.** FAK inhibition of compound **1** based PROTACs **A1**–**A15** with different linkers. 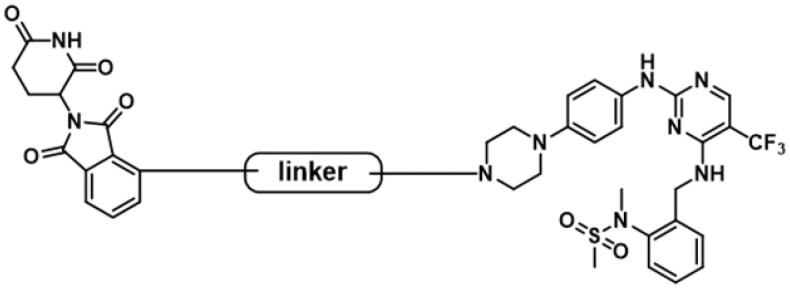

Compd.	linker	FAK inhibition (%)^a^	
		0.5 μM	0.1 μM
**A1**	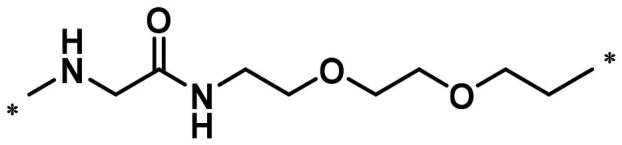	98.0	94.9
**A2**	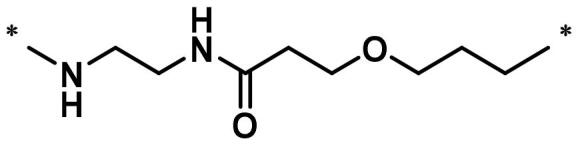	98.8	89.0
**A3**	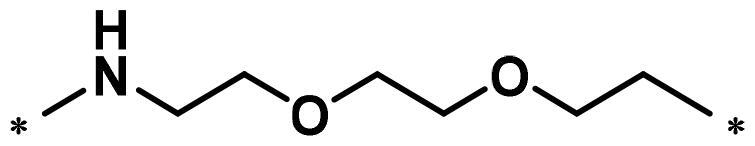	97.5	87.8
**A4**	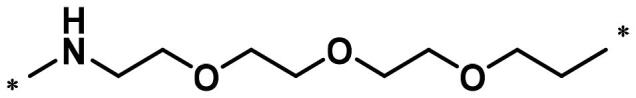	97.0	90.8
**A5**	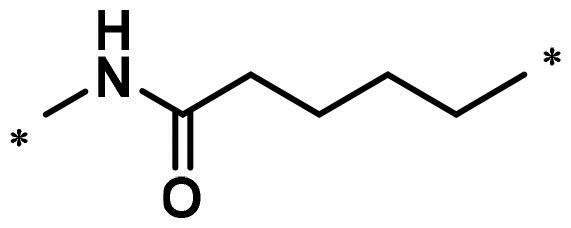	98.0	91.2
**A6**	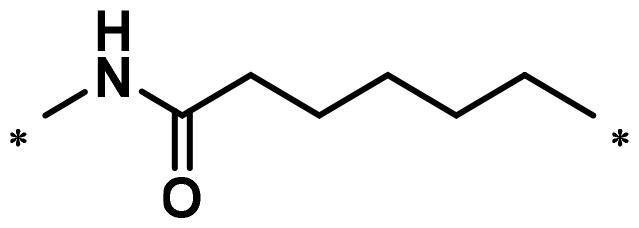	96.8	88.2
**A7**	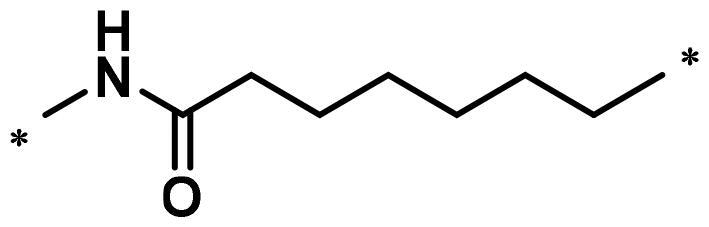	95.4	87.5
**A8**	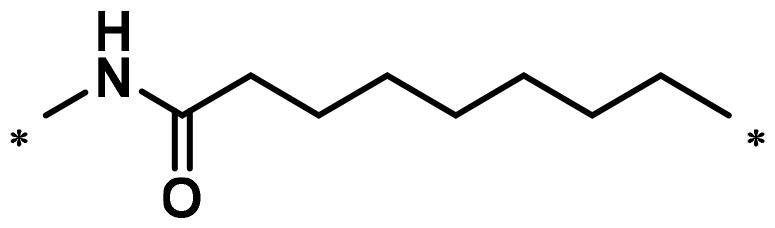	83.0	67.7
**A9**	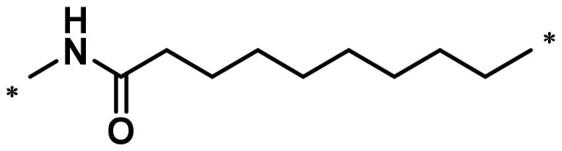	92.3	79.0
**A10**	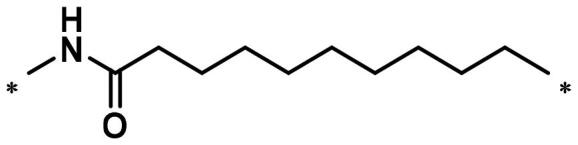	90.0	66.3
**A11**	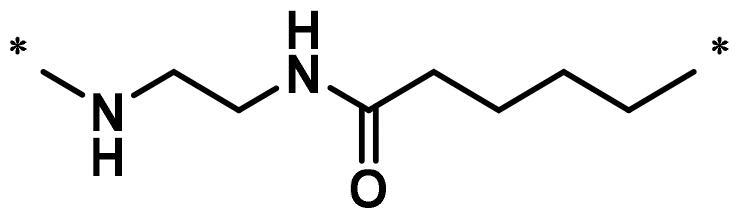	98.6	93.5
**A12**	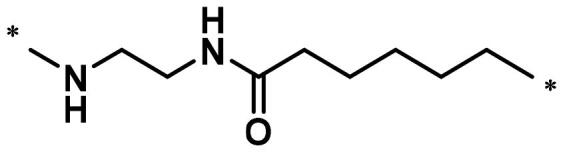	98.0	86.1
**A13**	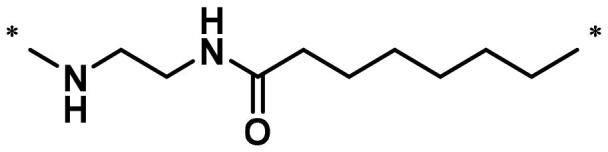	96.9	84.4
**A14**	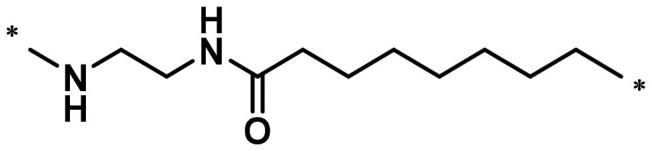	92.8	84.0
**A15**	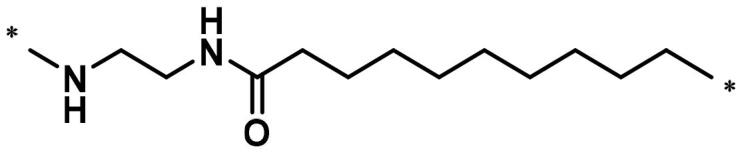	95.8	74.9
**PF-562271**		95.6	91.2

^a^The inhibition ratio are shown as the mean values from two separate experiments.

**Figure 5.**
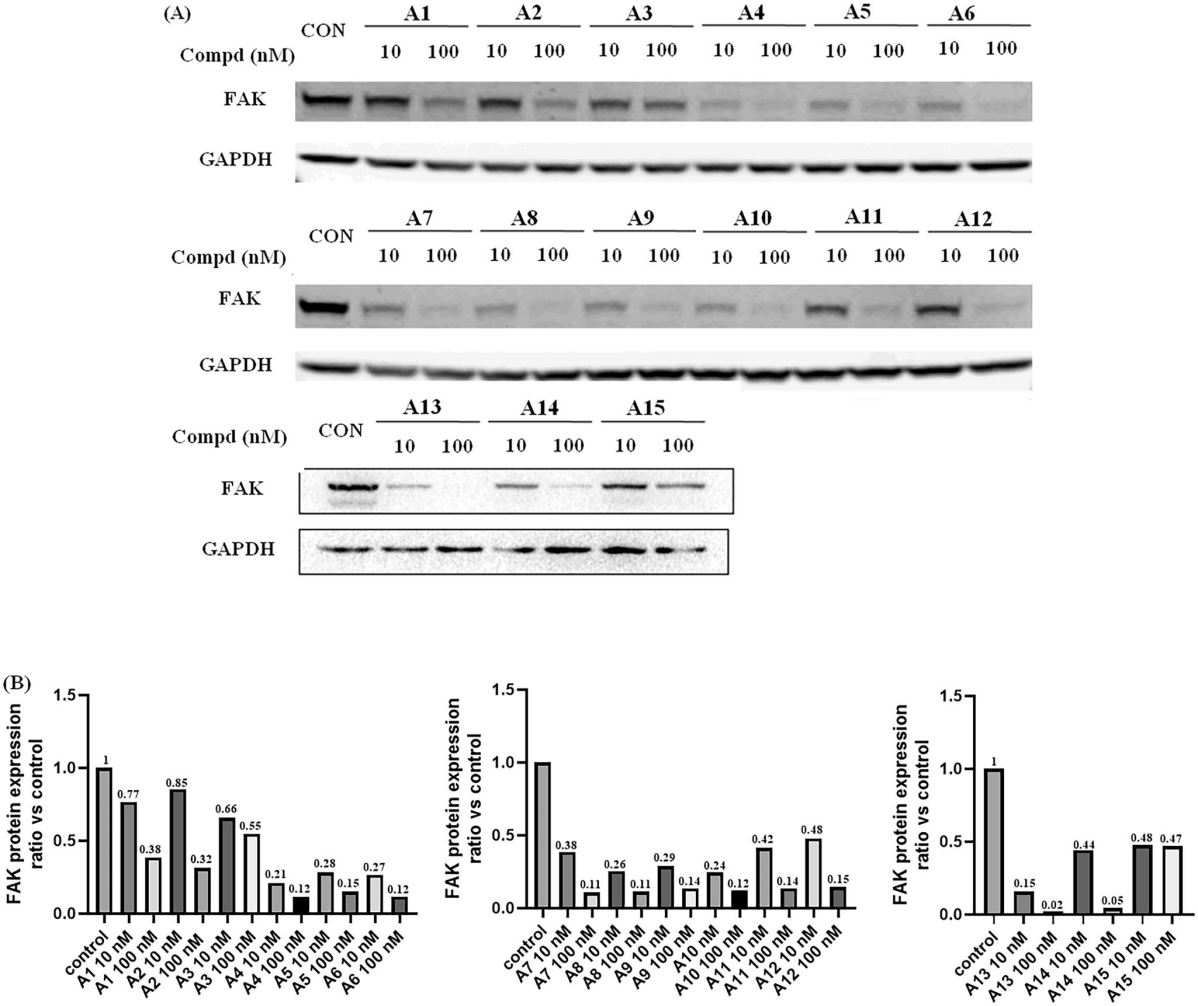
FAK-degrading efficiency of PROTACs **A1–A15** in A549 cells (A) FAK levels in response to dose escalations of **A1–A15** in the A549 cell line after treatment for 12 h, with glyceraldehyde-3-phosphate dehydrogenase (GAPDH) used as the loading control. The experiments were repeated three times, and representative images were selected. (B) FAK protein expression ratio vs control.

Next, the degradation efficiency of FAK-PROTAC was evaluated in A549 cells. A549 was a human non-small cell lung cancer cell with high expression of FAK. We selected two concentration gradients (10 nM, 100 nM) of PROTACs to incubate with A549 cells, and then the cells were lysed and performed Western blot experiments to observe the changes of FAK protein content in A549 cells. As shown in Figure 5, the results of Western blot test showed that most of the compounds had potent degradation activities on FAK protein in A549 cells. Compounds **A4–A15** had stronger degradation activities, the degradation rate of FAK protein was more than 50% at concentration of 10 nM. Among them, compound **A13** exhibited optimal protein degradation (85% degradation at 10 nM).

### 3.3. The cell proliferation inhibition potency of PROTACs in A549 cells

**Figure 6.**
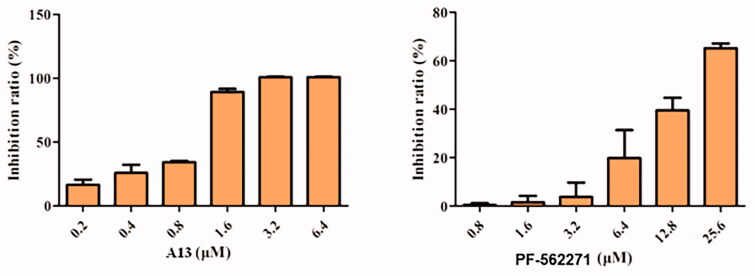
Antiproliferative activities of compound **A13** and PF-562271 against A549 cells. Cell growth inhibition rate was measured by MTT assay. (mean ± SD, *n* = 3).

To further test the antitumor activities of this class of PROTACs, we selected compound **A13** for antiproliferative activity studies against the A549 cell lines^23^. PF-562271 was used as a positive control. As shown in Figure 6, compound **A13** exhibited more significant anti-tumour cell proliferation inhibitory activity than PF-562271. At a concentration of 1.6 μM, the inhibition rate of compound **A13** was more than 50%, but the inhibition rate of PF-562271 was less than 20%. And at a concentration of 3.2 μM, the inhibition rate of compound **A13** was reached 100%. In contrast, the inhibition rate of PF-562271 was only more than 50% at a concentration of 25.6 μM.

### 3.4. Effects of PROTAC A13 on cell migration

**Figure 7.**
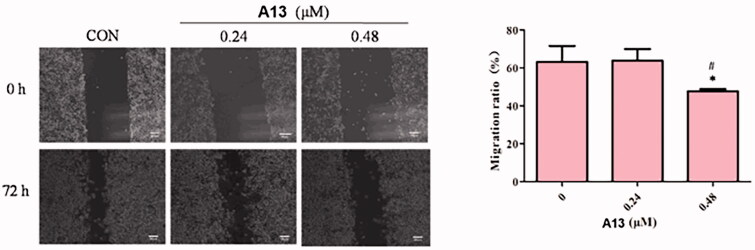
(A) The wound healing assay was performed to show the effect of indicated concentrations of PROTAC **A13** on A549 lung cancer cell migration. (B) Quantitative analysis of the wound healing assay by PROTAC **A13**. The migration ratio of A549 cells treated with **A13** was measured by wounding healing assay. (mean ± SD, *n* = 3). *p < 0.05, **p < 0.01 vs CON, ^#^*p* < 0.05.

To evaluate the effect of PROTAC **A13** on the migration of A549 cells, wound healing assays were performed. For this assay, A549 cells were incubated with different concentrations of **A13** (0.24 and 0.48 μM) and DMSO for 72 h, investigating whether compound **A13** affects cell lines migration after scratching the cell monolayer (Figure 7). The results from the assay showed that A549 cells filled more than 60% wounded area after scribing 72 h at the concentration of 0.24 μM of PROTAC **A13**. And at the concentration of 0.48 μM, the migration rate of A549 cells was less than 60%, a concentration-dependent inhibition of **A13** was observed in A549 cells.

### 3.5. Effects of PROTAC A13 on cell invasion

**Figure 8.**
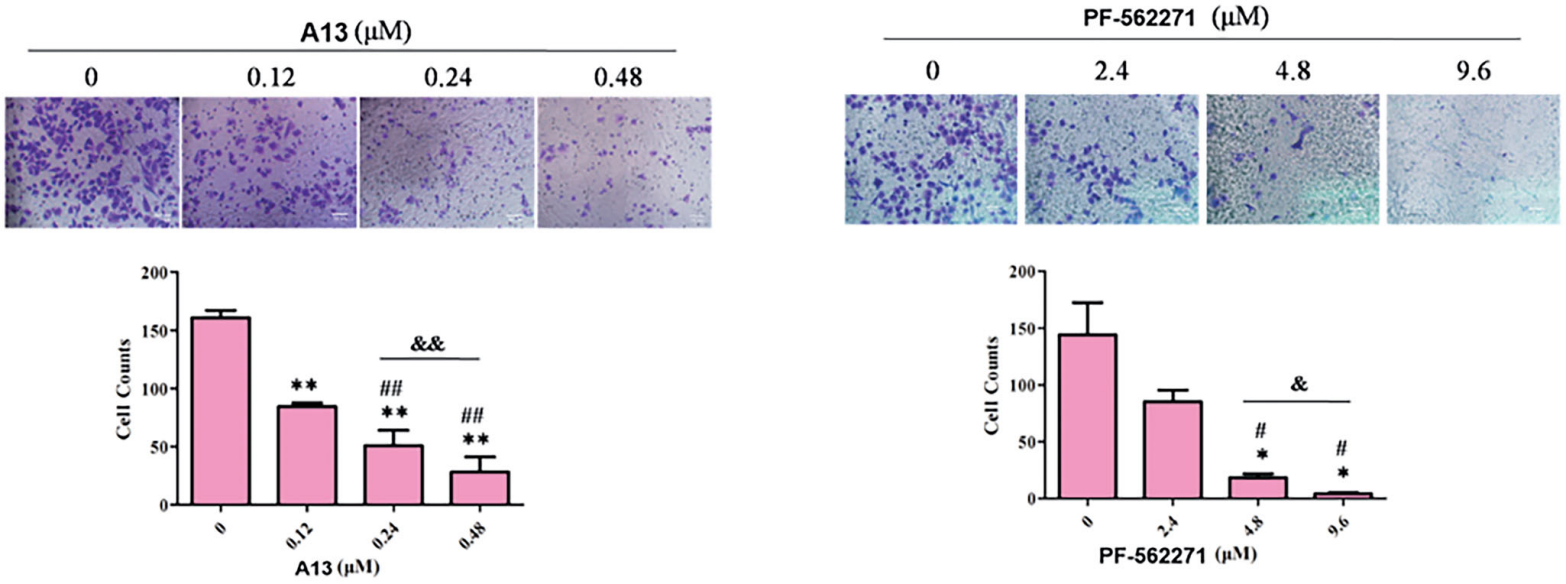
(A) Transwell assay was performed to show the effect of indicated concentrations of PROTAC **A13** and PF-562271 on A549 lung cancer cell invasion. (B) Quantitative analysis of transwell assay by PROTAC **A13** and PF-562271. The Invasion ratio of A549 cells treated with **A13** or PF-562271 was measured by transwell assay. (mean ± SD, *n* = 3). *p < 0.05, **p < 0.01 vs CON; ^#^*p* < 0.05, ^##^*p* < 0.01 vs 1/8 IC_50_; ^&^*p* < 0.05, ^&&^*p* < 0.01 vs 1/4 IC_50_.

We further investigated the inhibitory activity of PROTAC **A13** on the invasion of A549 cells, transwell assay was performed. PF-562271 was used as a positive control. A549 cells were incubated with DMSO, **A13** (0.12, 0.24 and 0.48 μM) and PF-562271 (2.4, 4.8 and 9.6 μM) for 72 h. The results were shown in Figure 8, the number of A549 cells that penetrated the membrane were decreased in a concentration-dependent manner, indicating that compound **A13** can dose-dependently inhibit the invasion of A549 cells. In addition, under the concentration of 0.24 μM of **A13**, the number of A549 cells that penetrated the membrane was lower than that of PF-562271 at the concentration of 2.4 μM, suggesting that the anti-invasion ability against A549 cells of PROTAC **A13** was superior than PF-562271.

### 3.6. The plasma stability and membrane permeability of PROTAC A13

Although PROTAC **A13** had been shown to degrade FAK, the high molecular weight and multiple hydrogen bond donors (HBDs) and acceptors (HBAs) of **A13** consistently limit their physicochemical properties, such as membrane permeability and stability^24–26^. Hence, the membrane permeability and *in vitro* plasma stability PROTAC **A13** were also evaluated. As shown in Table 2, compound A13 possessed favourable plasma stability with *T*_1/2_ > 194.8 min. But it is regrettable that PROTAC A13 exhibited a low membrane permeability with the measured Caco-2 permeability P_app(A−b)_ < 0.48 × 10^−6 ^cm/s, which is below the standard for “modest” permeability (P_app_ > 0.5 × 10^−6 ^cm/s) (Table 3).

**Table 2.** Plasma stability of PROTAC **A13**.

Time Point (min)	0	10	30	60	120
Remaining (%)	100.0	98.6	84.1	79.7	65.3
*T*_1/2_ (min)	>194.8				

**Table 3.** The membrane permeability of PROTAC **A13** in Caco-2 cell lines.

Compd.	A–B permeability (P_app_ × 10^-6^ cm/s)^a^	B-A permeability (P_app_ × 10^-6^ cm/s)^a^	Efflux ratio (P_app(B−A)_/ P_app(A−B)_)
**B5**	<0.480	0.417	>0.869

^a^Duplicates were performed (*n* = 2).

## 4. Conclusion

We designed and synthesised 15 FAK-targeting PROTACs based on FAK inhibitor (PF-562271 derivative **1**) and CRBN E3 ligase ligand (Pomalidomide). All PROTACs potently suppressed the enzymatic activities of FAK, the inhibition rate against FAK was more than 50% at the concentration of 0.1 μM. And PROTACs **A1**, **A6** and **A13** exhibited potent enzyme inhibition with IC_50_ values of 22.3, 24.4 and 26.4 nM, respectively. Next, the FAK degradation activities of 15 PROTACs were evaluated, the representative PROTAC **A13** had a degradation rate of 85% at the concentration of 10 nM. In addition, the antiproliferative activity by PROTAC **A13** exceeded beyond FAK inhibitor PF-562271 at a concentration of 1.6 μM in A549 cells, the inhibition rate of PROTAC **A13** was more than 50%. Also, the anti-invasion activity against A549 cells of PROTAC **A13** was superior than PF-562271. Further, preliminary drug-like properties evaluations indicate that PROTAC **A13** has excellent plasma stability (*T*_1/2_ > 194.8 min). In summary, PROTAC **A13** can be used as a potent tool to study FAK-related biology, and facilitated the development of new therapeutic agents for the treatment of FAK-related diseases.

## Supplementary Material

Supplemental Material
