# Supplementary material for: Design, synthesis, and biological evaluation of potent FAK-degrading PROTACs
Source: J Enzyme Inhib Med Chem. 2022 Aug 17;37(1):2241–55. doi: 10.1080/14756366.2022.2100886 (PMC9455338; doi:10.1080/14756366.2022.2100886)
Supplement: Supplemental Material [file IENZ_A_2100886_SM8975.pdf]

Supporting information

Design, synthesis, and biological evaluation of potent FAK-degrading PROTACs

Qiaohua Qin[a,1], Ruifeng Wang[a,b,1], Qinglin Fu[a], Guoqi Zhang[a], Tianxiao Wu[a], Nian Liu[a], Ruicheng Lv[a], Wenbo Yin[a], Yin Sun[a], Yixiang Sun[a], Dongmei Zhao[a,*], Maosheng Cheng[a]

[a]*Key Laboratory of Structure-Based Drug Design and Discovery, Ministry of Education, School of Pharmaceutical Engineering, Shenyang Pharmaceutical University, 103 Wenhua Road, Shenhe District, Shenyang 110016, PR China*

[b]*Department of Pharmacy, Shanxi Medical University, 56 Xinjiang Road, Yingze District, Taiyuan 030001, PR China*

CONTACT: Dongmei Zhao *medchemzhao@163.com* Shenyang Pharmaceutical University, 103 Wenhua Road, Shenhe District, Shenyang 110016, PR China

[1]Both authors contributed equally to this work.

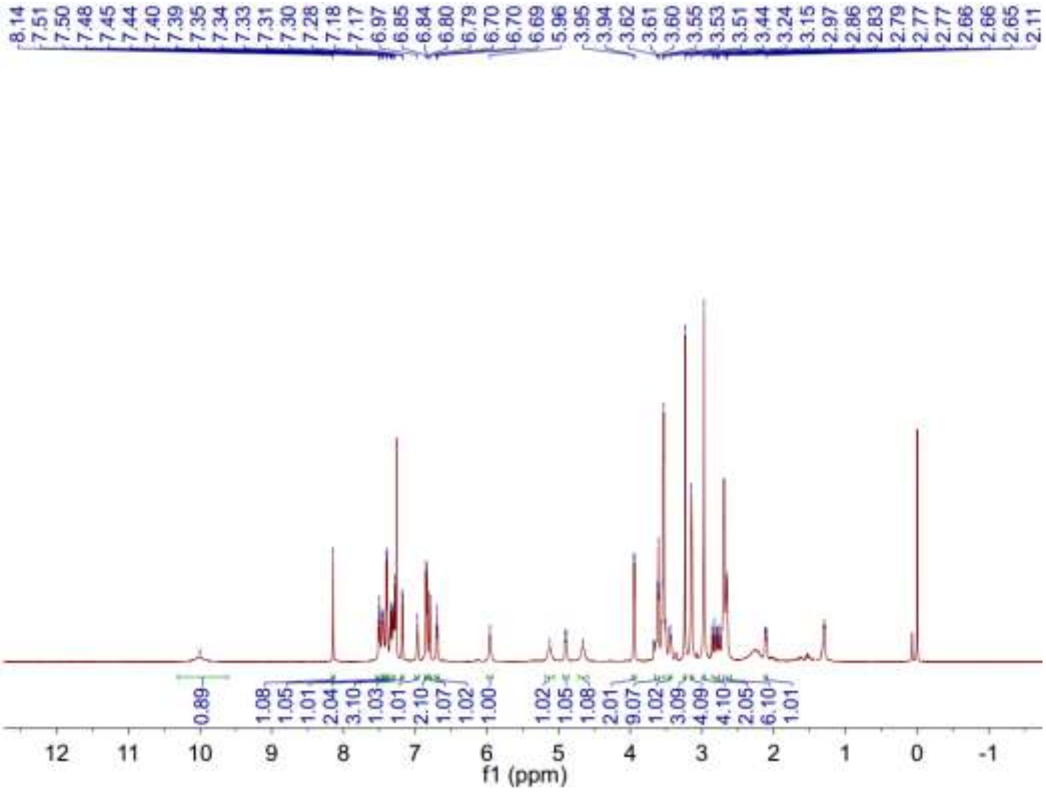

Figure S1. The ^{1}H-NMR spectrum of Compd. **A1**

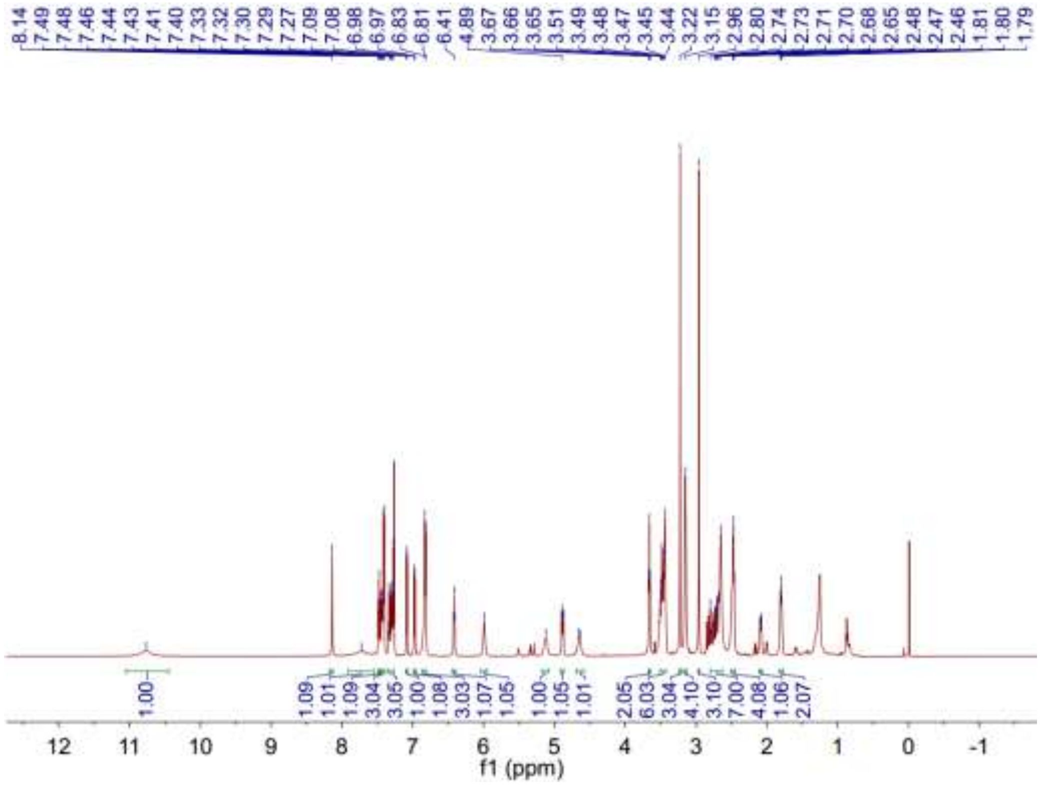

Figure S2. The ^{1}H-NMR spectrum of Compd. **A2**

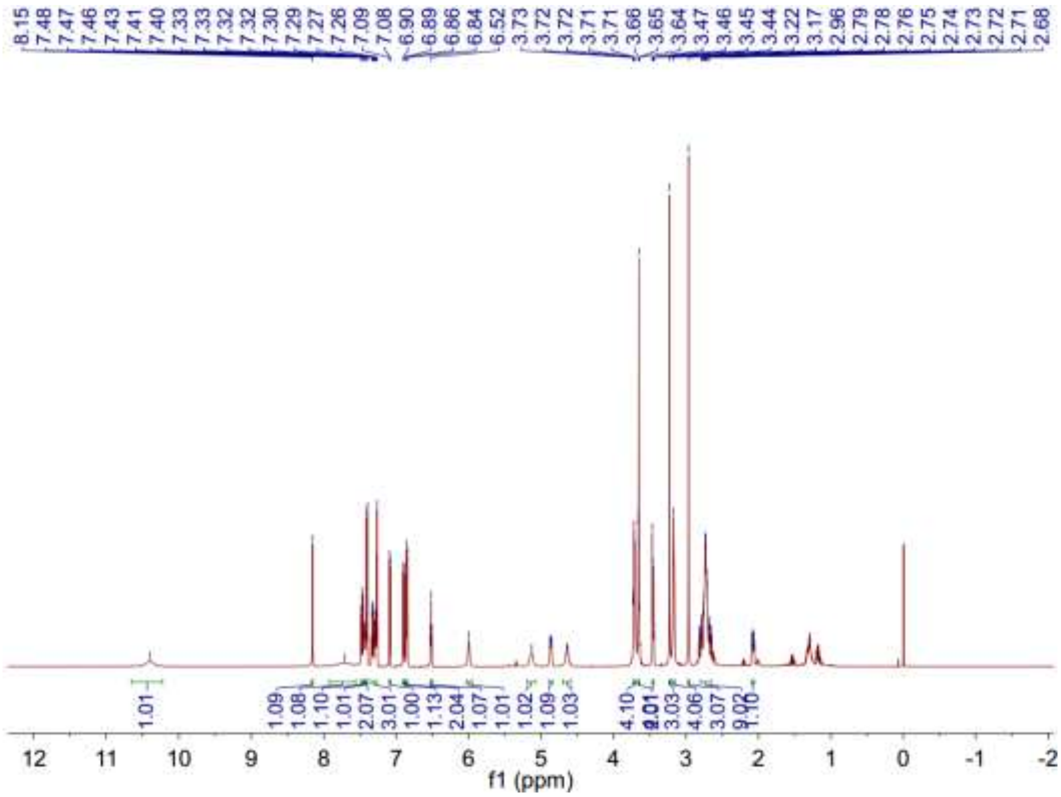

Figure S3. The ^{1}H-NMR spectrum of Compd. **A3**

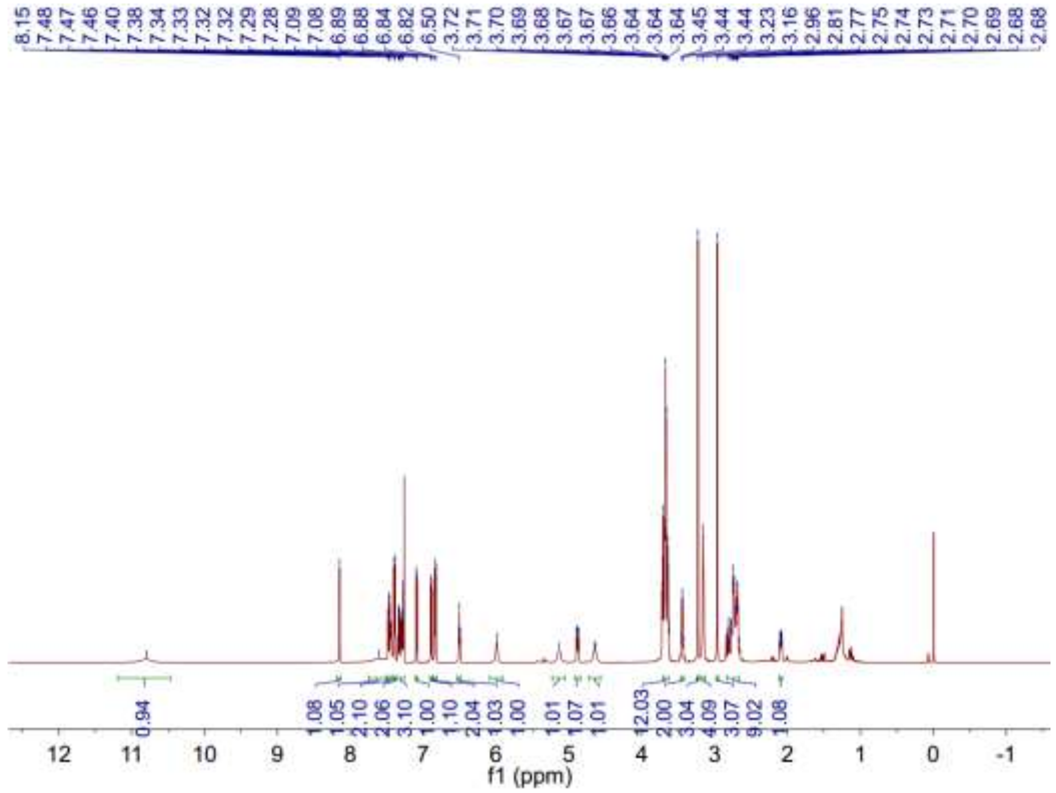

Figure S4. The ^{1}H-NMR spectrum of Compd. **A4**

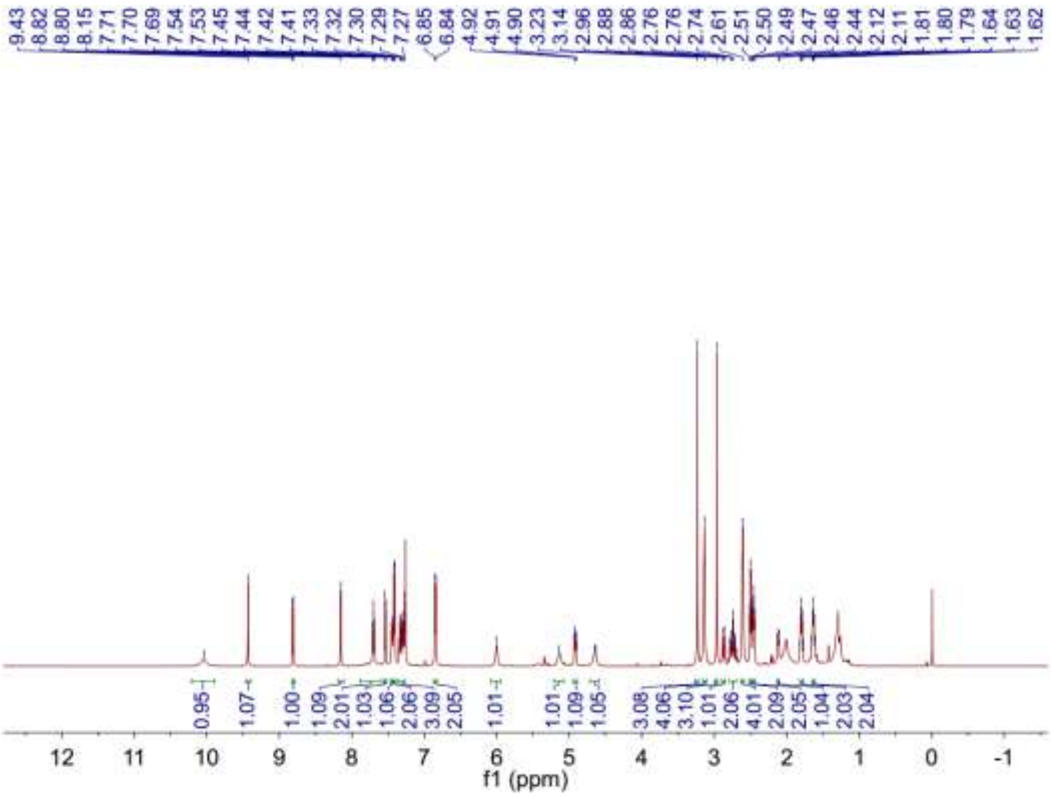

Figure S5. The ^{1}H-NMR spectrum of Compd. **A5**

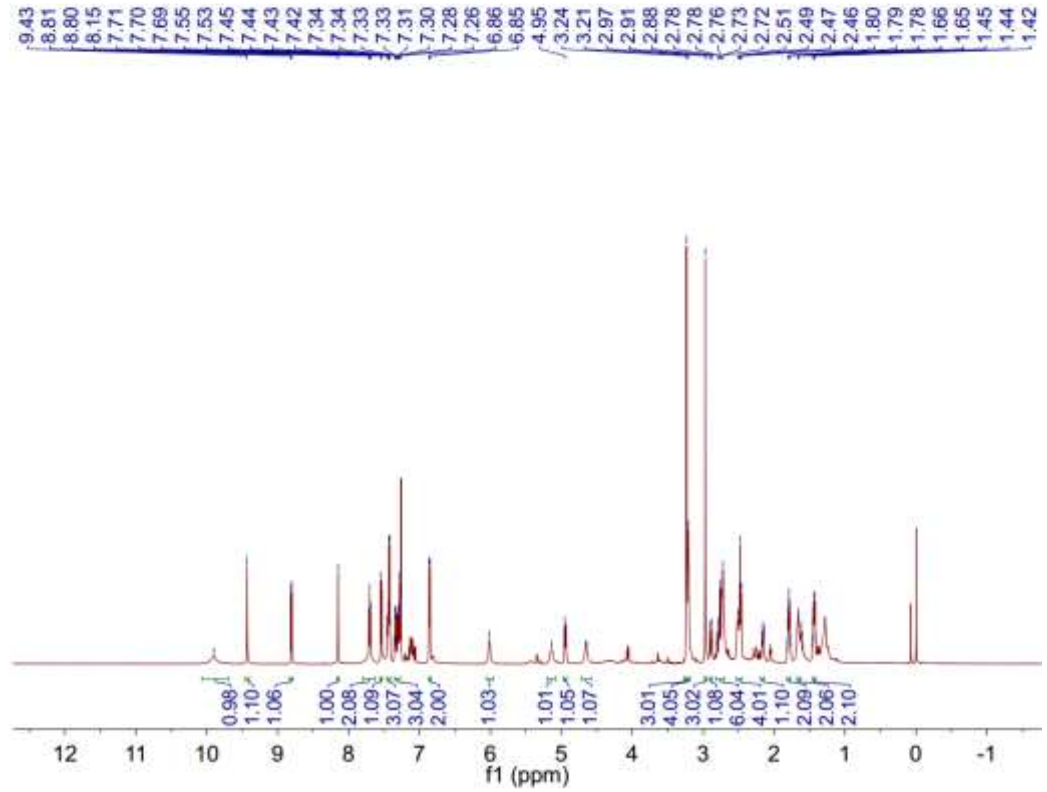

Figure S6. The ^{1}H-NMR spectrum of Compd. **A6**

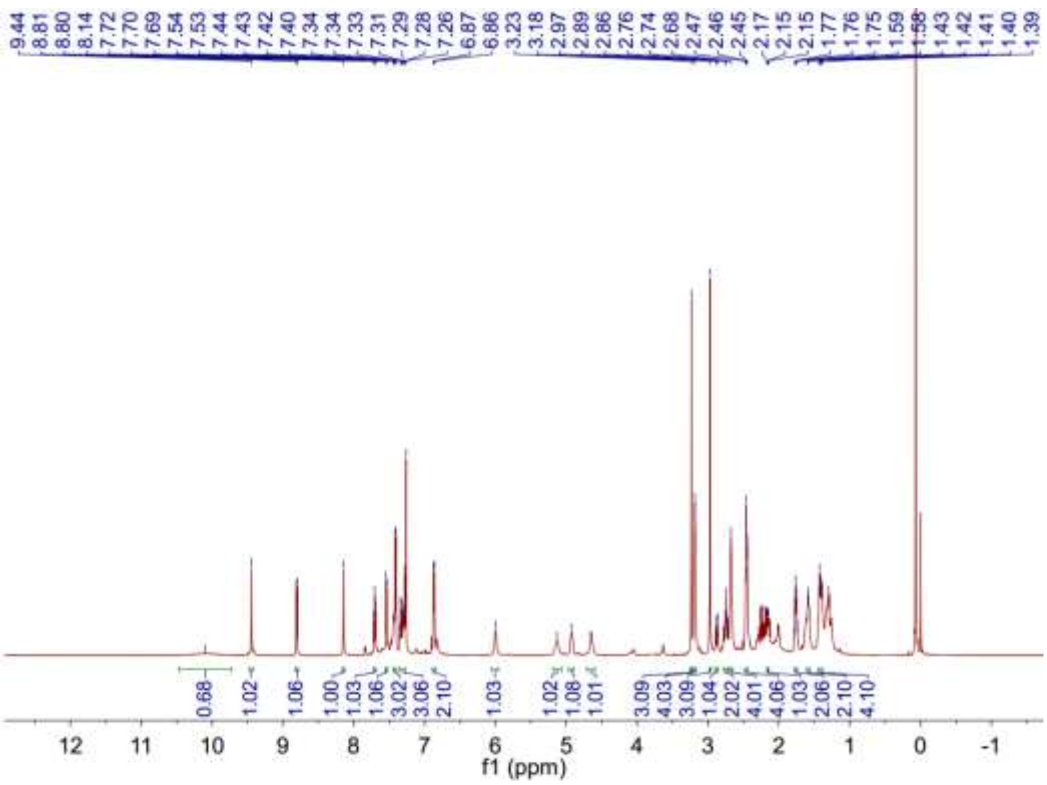

Figure S7. The ^{1}H-NMR spectrum of Compd. **A7**

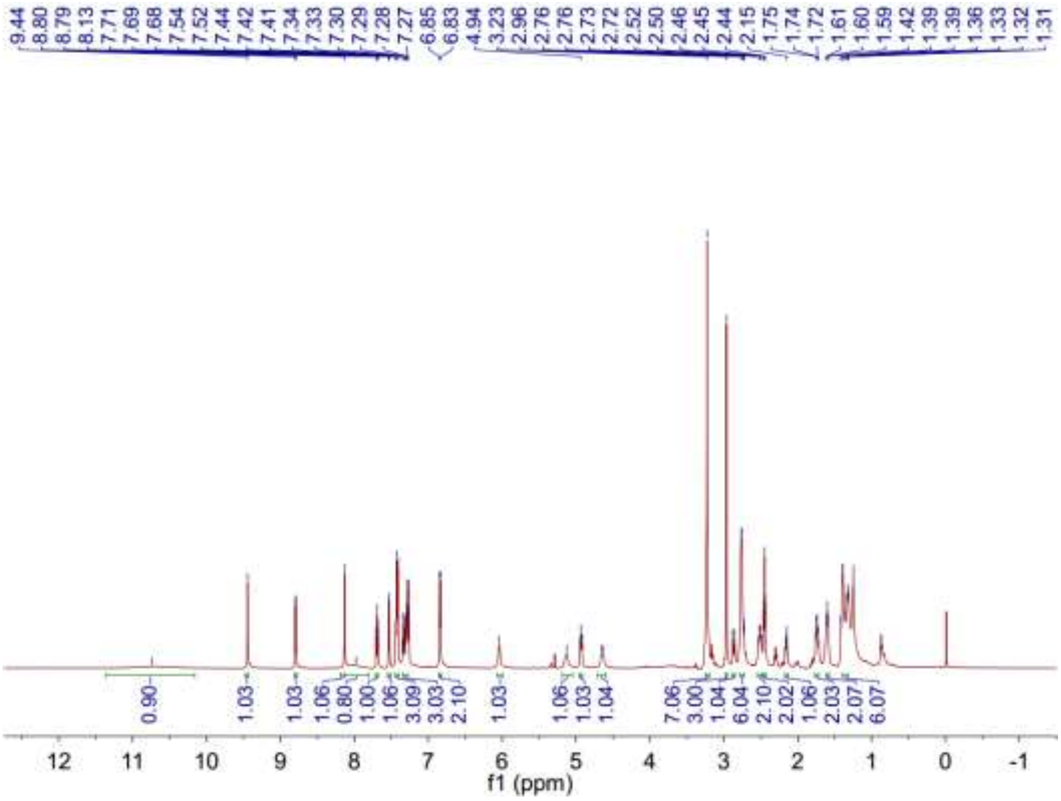

Figure S8. The ^{1}H-NMR spectrum of Compd. **A8**

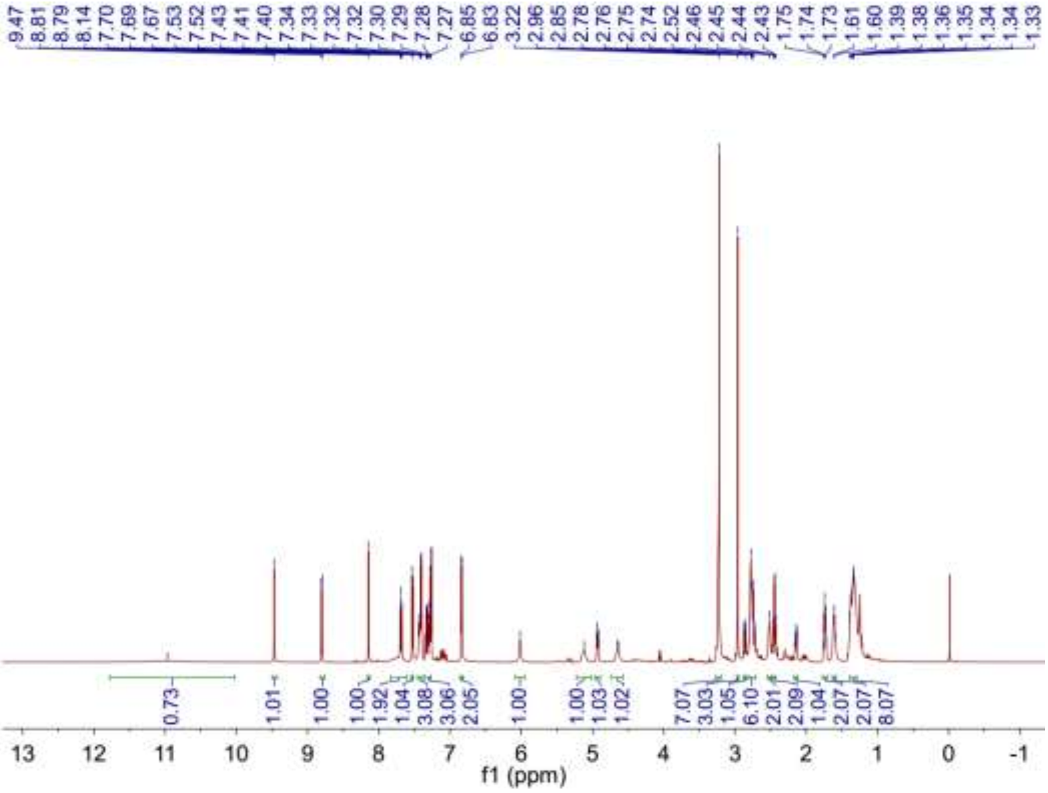

Figure S9. The ^{1}H-NMR spectrum of Compd. **A9**

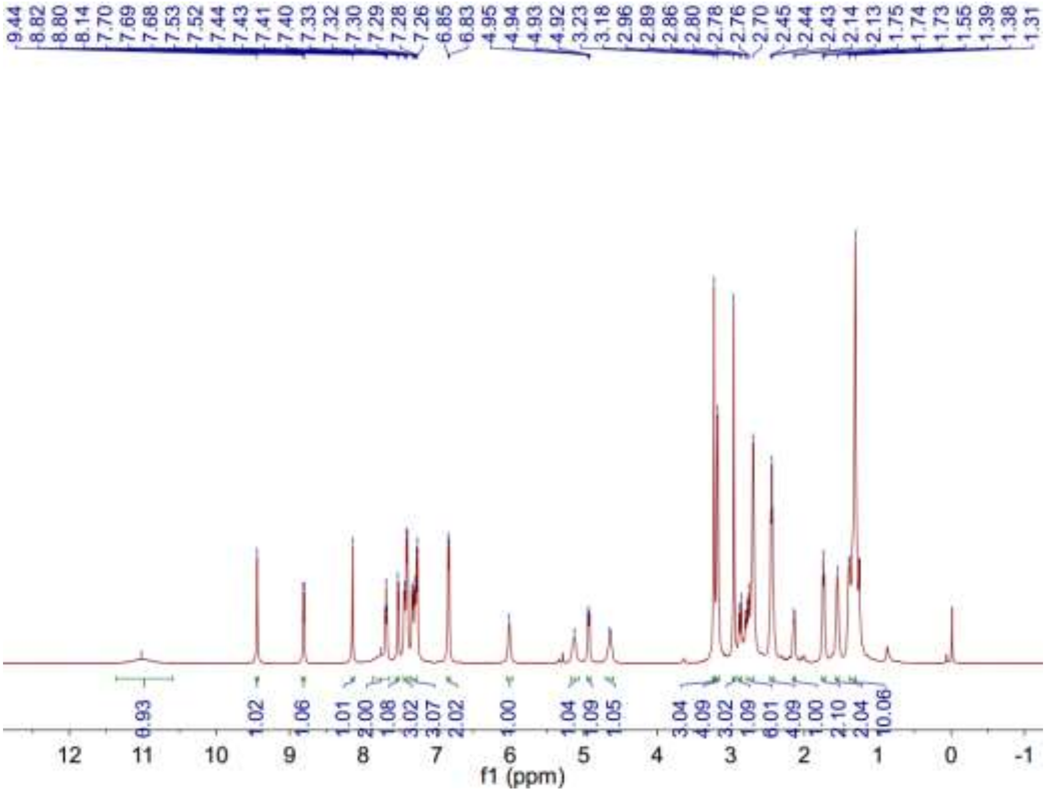

Figure S10. The ^{1}H-NMR spectrum of Compd. **A10**

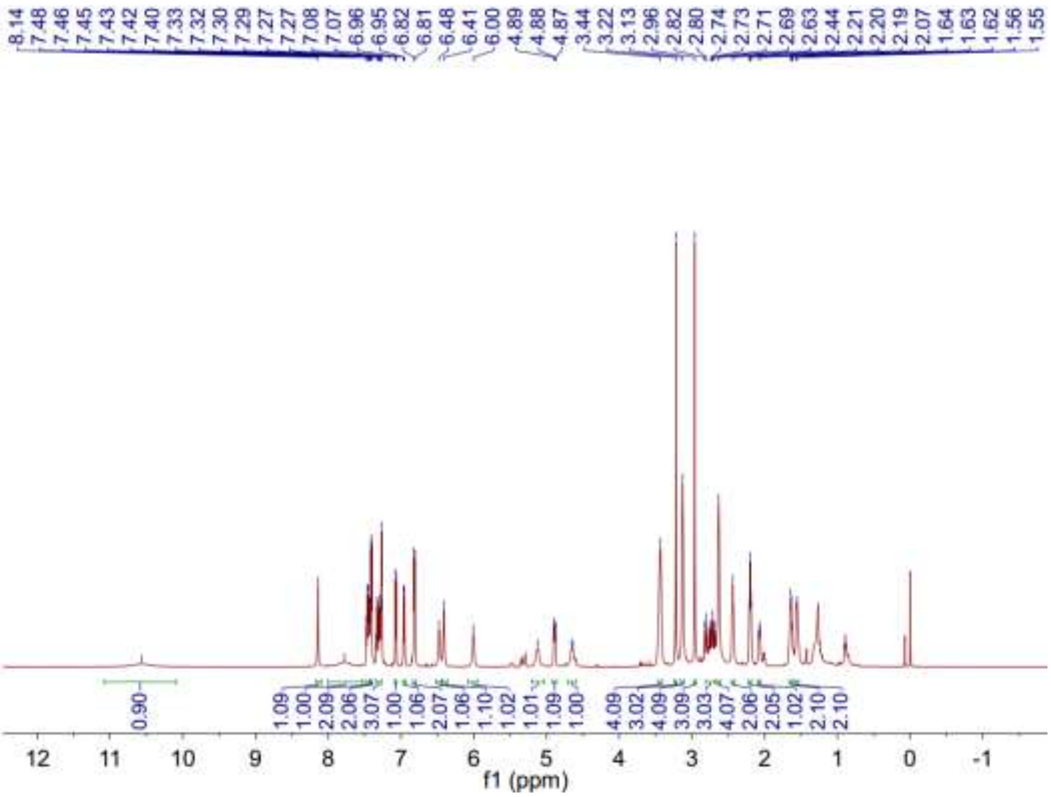

Figure S11. The ^{1}H-NMR spectrum of Compd. **A11**

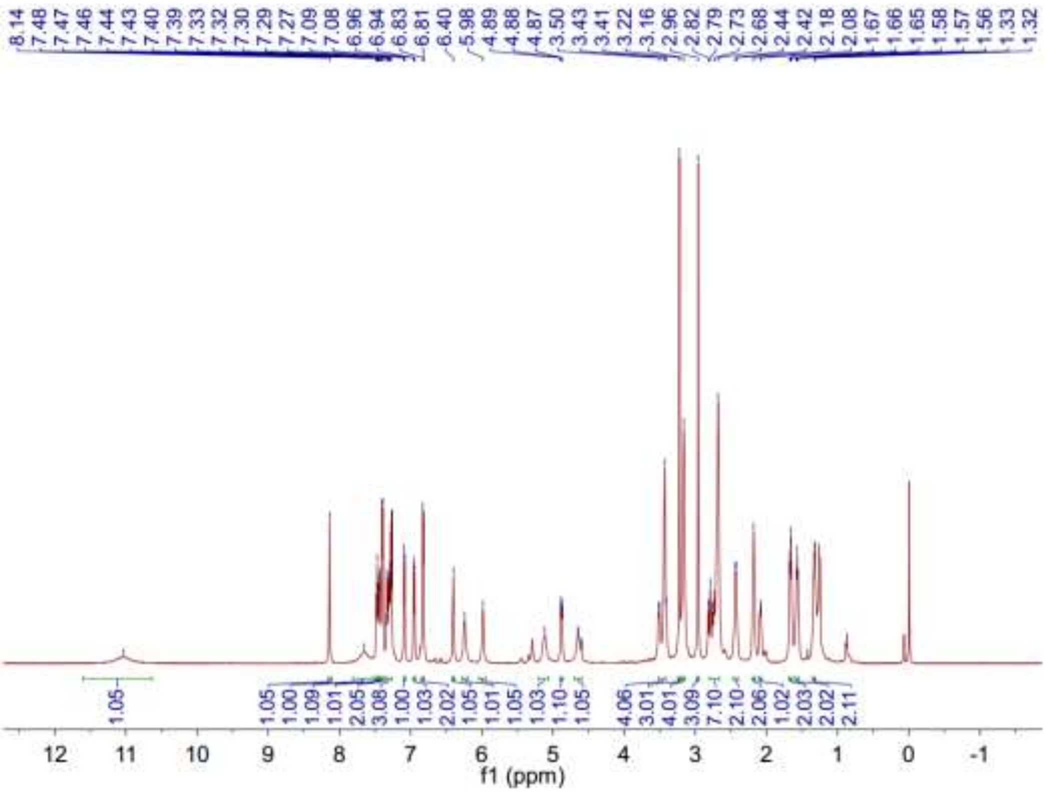

Figure S12. The ^{1}H-NMR spectrum of Compd. **A12**

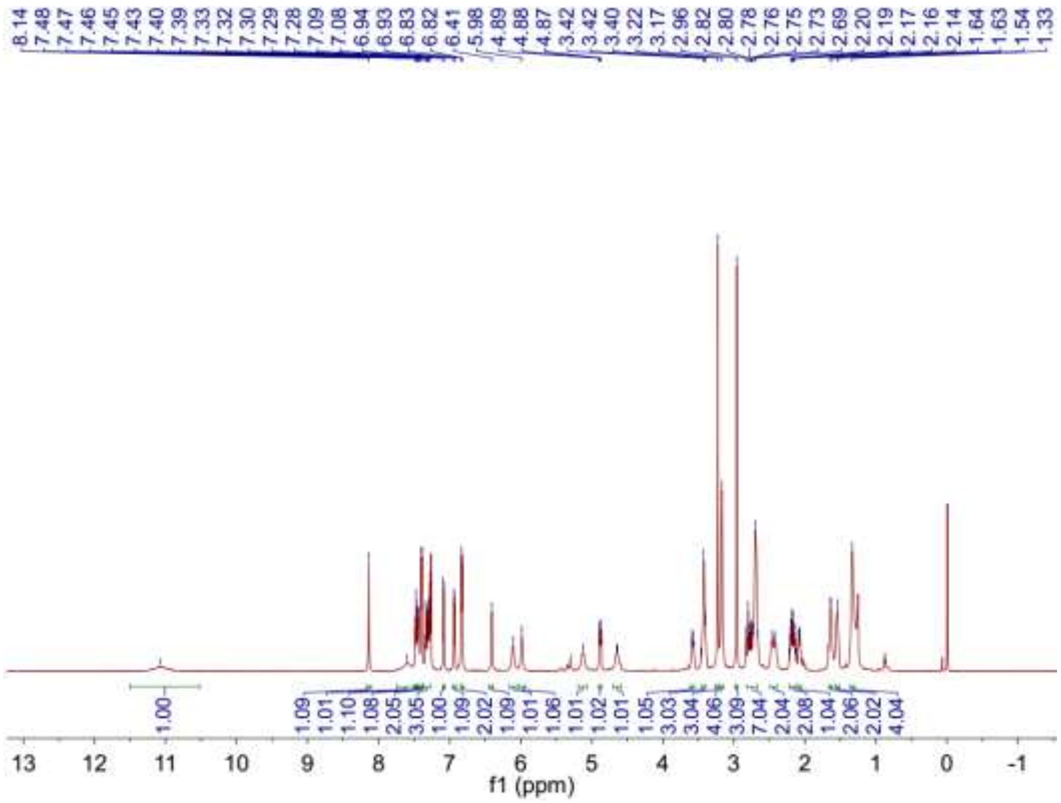

Figure S13. The ^{1}H-NMR spectrum of Compd. **A13**

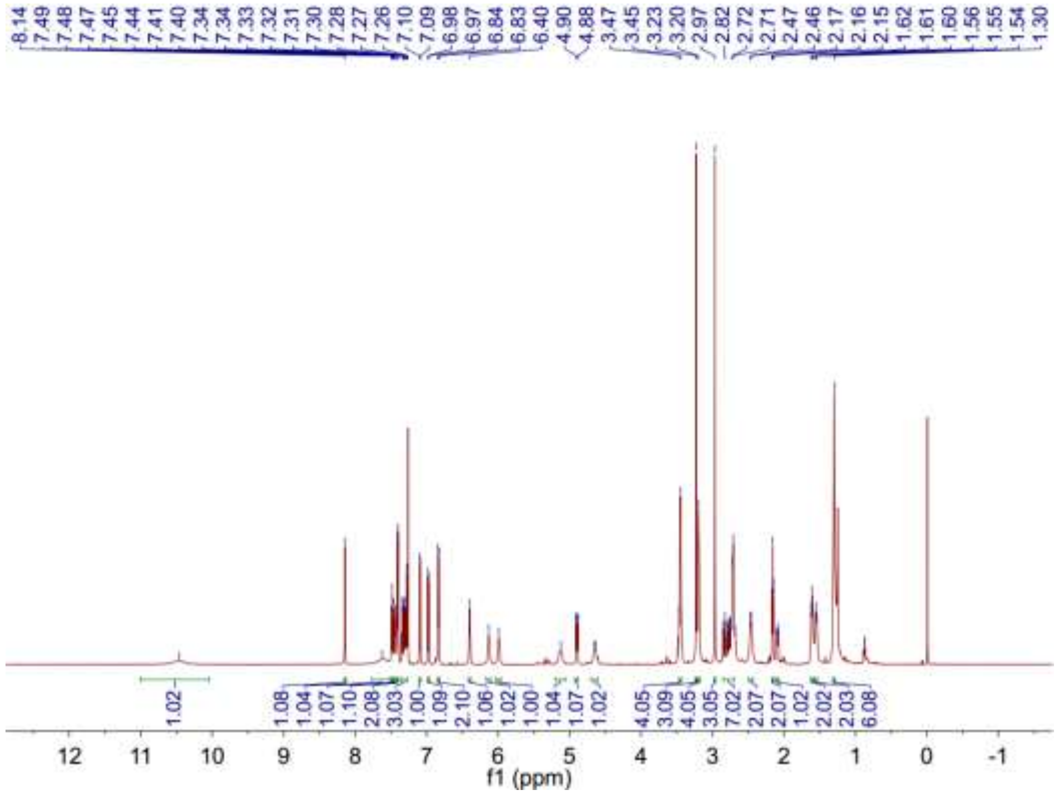

Figure S14. The ^{1}H-NMR spectrum of Compd. **A14**

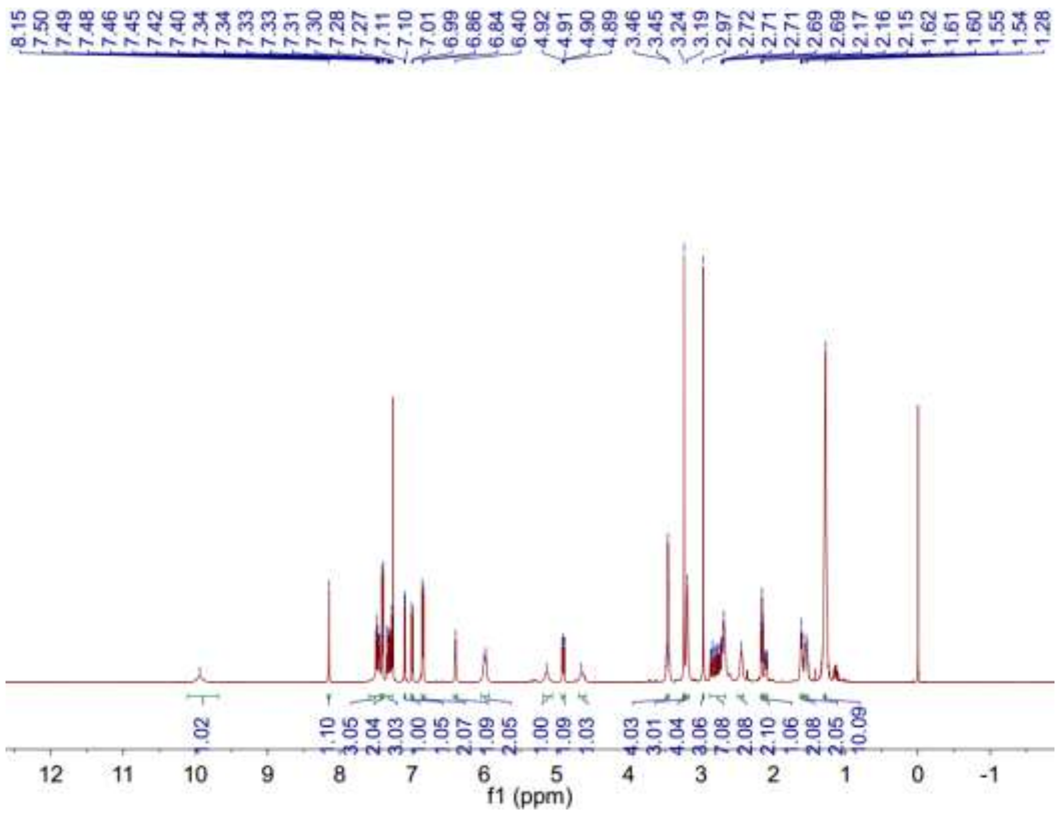

Figure S15. The ^{1}H-NMR spectrum of Compd. **A15**

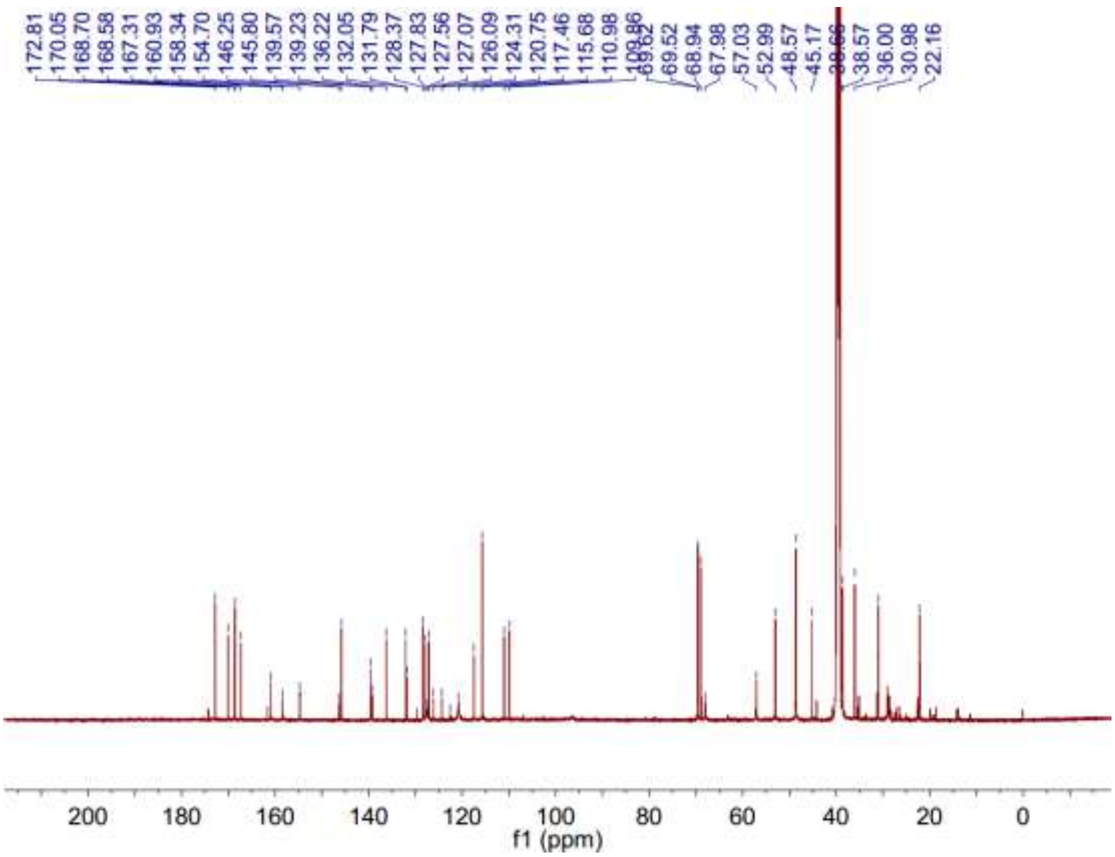

Figure S16. The ^{13}C-NMR spectrum of Compd. **A1**

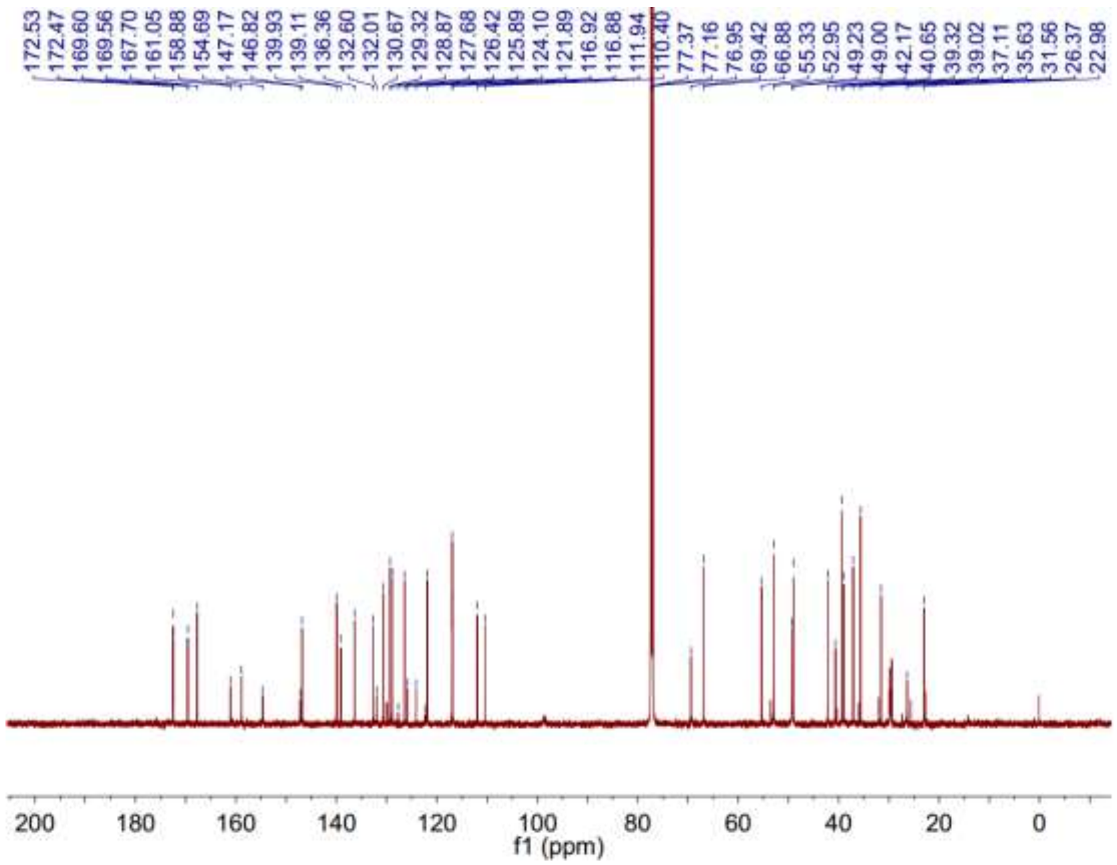

Figure S17. The ^{13}C-NMR spectrum of Compd. **A2**

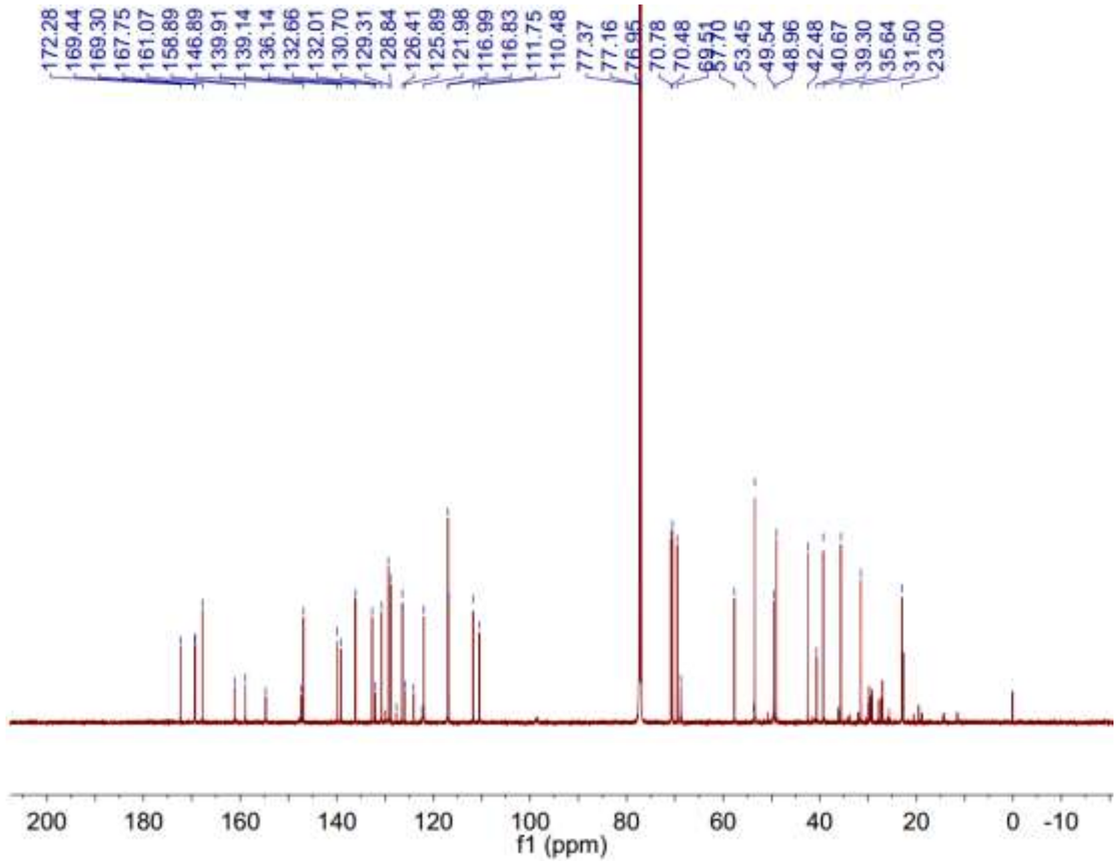

Figure S18. The ^{13}C-NMR spectrum of Compd. **A3**

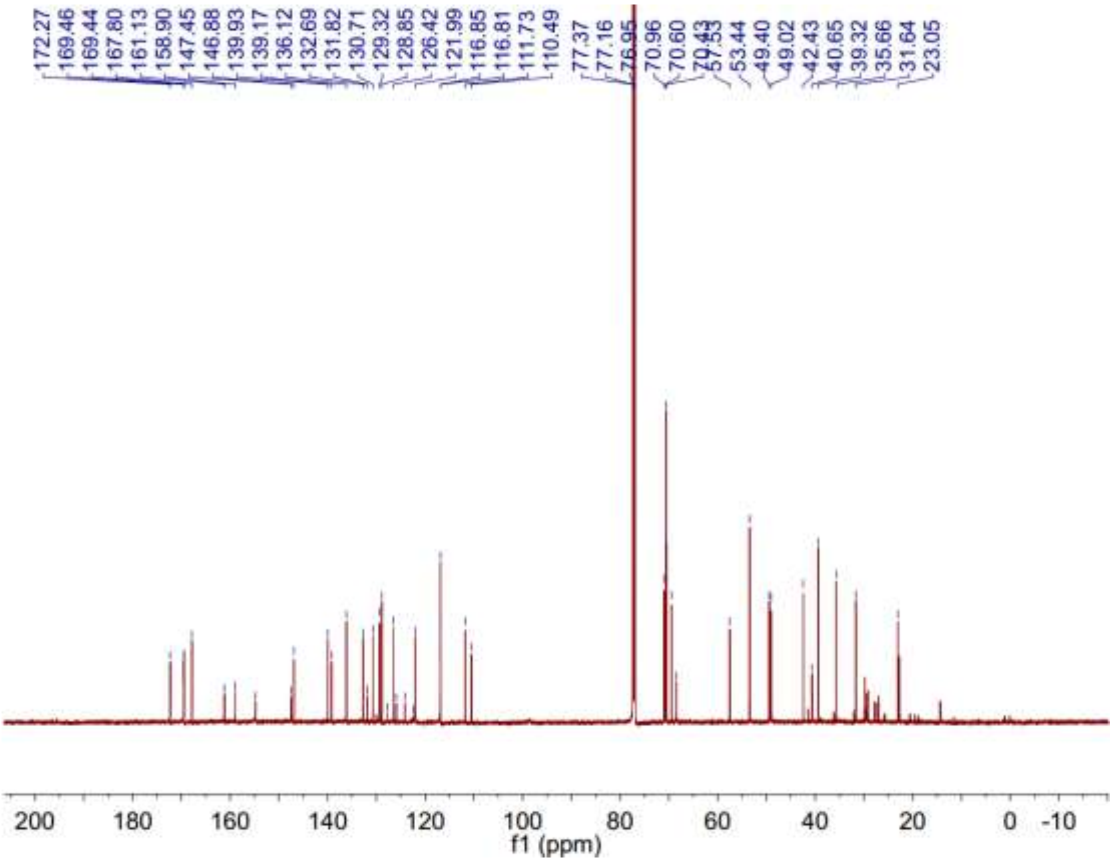

Figure S19. The ^{13}C-NMR spectrum of Compd. **A4**

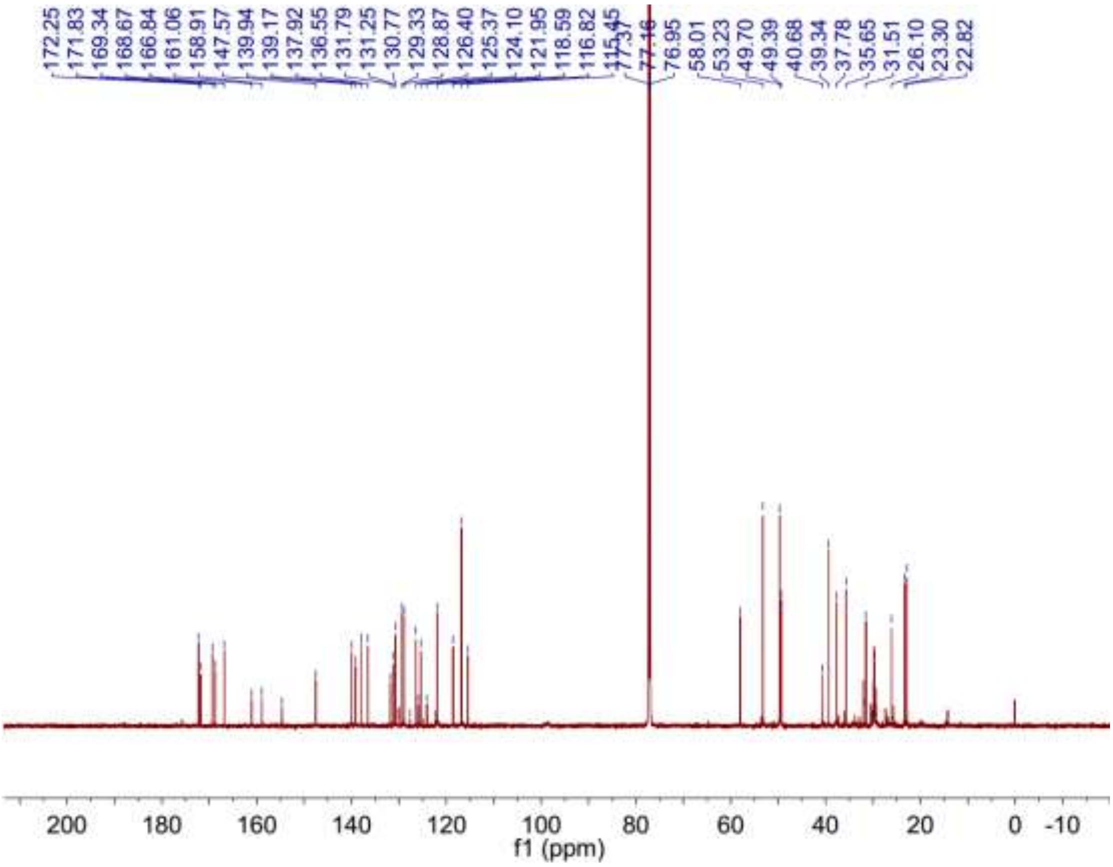

Figure S20. The ^{13}C-NMR spectrum of Compd. **A5**

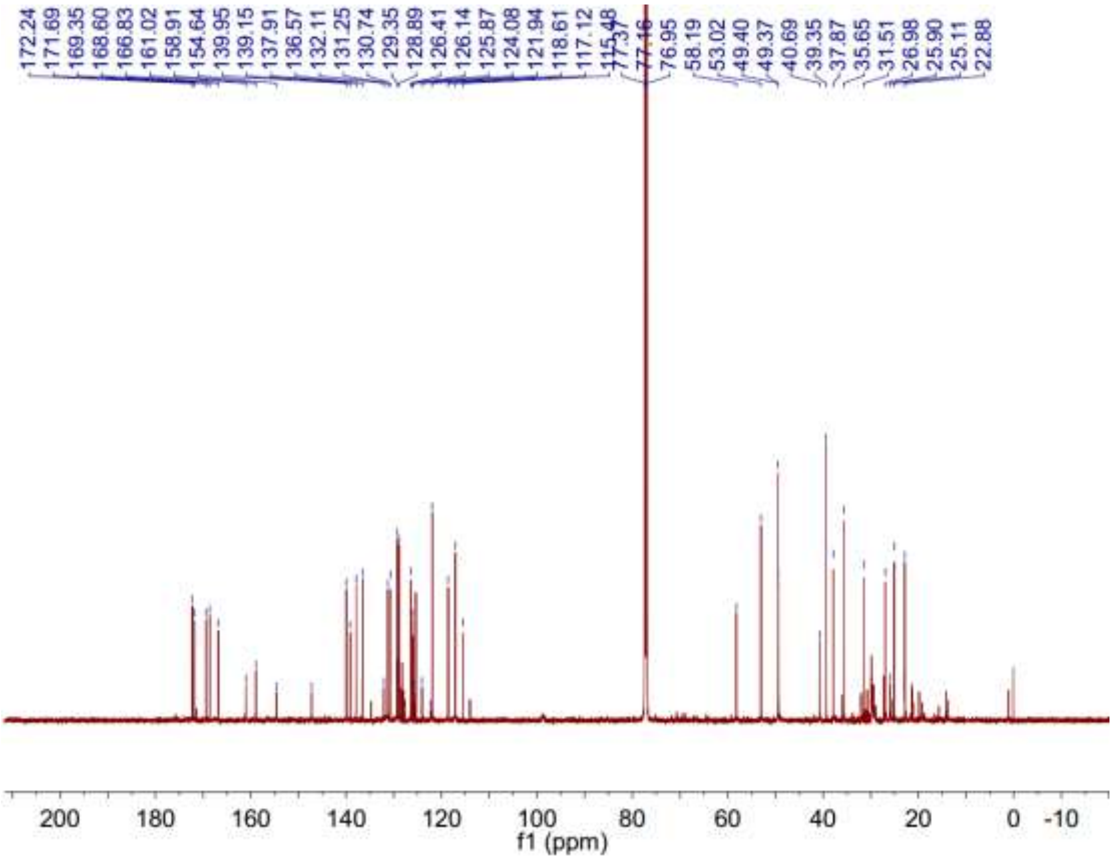

Figure S21. The ^{13}C-NMR spectrum of Compd. **A6**

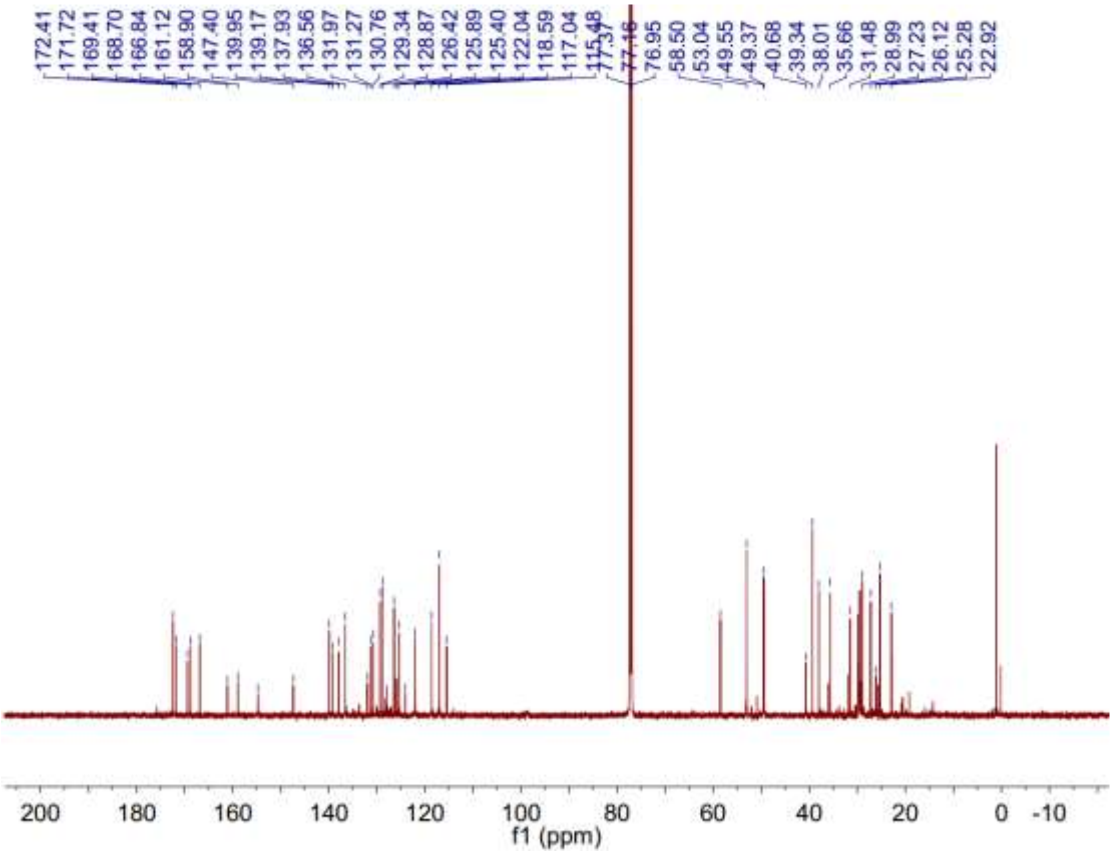

Figure S22. The ^{13}C-NMR spectrum of Compd. **A7**

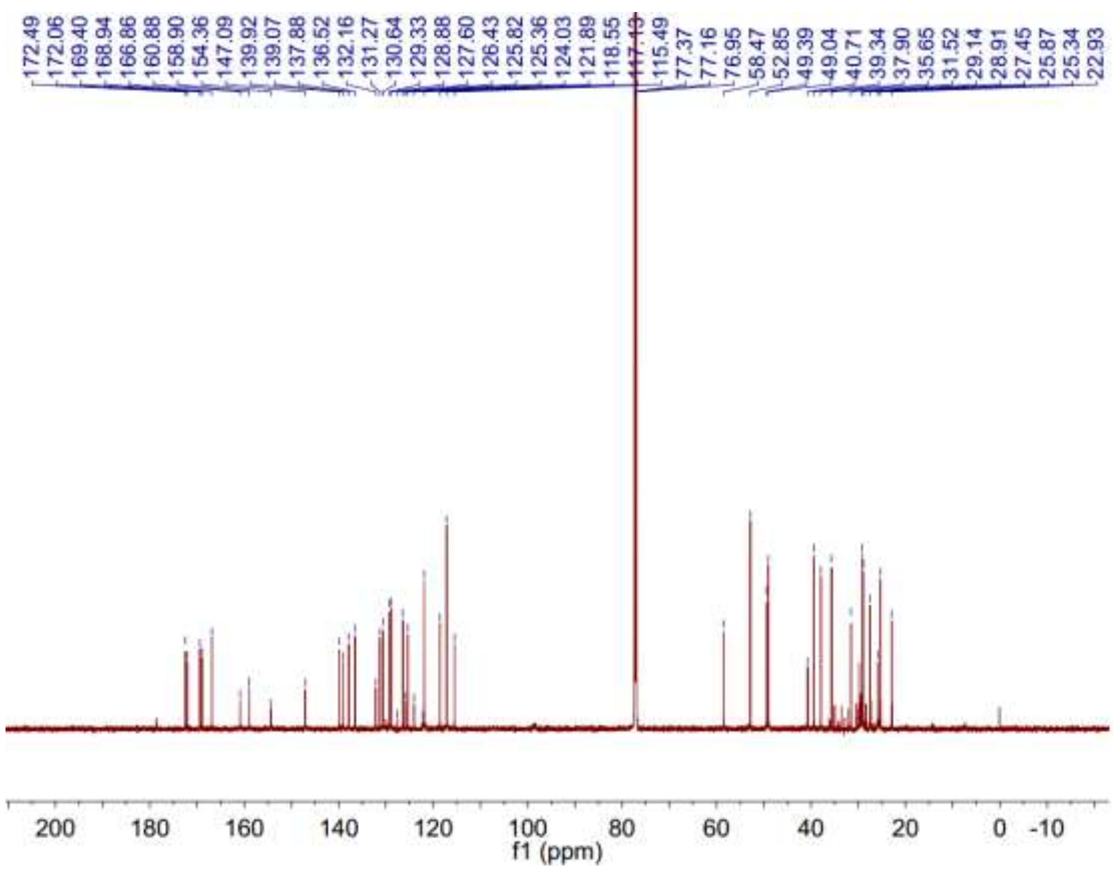

Figure S23. The ^{13}C-NMR spectrum of Compd. **A8**

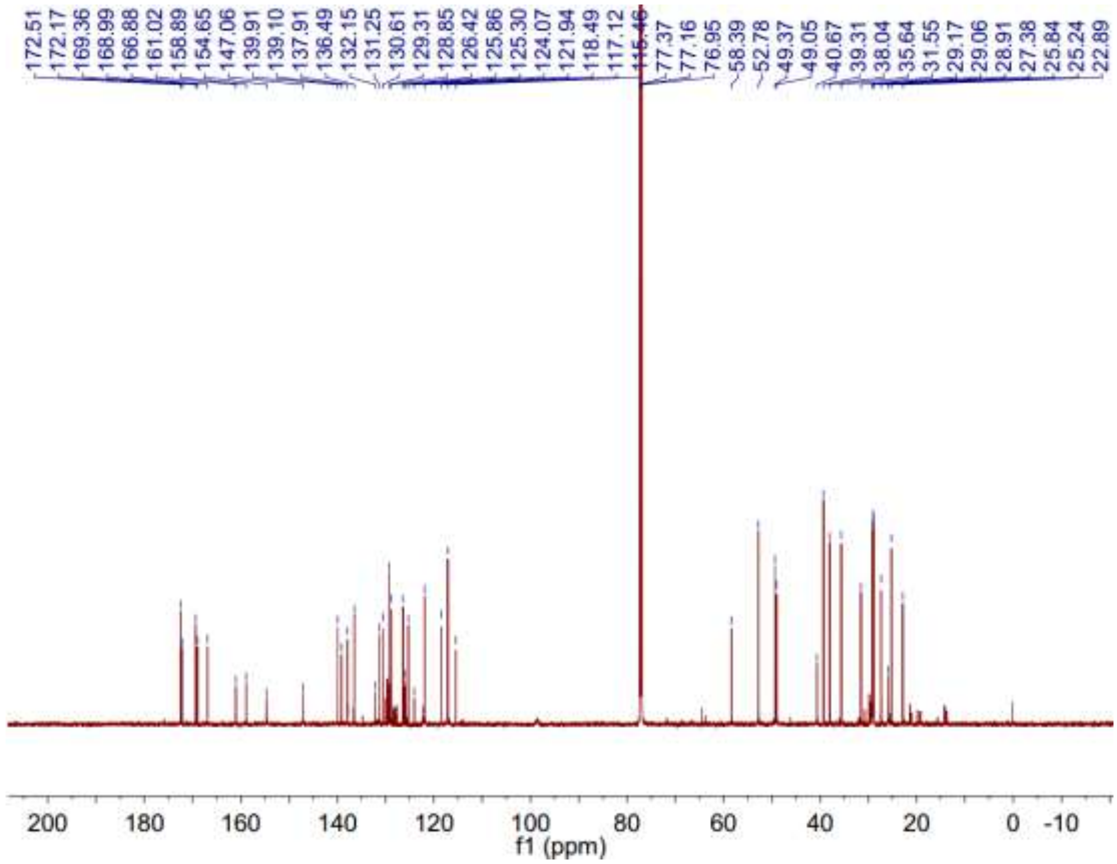

Figure S24. The ^{13}C-NMR spectrum of Compd. **A9**

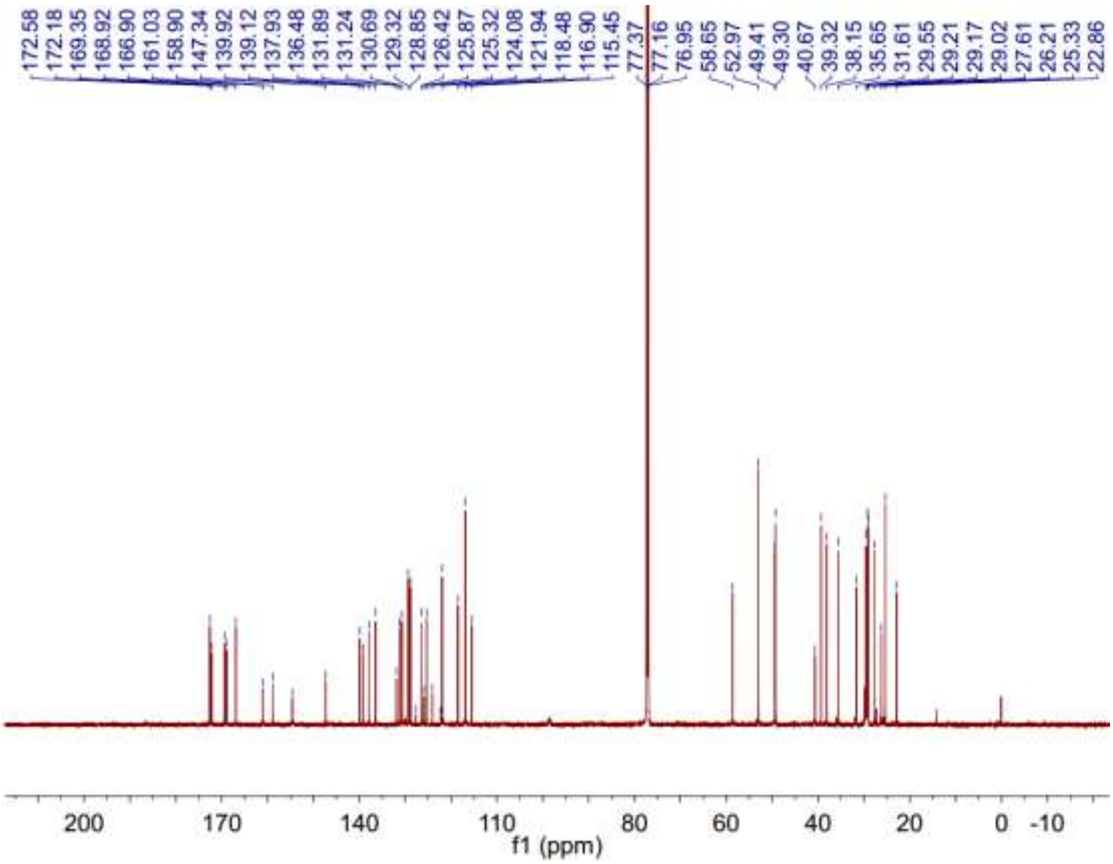

Figure S25. The ^{13}C-NMR spectrum of Compd. **A10**

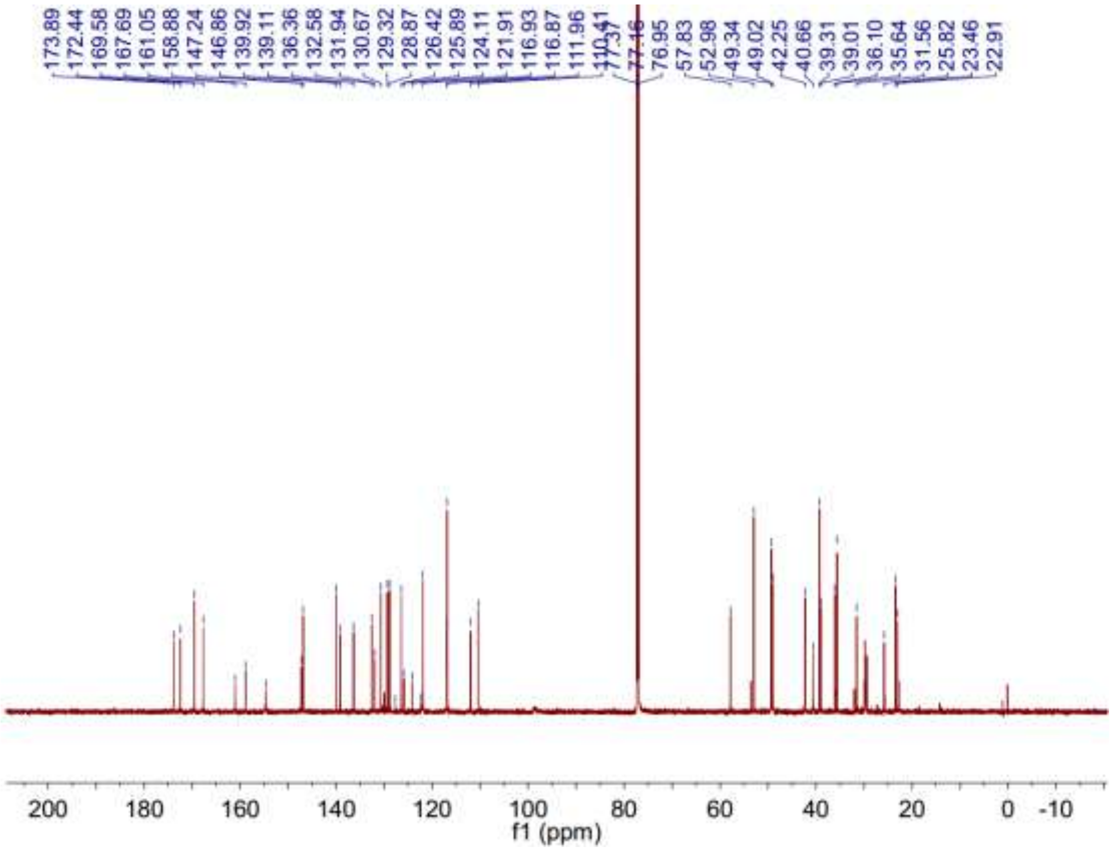

Figure S26. The ^{13}C-NMR spectrum of Compd. **A11**

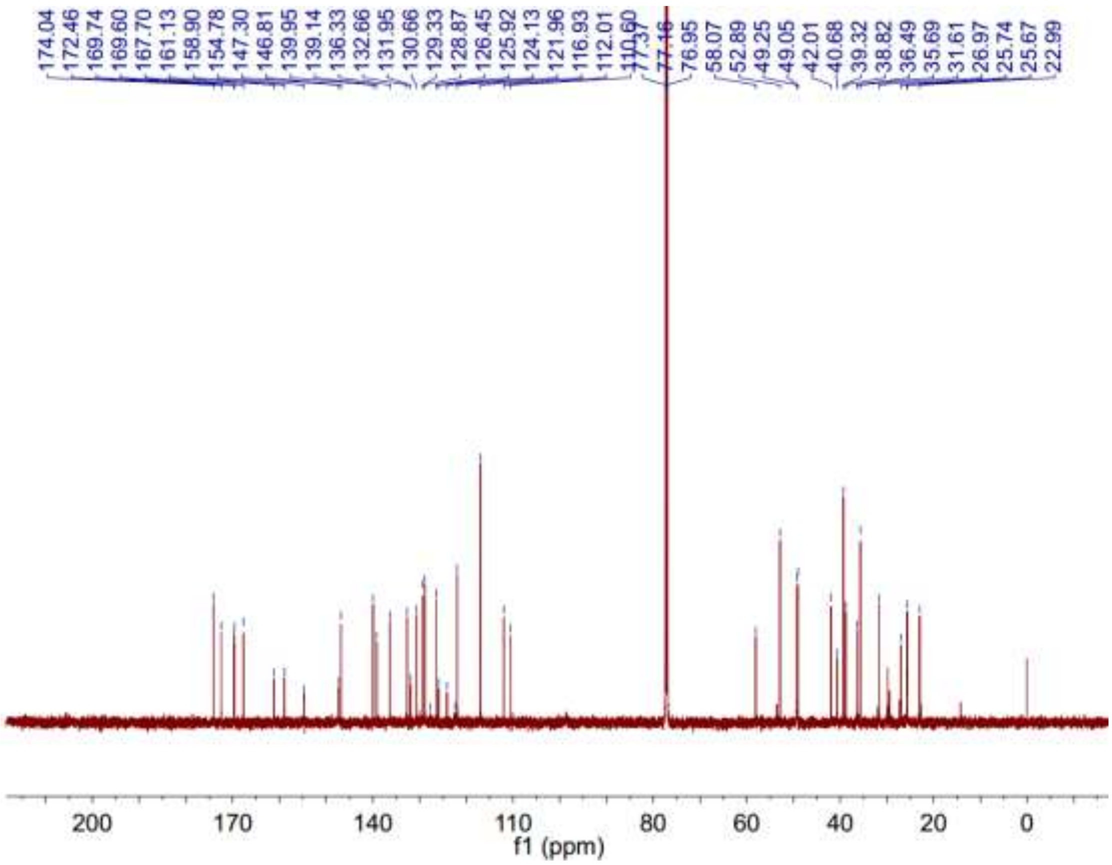

Figure S27. The ^{13}C-NMR spectrum of Compd. **A12**

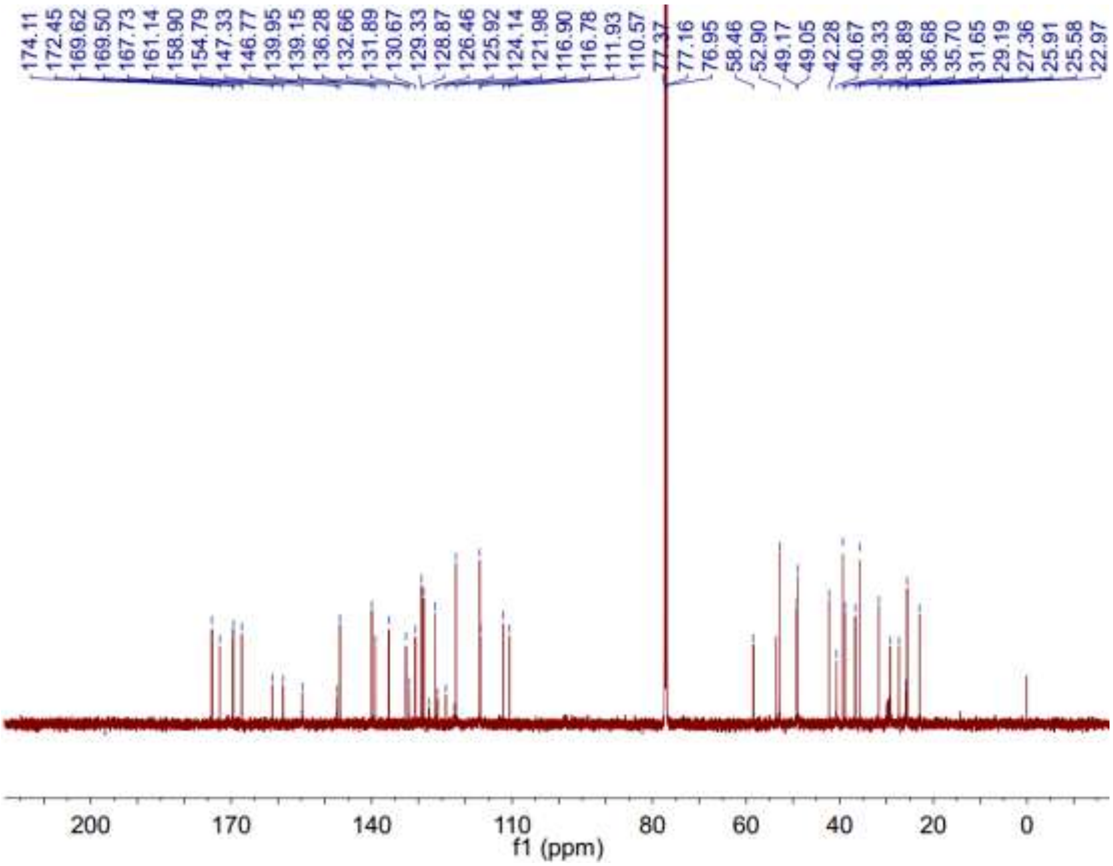

Figure S28. The ^{13}C-NMR spectrum of Compd. **A13**

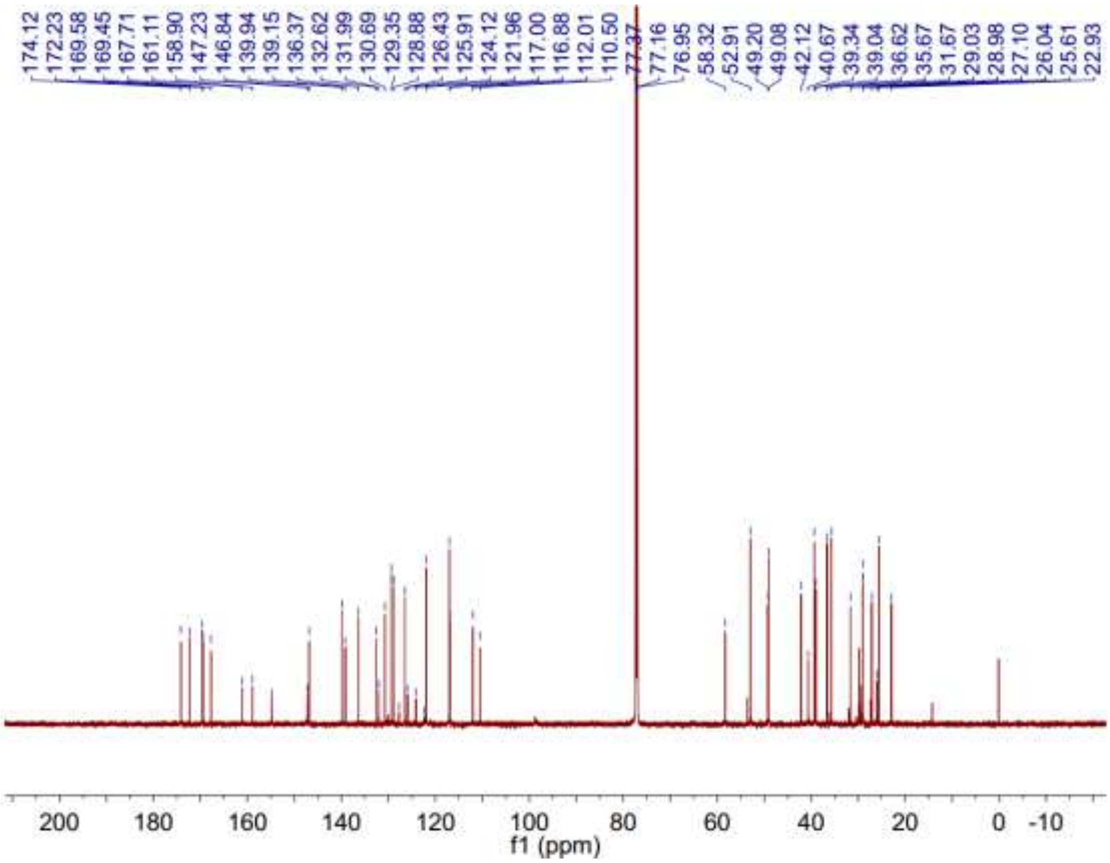

Figure S29. The ^{13}C-NMR spectrum of Compd. **A14**

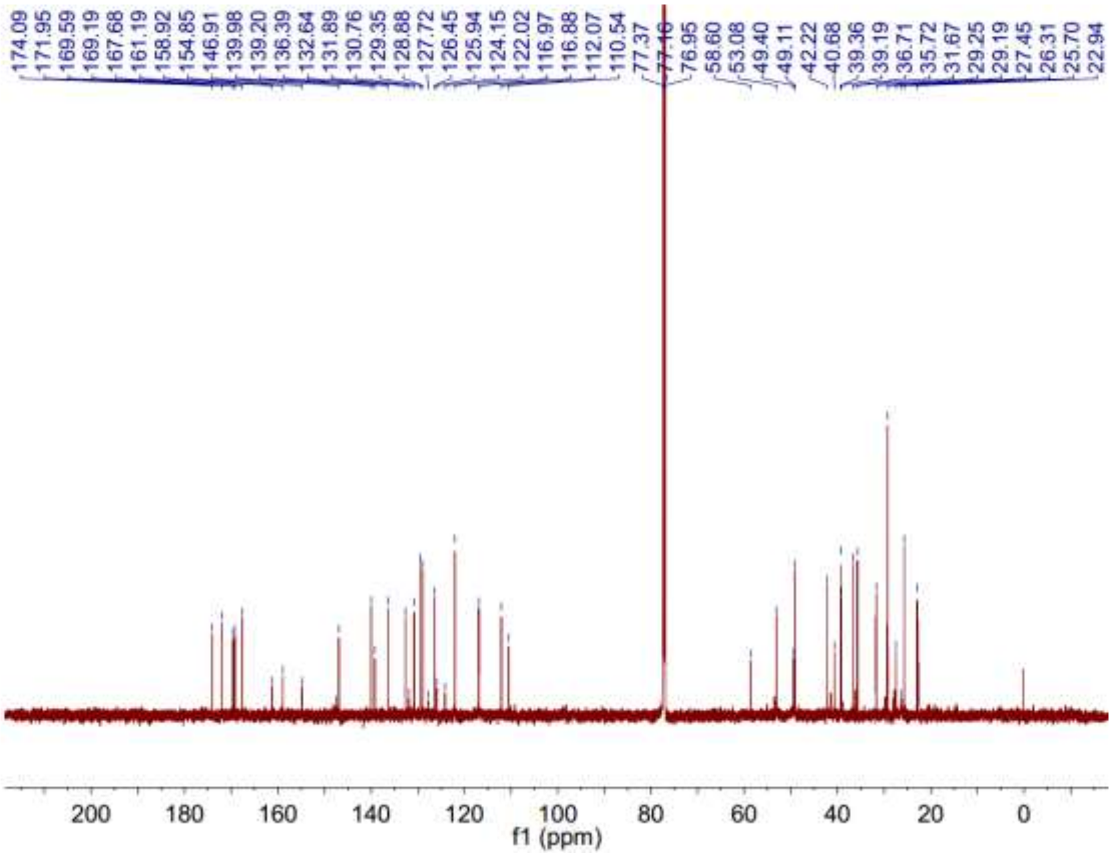

Figure S30. The ^{13}C-NMR spectrum of Compd. **A15**

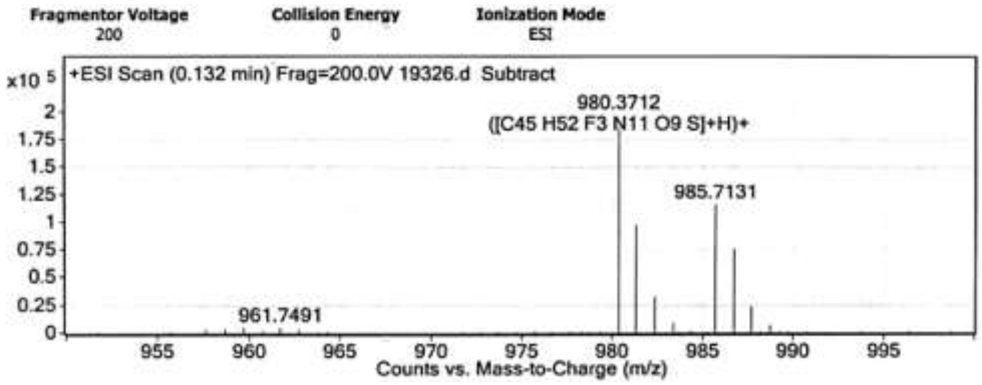

Figure S31. The HRMS spectrum of Compd. **A1**

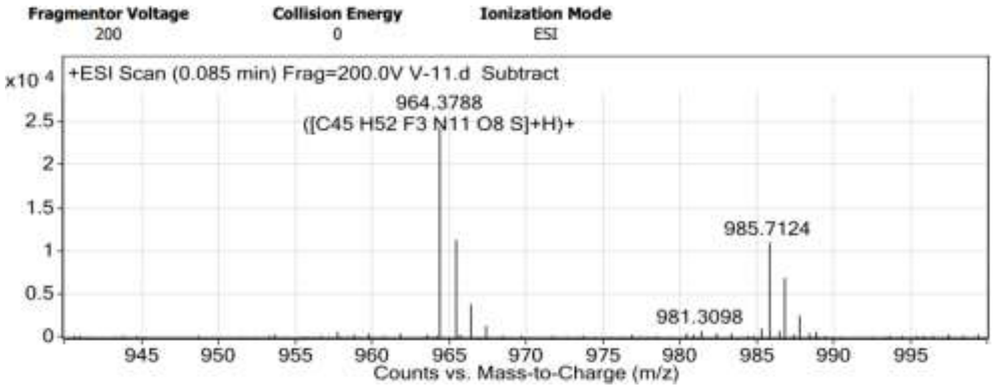

Figure S32. The HRMS spectrum of Compd. **A2**

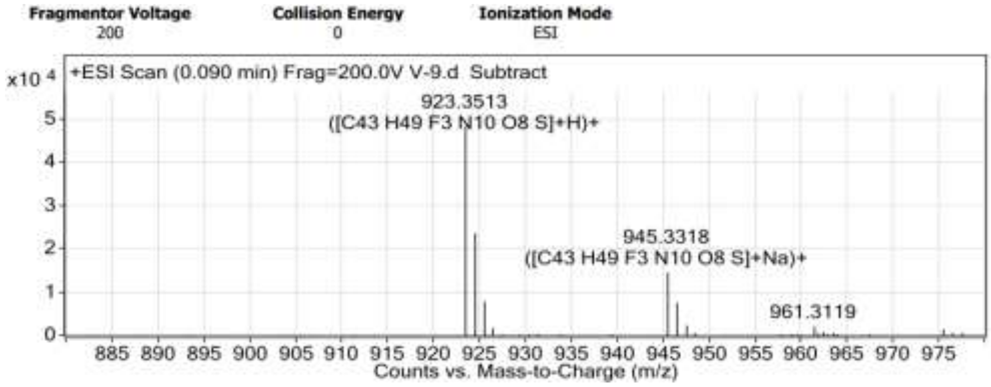

Figure S33. The HRMS spectrum of Compd. **A3**

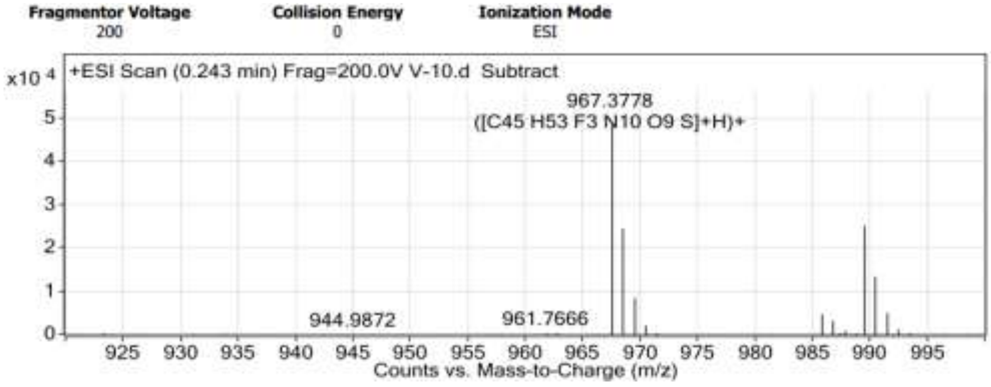

Figure S34. The HRMS spectrum of Compd. **A4**

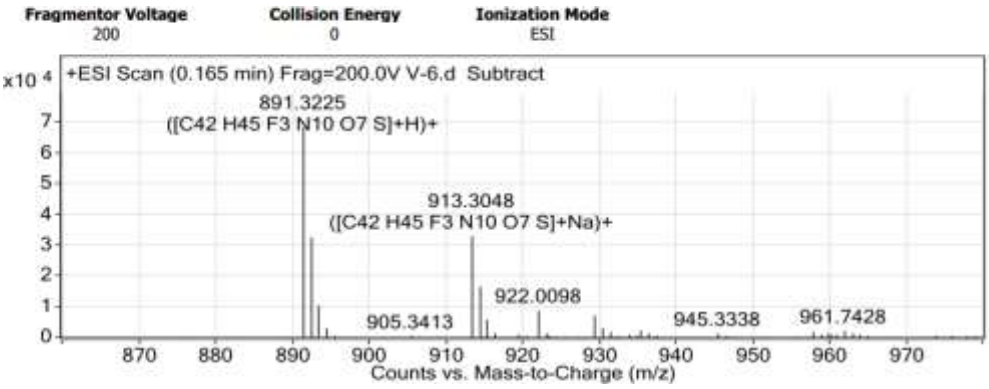

Figure S35. The HRMS spectrum of Compd. **A5**

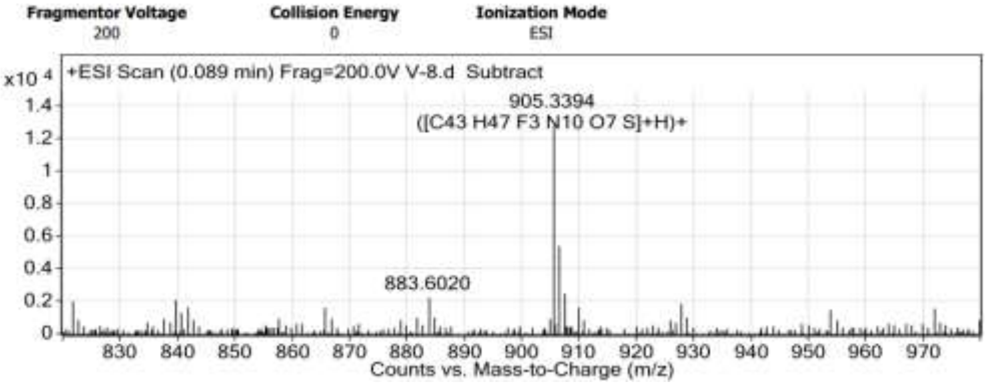

Figure S36. The HRMS spectrum of Compd. **A6**

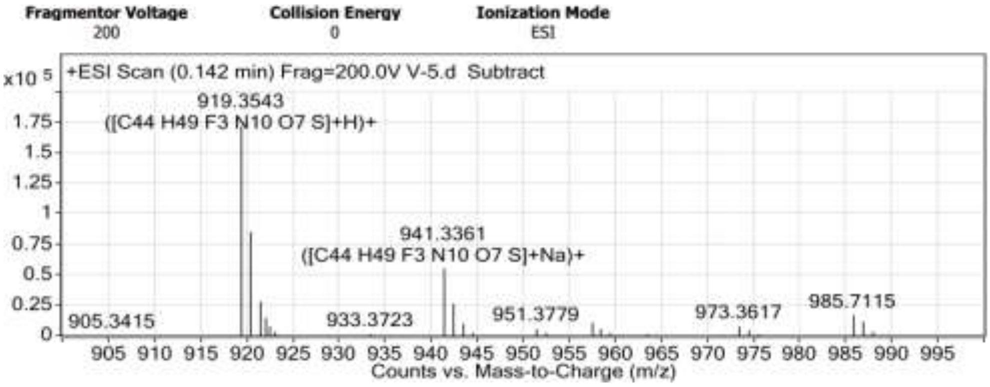

Figure S37. The HRMS spectrum of Compd. **A7**

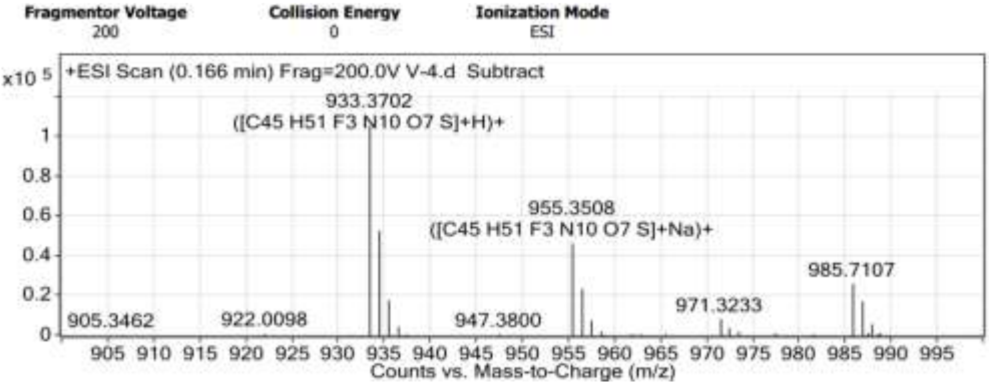

Figure S38. The HRMS spectrum of Compd. **A8**

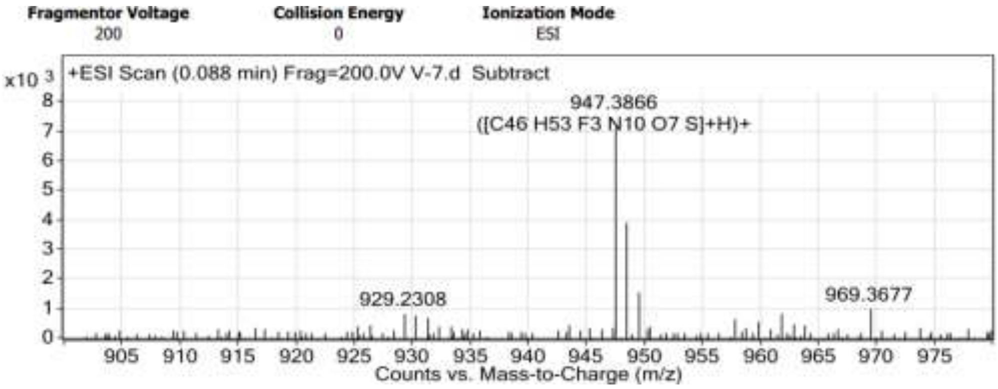

Figure S39. The HRMS spectrum of Compd. **A9**

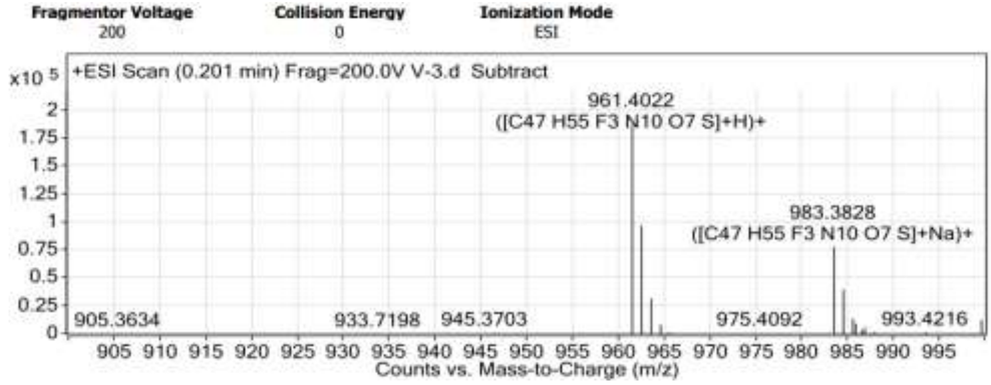

Figure S40. The HRMS spectrum of Compd. **A10**

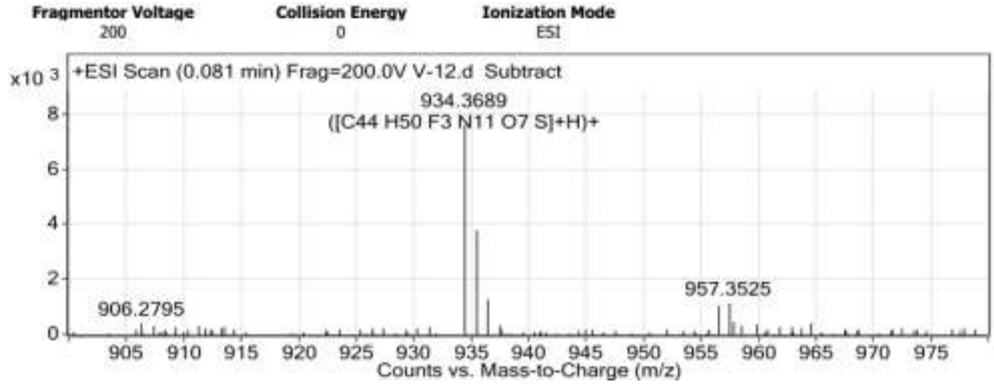

Figure S41. The HRMS spectrum of Compd. **A11**

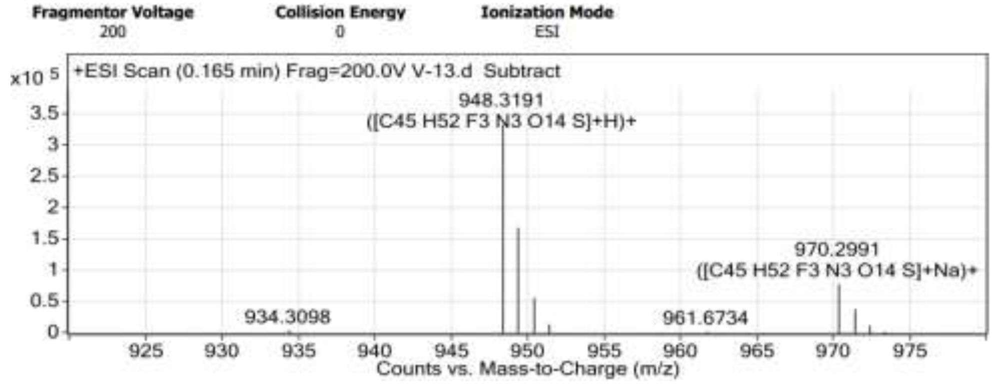

Figure S42. The HRMS spectrum of Compd. **A12**

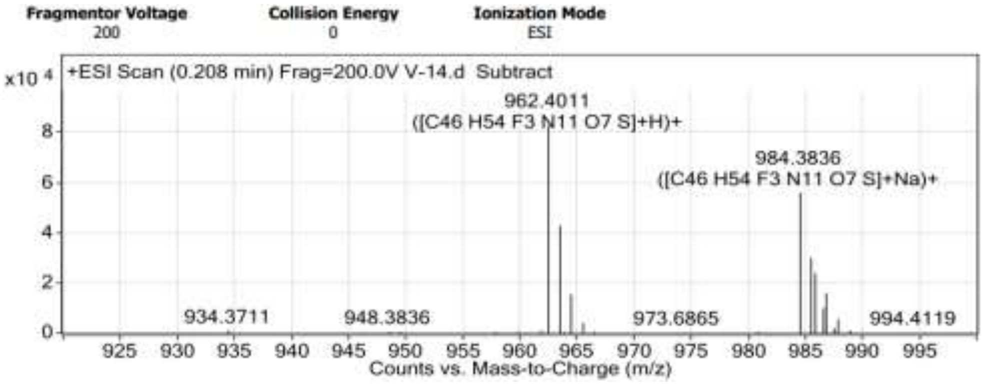

Figure S43. The HRMS spectrum of Compd. **A13**

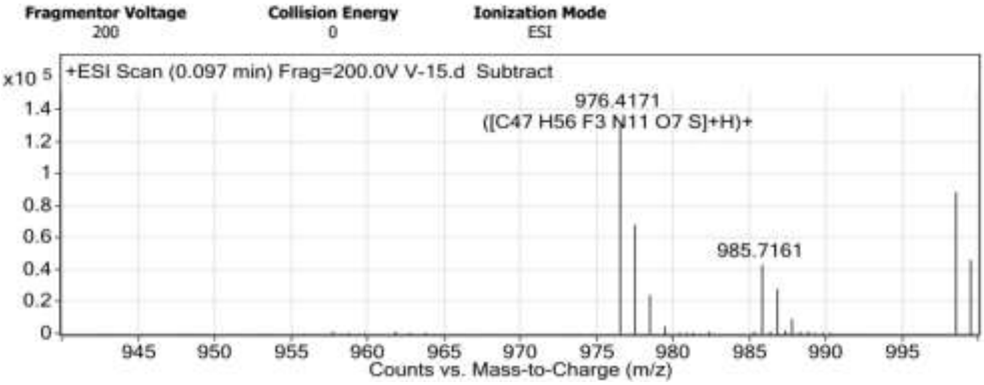

Figure S44. The HRMS spectrum of Compd. **A14**

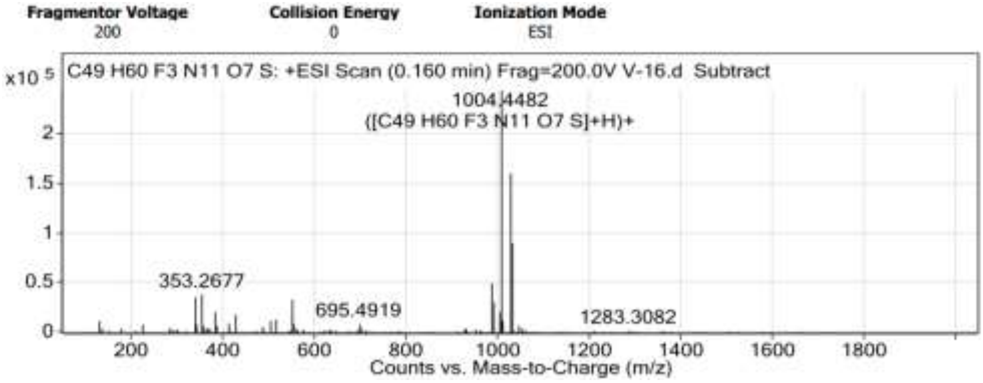

Figure S45. The HRMS spectrum of Compd. **A15**

We split compound **A13** into two parts for docking. Firstly, we docked the FAK ligand-linker moiety with the FAK protein. Molecular docking studies showed that the 2,4-diaminopyrimidine core formed two donor-acceptor interactions with Cys502 in the hinge region, *N*-methyl sulfonamide fragment formed a hydrogen bond interaction with Asp564 in the DFG-motif, the trifluoromethyl group contained a hydrophobic interaction with the gatekeeper residue Met499, the linker extended from the solvent region to the outside of the protein pocket (Fig. S46). Secondly, we docked the Pomalidomide-linker moiety with the CRBN protein. Molecular docking studies showed that piperidine-2,6-dione formed three donor-acceptor interactions with His380 and Trp382, the linker also extended from the solvent region to the outside of the protein pocket (Fig. S47).

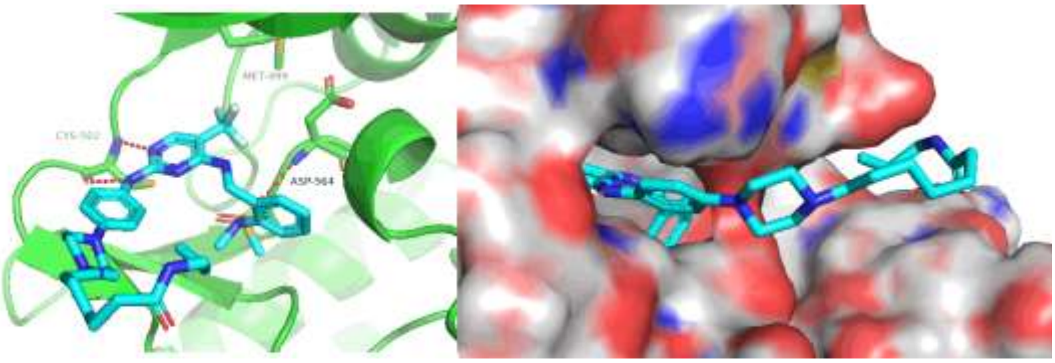

Fig. S46 Molecular docking model of FAK ligand-linker moiety of compound **A13** with FAK protein (PDB ID: 3BZ3)

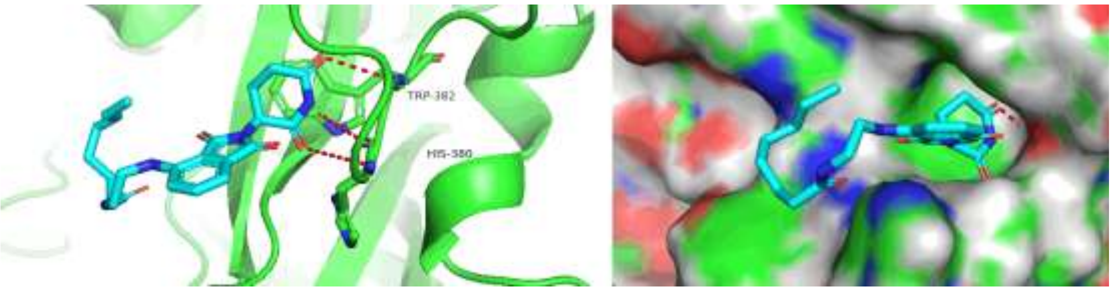

Fig. S47 Molecular docking model of pomalidomide-linker moiety of compound **A13** with CRBN protein (PDB ID: 4CI3)
